# An Insight into Anion Extraction by Amphiphiles: Hydrophobic Microenvironments as a Requirement for the Extractant Selectivity

**DOI:** 10.1021/acsomega.3c06767

**Published:** 2023-11-03

**Authors:** Karolína Salvadori, Alessia Onali, Gregory Mathez, Václav Eigner, Marcela Dendisová, Pavel Matějka, Monika Mullerová, Andrea Brancale, Petra Cuřínová

**Affiliations:** † Department of Physical Chemistry, University of Chemistry and Technology Prague, Technická 5, Prague 6 16628, Czech Republic; ‡ Department of Bioorganic Chemistry and Biomaterials, Institute of Chemical Process Fundamentals of the CAS, v.v.i., Rozvojová 135, Prague 6 16502, Czech Republic; § Department of Organic Chemistry, University of Chemistry and Technology Prague, Technická 5, Prague 6 16628, Czech Republic; ∥ Department of Solid-State Chemistry, University of Chemistry and Technology Prague, Technická 5, Prague 6 16628, Czech Republic

## Abstract

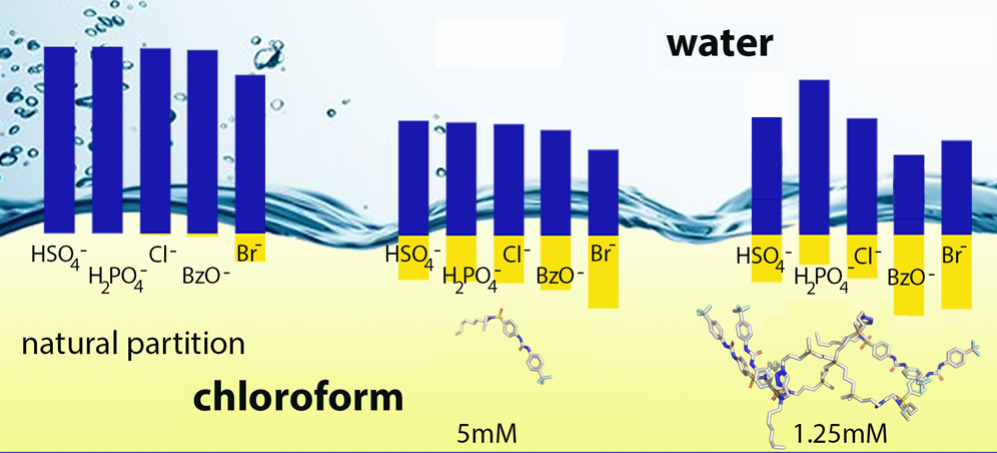

Coupling of electron-deficient urea units with aliphatic chains gives rise to amphiphilic compounds that bind to phosphate and benzoate anions in the hydrogen bonding competitive solvent (DMSO) with *K*_Ass_ = 6 580 M^–1^ and *K*_Ass_ = 4 100 M^–1^, respectively. The anchoring of these receptor moieties to the dendritic support does not result in a loss of anion binding and enables new applications. Due to the formation of a microenvironment in the dendrimer, the high selectivity of the prepared compound toward benzoate is maintained even in the presence of aqueous media during extraction experiments. In the presence of binding sites at 5 mM concentration, the amount of benzoate corresponding to the full binding site occupancy is transferred into the chloroform phase from its 10 mM aqueous solution. A thorough investigation of the extraction behavior of the dendrimer reported here, supported by a series of molecular dynamics simulations, provides new insight into the fundamental principles of extraction of inorganic anions by amphiphiles.

## Introduction

The transport of oxo-anions from water to nonpolar media facilitated by synthetic extractants represents an important topic in modern supramolecular chemistry. Although many effective and selective anion receptors are already known,^1−6^ only a few have been used in extraction experiments.^7^ In contact with the aqueous phase, both the efficiency and selectivity of the extractant are compromised, and the natural lipophilicity of anions predominates; as a result, oxo-anions extraction becomes a challenge.^8^

The Hofmeister series classifies anions according to their ability to salt out proteins, an aptitude that is considered to be closely related to their hydrophilicity.^9^ The extraction of anions into nonpolar solvents follows the Hofmeister bias, and the transport of highly hydrated anions as sulfates, phosphates, and carboxylates into the organic phase becomes difficult. On the other hand, altering the order in the Hofmeister series is highly desirable, as it can lead to systems that are able to remove pollutants from water bodies or recycle valuable materials. For this reason, despite the complexity of the task, the design and synthesis of oxo-anion extractants represent a highly scientifically relevant topic in supramolecular chemistry.

Regarding inorganic phosphates, many selective and effective organic receptors are known.^10,11^ However, only a few attempts have been made to extract phosphates into an organic phase. The ruthenium complex-based urea derivatives, which inherently offer the counterion to be soluble in the organic phase, were used to transfer dihydrogen phosphate from water into chloroform. Although some deprotonation of the receptor urea group by TBA^+^H_2_PO_4_^–^ was observed, the systems could transport dihydrogen phosphate into the organic phase with about 30% efficiency.^12^ Tripodal thioureas were used to remove phosphates from aqueous media by precipitation in self-assembled capsules.^13^ Other tripodal thioureas were used in the liquid–liquid extraction experiments. Up to 55 mol % of the dihydrogen phosphate was extracted into chloroform from its 10 mM aqueous solution, with part of the phosphate being extracted and converted to its monobasic form.^14^ The pH-switchable phosphate binding was observed in a gadolinium-based receptor system that was used 10 times in “catch and release” extraction experiments.^15^ Preorganization of binding units on a calix[4]arene scaffold led to a receptor capable of extracting both phosphates and fluorides.^16^ Schiff-base-based macrocycles were polymerized to obtain a phosphate binding polymer, but it unfortunately had a low phosphate extraction ability.^17^

Sulfate can be extracted from water, by extractants such as calixpyrrole derivatives,^18,19^ cyclic amides,^20^ and iminoguanidinium-based compounds.^21^ The presence of Aliquat 336 greatly enhances the extraction efficiency. A tripodal urea-based receptor working in the presence of TBA^+^Cl^–^ extracted sulfate into chloroform via the formation of 12 hydrogen bonds.^22^

Carboxylates obtained by fermentation processes are a valuable starting material for biopolymer preparation. Their extraction from aqueous streams is mainly based on pH changes or the formation of salts with lipophilic bases.^23−25^ However, successful attempts on the selective extraction of dicarboxylates were achieved using a dicopper-cryptate.^26^

We previously synthesized recyclable dendritic receptors suitable for dihydrogen phosphate transport through dialysis membranes.^27^ Moreover, we prepared and studied anchorable receptor moieties capable of dihydrogen phosphate recognition, by linking urea binding sites with sulfonamidic groups.^28^ In the present work, we aim to anchor these moieties to a dendritic core, thus obtaining a bulky recyclable host with sulfonamide-urea binding sites. These binding sites, having a superior affinity toward dihydrogen phosphate and carboxylates, should make the resulting material suitable for liquid–liquid extraction of oxo-anions. Moreover, a series of molecular simulations gave us novel insight into the behavior of the anions and the receptors at the water–chloroform interface.

## Results and Discussion

### Synthesis

**Scheme 1:**
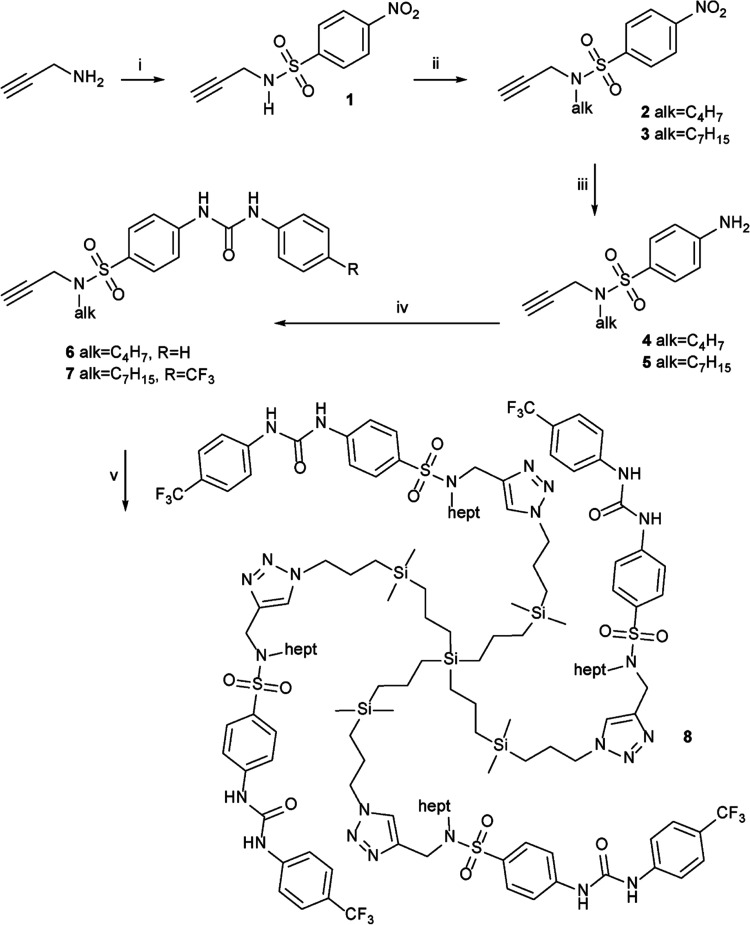
Synthesis of Receptors **6** and **7** Followed by Anchoring of **7** to the Dendritic Core upon Formation of **8** (i) nosyl chloride, pyridine, r.t.; (ii) aq. NaOH, lyophilization, then alkyl halogenide in acetonitrile, r.t.; (iii) SnCl_2_ × 2H_2_O, ethanol, reflux; (iv) aryl isocyanate, dichloromethane, r.t.; (v) CuI, DIPEA, Si(CH_2_CH_2_CH_2_Si(CH_3_)_2_CH_2_CH_2_CH_2_N_3_)_4_, DMF, microwave, 150 °C.

The synthesis (Scheme 1) started from propargylamine, which was converted to sulfonamide **1** by reacting with nosyl chloride in pyridine with 95% yield. The addition of one equivalent of sodium hydroxide transformed **1** into a salt that was alkylated by alkyl bromide, forming compound **2** or **3** in 80% yields over two steps. The reduction to the corresponding amines **4** and **5** was performed using tin(II) chloride dihydrate in ethanol, which is inert to the triple bond and reduced the nitro groups almost quantitatively. The addition of **4** to phenyl isocyanate gave compound **6** in 65% yield. Compound **7** was obtained in 60% yield upon the addition of amine **5** to *p*-trifluoromethyl phenylisocyanate. Receptor moieties of **7** were then clicked under microwave irradiation to tetrakis[3-((3-azidopropyl)dimethylsilyl)propyl]silane, giving compound **8** in 75% yield.

### Solid State Study

**Figure 1:**
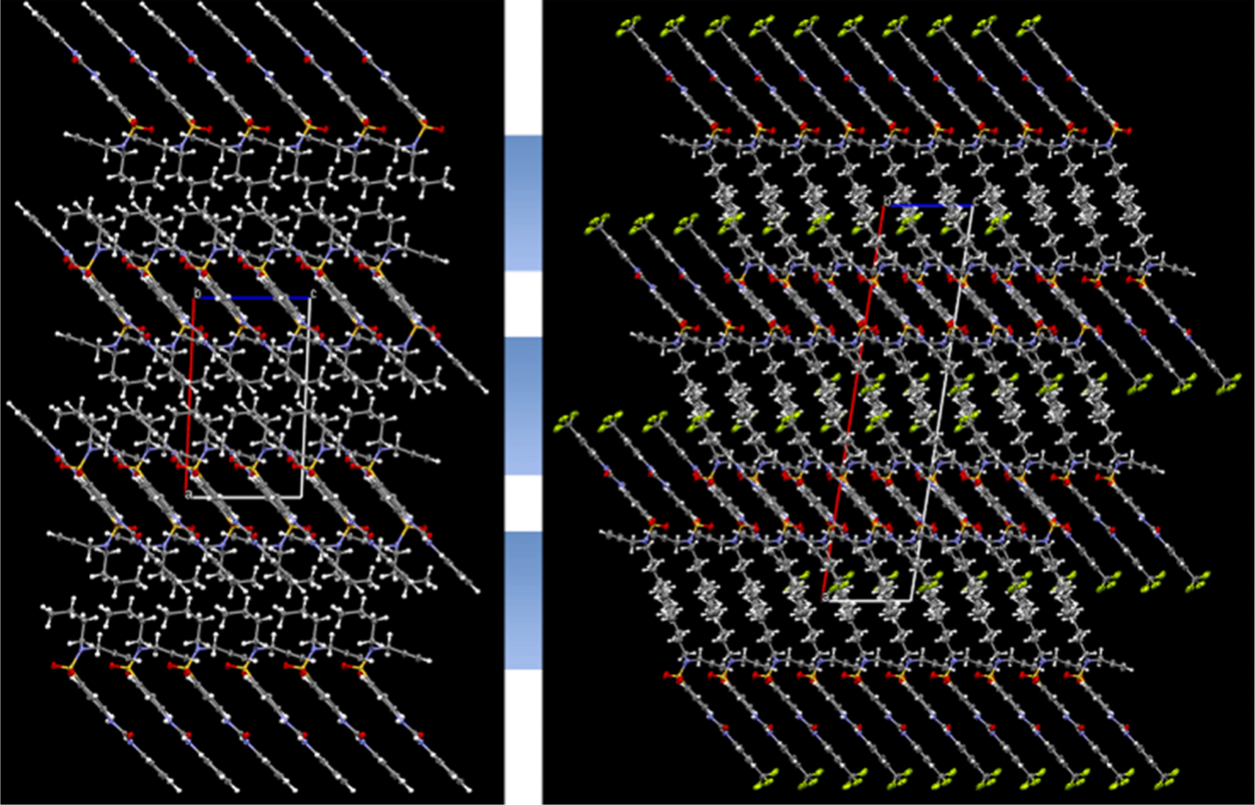
X-ray structures of **6** and **7**.

For both unanchored receptor moieties, compounds **6** and **7**, the single crystals suitable for X-ray diffraction analysis were available (SI, Figures S28 and S29). Solved and refined structures^29−32^ were visualized in Mercury.^33^ Both molecules show similar crystal patterns; aromatic regions are arranged in parallel, while the urea groups form hydrogen bonds with the sulfonamide oxygens of the next layer of molecules. In addition, no solvent molecules participate in the lattice and the crystals contain large hydrophobic areas of the alkyl chains (Figure 1, blue). This is in contrast to the previously prepared analogous molecules in which the alkyl group on sulfonamide was the methyl group;^34^ these substances possess regions between the aromatic layers filled with DMSO solvent molecules.

### Solution State Study

In the solution state, the hydrophobic interactions within **6** and **7** also play an important role. The presence of alkyl groups in the receptor moieties enhances their solubility in chloroform. Moreover, it causes extensive receptor aggregation in nonpolar and even in polar solvents. A better insight into the aggregation issues was obtained by studying the behavior of compound **7** in (a) dynamic light scattering (DLS) and (b) dilution experiments. DLS^35^ experiments in chloroform at 25 °C showed that at concentrations exceeding 2 mM, aggregates with a size of about 2000 nm and a 0.2–0.3 polydispersity index are present (SI, Table S1, Figure S30). Below this concentration, the size of the aggregates gradually decreases. Moreover, the addition of H_2_PO_4_^–^ results in a dramatic change in the particle size. In the dilution experiments, the behavior of **7** in the concentration range of 0.6 to 25.6 mM was monitored by ^1^H NMR. The N*H* signals showed significant upfield shifts with decreasing concentration, which allowed monitoring of the aggregate disintegration (SI, Figures S31 and S32). The obtained data were fitted to both the cooperative equal *K* (CoEK) and dimerization/equal *K* (EK)^36,37^ models to elucidate the corresponding self-association constants (SI, Table S2). The self-association constants for **7** obtained from both models show values on the order of hundreds for chloroform. Even in the hydrogen bonding competitive DMSO, the aggregates are formed, with *K* on the order of tens. The associated errors obtained by fitting the data to the CoEK and EK models exhibit that these systems better fit the EK model.

The selectivity and effectivity of the receptor moieties **6** and **7** were evaluated based on the estimation of their apparent association constants with a variety of tetrabutylammonium (TBA^+^) salts of anions (SI, Figures S34–S43).^38−40^ The methodology of ^1^H NMR titration was used, carefully balancing the requirements for estimation of *K*_Ass_ with acceptable accuracy and, when possible, at the low amount of aggregates (*c* below 2 mM, DMSO). These experiments revealed the high selectivity of **6** and **7** toward dihydrogen phosphate and benzoate over halides and hydrogen sulfate, while hexafluorophosphate and perchlorate were not bound at all (SI, Figure S33). Increasing the length of the alkyl chain attached to the receptor moiety appeared to slightly enhance the receptor solubility, and because of the presence of trifluoromethyl groups, the binding abilities of **7** were superior to those of over **6** (Table 1).

**Table 1:** Apparent Association Constants *K*_Ass_^a^ of **6** and **7** Measured by ^1^H NMR Titration in DMSO-*d*_*6*_ with TBA^+^X^–^ at 25 °C

anion/receptor	**6** *K*_Ass_ (M^–1^)	**7** *K*_Ass_ (M^–1^)
H_2_PO_4_^–^	5 200	6 580
BzO^–^	3 860	4 100
Cl^–^	60	90
HSO_4_^–^	11	20
Br^–^	7.5	8.5
PF_6_^–^	n.b.	n.b.
ClO_4_^–^	n.b.	n.b.

a Error, when estimated, was <5%; n.b. = no binding.

### Extraction Experiments

Encouraged by these results, we decided to anchor **7** to the dendritic core to obtain **8**. The anion-extracting ability of the free molecule **7** and its quadruple manifestation on the periphery of a carbosilane dendrimer **8** was thoroughly tested in extraction experiments, by considering the effects such as the repeatability, time dependence, concentration of selected anion in the aqueous phase, and pH dependence (SI, Figures S45–S47, Tables S3–S5). The amounts of extracted anions were obtained by analysis of the organic layer by ^1^H NMR, integrating the signals of tetrabutylammonium. This nondirect approach was justified on the analysis of tetrabutylammonium benzoate extracts, where also the signals of BzO^–^ are ^1^H NMR observable. Moreover, selected experiments were repeated using direct detection of anions in the water phase by Raman spectroscopy.^41^

For compound **7**, the effect of the concentration on the extraction ability was determined using a 5 mM solution of the receptor in chloroform-*d* and water solutions of tetrabutylammonium dihydrogen phosphate with a gradually increasing concentration (5–100 mM).

**Figure 2:**
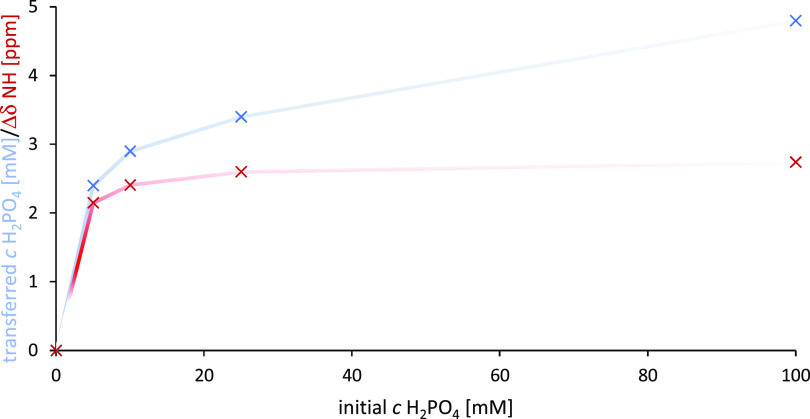
H_2_PO_4_^–^ concentration-dependent extraction efficiency of 5 mM **7**; blue: concentration of H_2_PO_4_^–^ in CDCl_3_ [mM], red: Δδ NH [ppm].

The changes in the dihydrogen phosphate concentration in the chloroform layer (Figure 2, blue) reveal the maximum extraction efficiency with a 5 mM solution of **7** at about 10 mM salt concentration. Further increases in the dihydrogen phosphate concentration result in only a moderate effect on further anion transport to the organic layer. This observation can be clarified by monitoring the NH chemical shift of **7** (Figure 2, red). The complexation of dihydrogen phosphate from its 10 mM aqueous solution corresponds to the full receptor binding ability (considering the partition between the organic and aqueous phases). The further addition of the salt does not cause any increase in the NH chemical shift and thus in complexation. Moreover, such a concentration level in both phases enables the accurate determination of the salt concentration. The time-dependent extraction efficiency was evaluated in the time range of 1 min to 24 h. In this experiment, the immediate partition of the salt without any measurable change with an increasing extraction duration was observed. According to these results, the experimental setup was established: the 10 mM aqueous solutions of TBA^+^ salts were extracted by 5 mM **7** in CDCl_3_ for 15 min, centrifuged for 5 min, and the amount of the salts in the wet organic layers was immediately determined by ^1^H NMR, integrating the signal of tetrabutylammonium cation (Tables S6 and S7).

**Figure 3:**
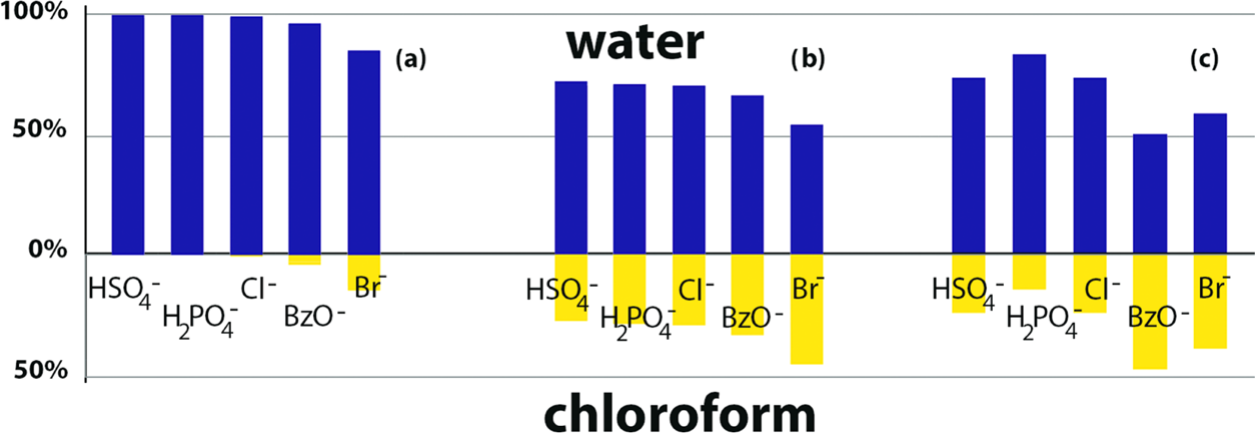
Results of extraction experiments. Concentration of anions in aqueous and organic phase after extraction from 10 mM aqueous solution: (a) natural partition, (b) 5 mM **7** in chloroform layer, and (c) 1.25 mM **8** in chloroform layer. The amount of extracted HSO_4_^–^ corresponds to its content in the formed precipitate.

The obtained results were compared to those obtained for the particular anion extraction into CDCl_3_ without any added **7**, at the same conditions (Figure 3a). In the absence of the receptor, only 15% of bromide, 4% of benzoate, and 0.8% of chloride are extracted, while hydrogen sulfate and dihydrogen phosphate are apparently not extracted. The presence of **7** in the chloroform layer enhances the anion extraction ability, but to a similar extent for all anions (Figure 3b). If the 5 mM **7** in the chloroform layer is replaced with approximately the same amount of anchored binding units in receptor **8**, the interaction becomes rather more specific, and the amounts of extracted anions considerably differ. In the case of hydrogen sulfate, a precipitate is readily formed on the surface of the chloroform layer. According to the integration of dendrimer signals, shaking with 10 mM aqueous HSO_4_^–^ causes the precipitation of 60% of **8**. The precipitate was filtered off, dissolved completely in DMSO-*d*_6_, and analyzed. The amount of extracted HSO_4_^–^ in Figure 3c corresponds to its content in the precipitate. Regarding dihydrogen phosphate, 20% precipitation of the complex occurs and the extraction ability of **8** is somewhat lower than that of **7**. This applies also to chloride anions. On the other hand, bromide and benzoate do not cause the receptor precipitation, and in the case of benzoate, the anion is extracted in a higher concentration than with free receptor moiety **7** (4.9 mM of H_2_PO_4_^–^ transferred by **8** compared to 3.4 mM with the use of **7**). Moreover, the shifts of receptor NH groups correspond to the state where the binding sites are fully occupied by benzoate (SI, Figure S49, Table S7). We also investigated the possibility of separation of benzoate ions from their mixtures with chlorides or bromides. As the cations are the same for both benzoate and halides, we performed the readout of the extracted anion concentration from the aromatic region directly integrating the signals of the benzoate anion. We obtained the same result for benzoate extraction in the presence or absence of tetrabutylammonium bromide, confirming the receptor selectivity. In the case of chlorides, the efficiency seems to be slightly decreased. The broadening of the aromatic signal of benzoate under these conditions affects the accuracy of the determination of anion concentration in the organic layer by simple integration. For this reason, we decided to employ Raman spectroscopy as a complementary method to monitor the anion transfer (SI, Figures S50 and S51 Tables S10 and S11). Advantageously, this technique offers the opportunity to monitor directly the changes in the composition of the anion mixture in the aqueous phase. Moreover, the presence of Raman-active tetrabutylammonium counterions allows the indirect determination of the chloride concentration. With this technique, higher-concentration solutions are required compared to those used for ^1^H NMR, and this was achieved by performing the anion extraction at a larger scale (10 times higher) and concentrating the postextraction aqueous phase to the level required to the Raman spectroscopy. The procedure is described in detail in the SI Experimental methods. It has been proven that from the aqueous solution containing a mixture of 10.0 mM TBA^+^BzO^–^ and 10.0 mM TBA^+^Cl^–^, about 3.2 mM benzoate was transferred into chloroform. In addition, 1.2 mM chlorides was co-extracted. Those results confirm the high selectivity of **8** toward benzoate, even in the presence of halides.

**Figure 4:**
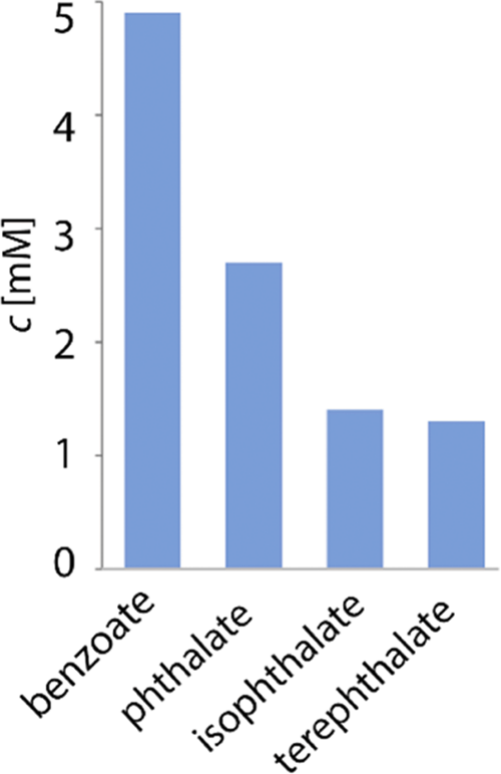
Comparison of extraction of 10 mM aqueous phthalates and benzoate to CDCl_3_ containing 1.25 mM **8**.

The affinity of **8** to benzoate inspired us to perform experiments comprising the extraction of phthalates that are important industrial pollutants of water bodies.^42^ Unfortunately, the second charge in the dicarboxylate molecule causes 30% dendrimer precipitation and significantly lowers the extraction ability of **8** (Figure 4, Table S8).

After anion extraction, receptor **8** can be recycled by re-extraction of the chloroform layer with deionized water. Except for benzoate, all of the anions can be transferred back to the aqueous environment, leaving the free **8** in the chloroform layer (Figure S48). Benzoate stays in the complex even after three cycles of re-extraction and must be separated from the complex by nanofiltration of the mixture with methanol washing.^43^

### Computational Studies

To gain some insight into the behavior of the anions at the water–chloroform interface in the presence of **7** and **8**, we performed a series of molecular dynamic simulations^44^ in three different conditions: (1) using dry chloroform as a solvent, (2) using chloroform with only a few water molecules (wet chloroform), and (3) using chloroform and enough water to create an extensive interface between the two phases. We run these simulations for chloride, dihydrogen phosphate, and benzoate, all with tetrabutylammonium as a counterion. In the case of **7**, the accumulation of the receptor molecules at the water–chloroform interface was observed. Further examination showed the possible formation of water droplets containing the ions surrounded by receptor **7** in the chloroform bulk. This is in accordance with the observed results: a similar amount of anion is extracted regardless of the anion type because the receptor can bring small droplets of water to the organic layer. These conditions reduce the selectivity of the receptor, as no specific contact between the anions and the receptor itself is made (SI, Figures S52 and S53).

**Figure 5:**
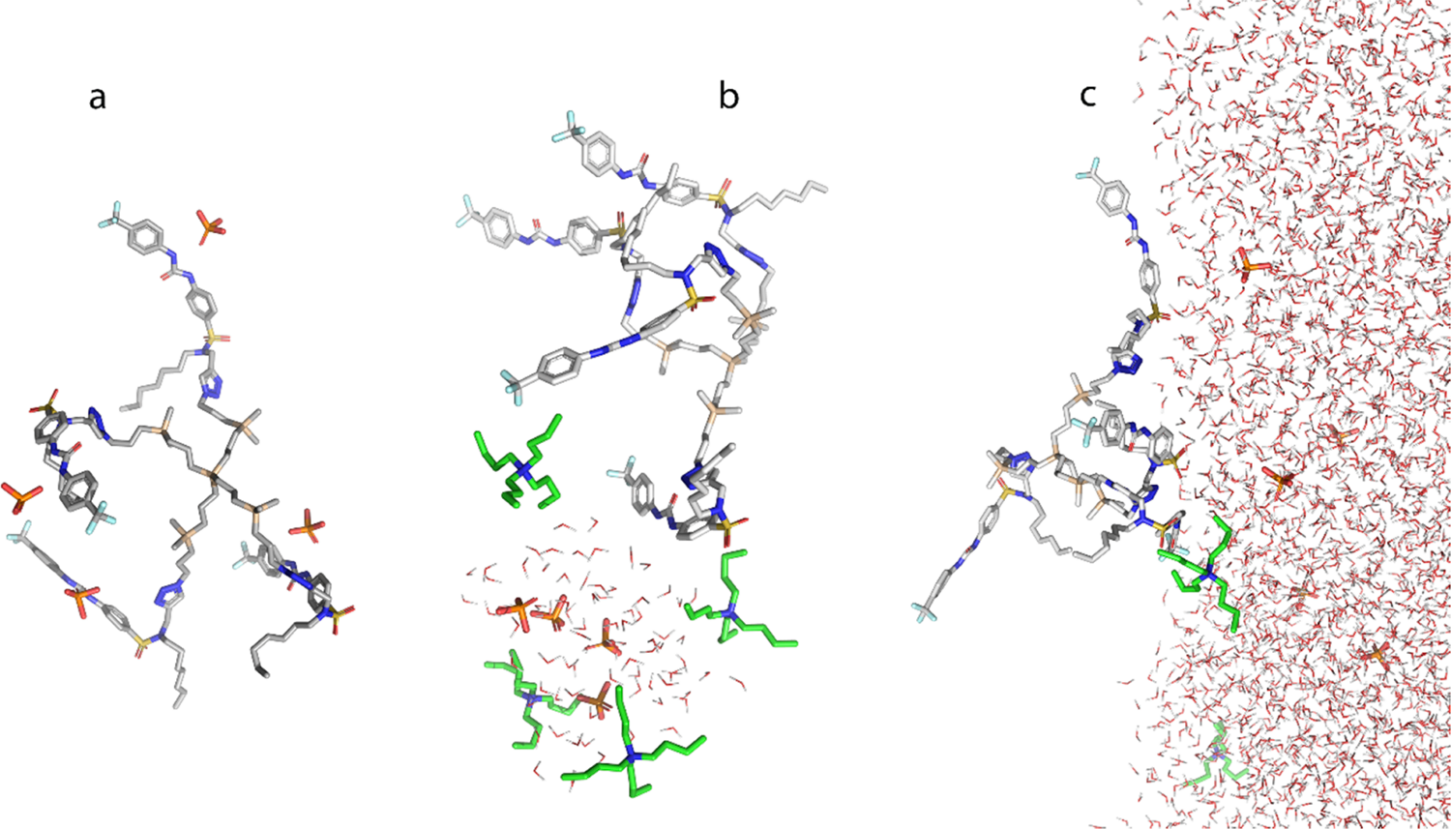
Simulation of behavior of **8** in the presence of TBA^+^ H_2_PO_4_^–^: (a) dry chloroform, (b) wet chloroform, and (c) water–chloroform interface.

In the case of **8**, a similar behavior can be observed with dihydrogen phosphate (Figure 5) and chloride anions (SI, Figure S54). In dry chloroform, **8** forms a stable complex with four dihydrogen phosphate anions (Figure 5a). The presence of even a small amount of water makes this complex unstable, and a very fast formation of water droplets containing TBA^+^ H_2_PO_4_^–^ surrounded by **8** (Figure 5b) is observed. The addition of more water molecules causes the formation of two clear phases, with the ions distributed in the polar phase, and no complex between **8** and dihydrogen phosphate can be traced (Figure 5c), even if the receptor can place itself at the solvent interface.

**Figure 6:**
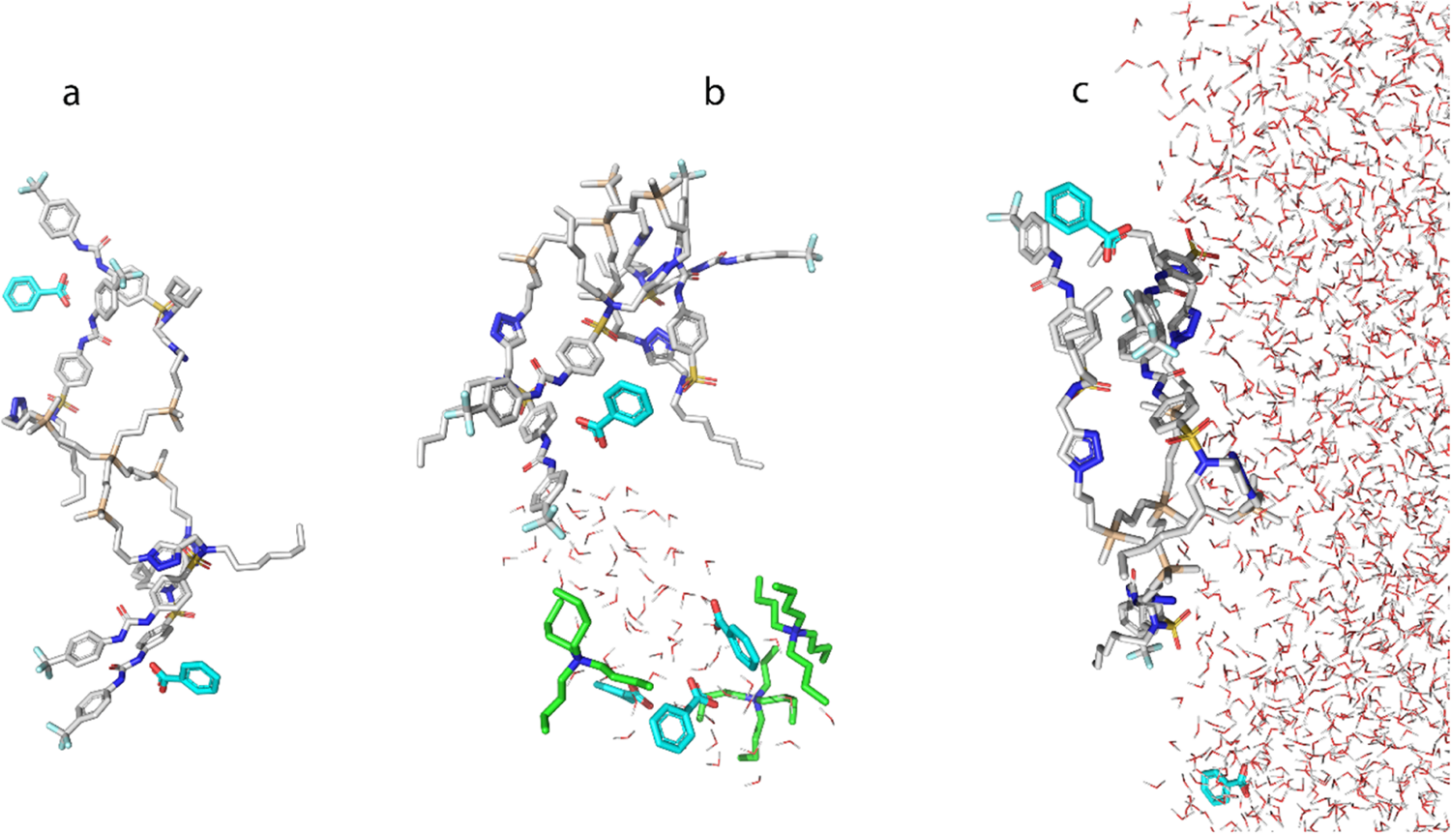
Simulation of behavior of **8** in the presence of TBA^+^ BzO^–^ (a) dry chloroform, (b) wet chloroform, and (c) water–chloroform interface.

The molecular dynamics simulations with the benzoate anion produced a significantly different set of results. The simulation with **8** and the benzoate anion in dry chloroform (Figure 6a) shows the hydrogen bonding between the carboxylic functions with the urea groups. The interaction of one benzoate anion with the two arms of **8** is also observed. The interaction between **8** and the anion is further visible in the wet chloroform, with the ion placed outside the water droplet (Figure 6b). Finally, the complex between the benzoate and **8** is observed even in the presence of a large amount of water (Figure 6c), supporting the experimental observation that **8** is selective for benzoate under the conditions of the liquid–liquid extraction.

## Conclusions

Amphiphiles are formed by adding lipophilic chains to the structure of receptors containing urea binding sites in conjugation with sulfonamide groups. Despite aggregation in a nonpolar environment, these molecules proved to be highly selective receptors of dihydrogen phosphate and benzoate. The main purpose of these novel receptor compounds is their use in liquid–liquid extraction. The amphiphilic nature of these molecules leads to their accumulation at the water–organic interface, facilitating the transport of anions into the organic phase. Although even oxo-anions can be transported this way, the selectivity of the receptor is lost as whole water droplets surrounded by the amphiphile could be transferred into chloroform. These droplets can incorporate ions of different natures, and such transport does not require any specific ion/receptor interaction. On the other hand, when these receptor moieties are anchored to a dendritic core, their ability for specific benzoate-ion extraction is observed. The dendritic structure might provide a hydrophobic microenvironment suitable for hydrogen bonding with some specific anions. When these ions are not surrounded by a high amount of water in their solvation shell, the complex remains relatively stable even in the presence of water, as observed in the case of benzoate. The findings of the present work not only present a selective extraction agent for benzoate but also elucidate some physicochemical principles of anion extraction into the organic phase using amphiphiles, also taking into consideration the role of water molecules at the interface.

## Supporting Information Available

Detailed description of experimental methods, synthesis, characterization of compounds, and the raw data used to calculate aggregation or association constants, extraction data, X-ray analyses, and figures obtained from computational analysis (PDF)
Crystallographic data 1 (CIF)
Crystallographic data 2 (CIF)

## Supplementary Material

ao3c06767_si_001.pdf

ao3c06767_si_002.cif

ao3c06767_si_003.cif
