# Supplementary material for: An Insight into Anion Extraction by Amphiphiles: Hydrophobic Microenvironments as a Requirement for the Extractant Selectivity
Source: ACS Omega. 2023 Nov 3;8(46):44221–8. doi: 10.1021/acsomega.3c06767 (PMC10666219; doi:10.1021/acsomega.3c06767)
Supplement: Supplementary file 1 — ao3c06767_si_001.pdf [file ao3c06767_si_001.pdf]

An Insight into Anion Extraction by Amphiphiles: Hydrophobic Microenvironments as a Requirement for the Extractant Selectivity

Karolína Salvadori[1,2], Alessia Onali[3], Gregory Mathez[3], Václav Eigner[4], Marcela Dendisová[1], Pavel Matějka[1], Monika Mullerová[2], Andrea Brancale[3], Petra Cuřínová[3]*

1) Department of Physical Chemistry, University of Chemistry and Technology Prague, Technická 5, Prague 6, 16628, Czech Republic

2) Department of Bioorganic Chemistry and Biomaterials, Institute of Chemical process Fundamentals of the CAS, v.v.i., Rozvojová 135, Prague 6, 16502, Czech Republic

3) Department of Organic Chemistry, University of Chemistry and Technology Prague, Technická 5, Prague 6, 16628, Czech Republic

4) Department of Solid-State Chemistry, University of Chemistry and Technology Prague, Technická 5, Prague 6, 16628, Czech Republic

Contents

1 Experimental methods S3

2 Synthesis S8

3 Spectral characterization of prepared compounds S13

4 X-Ray single crystal diffraction analysis S30

5 Dynamic light scattering S32

6 Titration experiments S33

Dilution experiments S33

Complexation study S36

8 Extraction S47

Time dependence S47

Concentration dependence S48

pH dependence S49

Extraction of different anions S50

Anion mixtures S52

9 Computational studies S56

10 References S59

1 Experimental methods

General: All chemicals were purchased from commercial sources and were used without further purification. Analytical TLC was carried out on foil sheets coated with silica gel containing a fluorescent indicator - 60 F_{254} (Merck) or on TLC plates covered by aluminium oxide with fluorescent indicator 60 F_{254} neutral. The analyte was detected by UV light (wavelength 254 nm). Preparative TLC chromatography was carried out on 20 × 20 cm glass plates covered by silica gel 60 PF_{254} (Merck).

NMR: ^{1}H (400.1 MHz), ^{13}C (100.6 MHz), ^{19}F (376.5 MHz), and ^{29}Si (79.5 MHz) NMR spectra were recorded using a Bruker Avance 400 spectrometer (Bruker Biospin, Rheinstetten, Germany) at 25 °C. Chloroform was stabilized with silver foil and both used solvents ($CDCl_3$ and DMSO-d_6) were stored over molecular sieves. The ^{1}H and ^{13}C NMR spectra were referenced to the line of the solvent (δ/ppm; δ_H/δ_C: DMSO-d_6, 2.50/39.52; δ_H: $CDCl_3$ 7.26 ppm). The ^{19}F spectra were referenced to the line of standard hexafluorobenzene (δ_F/ppm; −163.00), the ^{29}Si spectra were referenced to the line of external standard hexamethyldisilane (δ_{Si}/ppm; −19.79).

HRMS: analyses were performed at MicrOtof III spectrometer (Bruker Daltonik, Bremen, Germany) with ESI or APCI ionisation in positive mode. APCI settings: capillary 4000 V, end plate offset -500V, corona needle 3000 nA, source temperature 400°C, dry gas: N_2 at 350°C/4 L/min, nebulizer N_2, 1.5 Bar, m/z 80-1500, calibration at low conc. Tuning Mix ESI/APCI (Agilent). ESI: capillary 4000 V, end plate offset -500V, source temperature 120°C, dry gas: N_2 4 L/min, nebulizer N_2, 1.5 Bar, m/z 80-4000, calibration at Na formate clusters.

FTIR: analysis was performed on a Nicolet 6700 spectrometer (Thermo-Nicolet, USA) connected with a GladiATR diamond ATR adapter (PIKE, USA), reflectance setup, DTGS KBr detector, with the following parameters: spectral range: 4000 - 400 cm^{-1}, resolution: 4 cm^{-1},

number of spectra accumulations: 64, apodization: Happ-Genzel. The spectra were processed by Omnic 9 (Thermo-Nicolet Instruments Co., USA) with baseline correction.

X-ray: The single crystal X-ray diffraction studies of **6** were performed using Rigaku OD Supernova equipped with Atlas S2 CCD detector and Cu-Kα radiation from mirror collimated sealed tube, with the sample cooled using Oxford cryo-systems Cryostream 800. With compound **7**, Bruker D8 Venture equipped with Photon III 7 CMOS detector and Cu-Kα radiation from Incoatec microfocus sealed tube was used, with the sample cooled using Oxford cryo-systems DTC. The data reduction, scaling, and absorption correction of **6** was handled in CrysAlis PRO[S1] and **7** in Apex4.[S2] The phase problem was solved using charge flipping methods in Superflip.[S3] The structures were refined using Crystals.[S4] Hydrogen atoms were refined using a mixture of restrained refinement for hydrogen atoms attached to non-carbon atoms and riding constraints for hydrogen atoms attached to carbon atoms. The MCE was used for visualization of residual electron density maps,[S5] Damond 3[S6] and Mercury[S7] were used for molecular graphics.

Organic solvent nanofiltration was performed using solvent-resistant stirred cell (Millipore) equipped with 3 kDa MWCO regenerated cellulose ultrafiltration discs (Ultracel, Millipore), and PTFE encapsulated O-rings (Teflex, Eriks), with nitrogen as a driving gas. The material was dissolved in a mixture of MeOH and DCM in 1:3 ratio and passed through the membrane (5 mbar trans-membrane pressure) until the reduction factor of 10 was reached. The retentate was then diluted to the original volume by the same solvent mixture and the cycle was repeated as necessary. ^{1}H NMR was used to monitor the purification progress: 4-5 cycles were typically sufficient to obtain an analytically pure product.[S8]

Computational studies: All molecular modelling experiments were performed on a custom-made machine with Intel i9-12900K x 24, NVIDIA RTX A5000, running Ubuntu 22.04.

Molecular Operating Environment (MOE) 2019.10[S9] and the Schrödinger suite (release 2021-2)[S10] were used as the main molecular modelling software packages. The single receptors and the dendrimer molecules were built in MOE, and energy was minimised using the OPLS4 force field in Maestro. Molecular dynamics simulations were performed using Desmond. Three simulation setups were created, each with varying amounts of water present in the system. The first simulation setup was designed to study the system in the absence of water, using chloroform as a sole solvent. The simulation box had an 87 Å cubic shape, and a density of 1.5 g/cm^3. The second simulation setup was used to investigate the system in a chloroform environment with a small amount of water (100 water molecules and 4907 chloroform molecules in total, in an 87 Å cubic simulation box). The third setup was used to simulate the system in a chloroform environment with an order of magnitude larger amount of water (4400 water molecules and 4907 chloroform molecules), in a 95 Å cubic simulation box. The OPLS4 force field and TIP3P water model were used in all the experiments. Four individual receptors, or a single dendrimer, were added to the simulation boxes, each in the presence of 4 of the tetrabutylammonium salts of the appropriate anion (chloride, dihydrogen phosphate; benzoate). All simulations were run for 1000 ns, with a 2 fs time step, in the NPT ensemble, with constant temperature (300 K) and pressure (1 atm). All other parameters were set using the Desmond default values. Images were created using PyMOL.

Dynamic light scattering*:* The size of aggregates was examined by dynamic light scattering nanoparticle size analyser NanoBrook Omni, sold by Brookhaven Instruments Corporation (USA, NY) using a 90° scattering angle. The measurements were performed in chloroform in a 1-cm optical glass cuvette (BI-SCGO) at 25 °C. The environment temperature was actively controlled inside the NanoBrook. The used concentration range started from 25 mM and was gradually reduced to 1 mM concentration. Before each measurement, the solution was 15 minutes sonicated. To ensure the consistency of reported results, all measurements were

performed five-time. Experimental data were processed by using the software Particle Solutions v 3.4.

Dilution experiments were performed in two different solvents ($CDCl_3$ and DMSO-d_6) in the concentration range from 0.5 to 25 mM. In both solvents, urea N*H* signals revealed significant downfield shifts with increasing concentration. The ^{1}H NMR titration experiments were performed in DMSO-d_6 at concentrations specified for individual measurements in the part Titration experiments of this SI. The complexation studies were performed at a constant concentration of receptors to avoid potential problems with chemical shift changes caused by dilution. To the solution of receptors were gradually added aliquots of solutions of anions in the form of their TBA^+ salts.

Association constants and self-association constants were calculated using the freely available program Bindfit. ^{1}H NMR dilution studies were fitted to both cooperative equal *K*(COEK) and dimerization/equal *K*(EK) models. All the data relating to the calculation of those constants can be accessed online, through the links given for each association event.

Liquid-liquid extraction experiments were performed with receptor **7** (1000 μL, 5 mM chloroform-d solution) or **8** (1000 μL, 1.25 mM chloroform-*d* solution), respectively, layered over with corresponding TBA^+ salt solution in ultrapure water (1000 μL, 10 mM). The layers were mixed by intensive shaking for 15 minutes, then the layers were separated by centrifugation. The NMR spectrum of the wet chloroform layer was recorded. Obtained spectra were processed by phase and baseline correction (polynomial fit). Integral values of the receptor proton signals were compared with the signal of residual $CHCl_3$, and the newly appearing signals of TBA^+. Those integrals were used to calculate the extracted concentration of anion and to evaluate the precipitation of dendrimer **8**. Representative spectral parts used for the generation of data for Tables S3-S9 are shown in Figures S45-47.

The extraction of phosphates was also monitored focusing on repeatability, time dependence, anion concentration, and pH dependence. In the case of pH dependence measurements, 10 mL of chloroform-*d* solution containing 5 mM of receptor **7** was shaken for 15 minutes with 10 mL of aqueous solution of corresponding pH (Table S5). Then the layers were separated by centrifugation. Besides the organic phase, also the aqueous phase was analysed by using WTW Lab pH Meter inoLab Multi 9620 IDS equipped with IDS Precision pH electrode SenTix 980.

Raman spectroscopy: To study the mixture of anions the Raman spectra of the aqueous phase were collected using an Equinox 55/s FT-NIR spectrometer (Bruker, Germany) with an FRA 106/s FT Raman module (Bruker, Germany). The irradiation source was a Nd:YAG laser (Coherent, USA) with 1064-nm excitation. The FT-Raman spectrometer was equipped with a Ge detector cooled with liquid nitrogen. Each spectrum was collected with the following parameters: 2048 scans at 4 cm^{-1} resolution and 400 mW laser power for one spectrum; the final noise-reduced spectrum was an average of 15 accumulations. Aqueous solutions of TBABzO and TBACl and their mixtures were used for calibration. Due to the low sensitivity of method, the extractions were performed in 10 mL scale: 10 mL of 10.0 mM TBABzO and 10.0 mM TBACl in water was extracted with 10 mL of 1.25 mM chloroform solution of **8**. After extraction, the water phase was separated and lyophilized. The residue was dissolved of 1 mL of ultrapure water (thus obtaining 10 times more concentrated solution) and analysed. The spectra were uploaded into TQ Analyst and were compared to the spectrum of an aqueous phase containing the mixture of 100 mM TBABzO and 100 mM TBACl before extraction (Figure 50).

2 Synthesis

In comparison to our previously published synthetic approaches,[S11] the propargyl motif was introduced into the structure of urea receptors already in the first reaction step. It turned out that propargylation during advanced stages of synthesis can be problematic. On the contrary, the alkylation of sulfonamidic hydrogen by aliphatic alkyl bromides proceeds without any complications.

4-nitro-*N*-(prop-2-yn-1-yl)benzenesulfonamide 1

To the solution of propargylamine (250 μL, 3.9 mmol) in pyridine (50 mL) 1 g of *p*-nosyl chloride (4.5 mmol) was added. The reaction was stirred for 1 day at ambient temperature and the progress was monitored by TLC. Upon completion of the reaction the mixture was poured into aqueous HCl (to reach a pH value of ca. 2) and the product was extracted into ethyl acetate (3 × 50 mL). The combined organic layers were dried over $MgSO_4$ and filtered. The filtrate was evaporated to give a title compound **1** as a yellow solid (0.89 g, 95% yield). **^{1}H NMR (400 MHz, DMSO-d_6)** δ: 8.54 (t, 1H, N*H*, J = 6.0 Hz); 8.42 (d, 2H, Ar*H*, J = 9.4 Hz); 8.07 (d, 2H, Ar*H*, J = 9.0 Hz); 3.79 (dd, 2H, CH_2, J_1 = 5.9 Hz, J_2 = 2.5 Hz); 3.02 (t, 1H, CH, J = 2.5 Hz) ppm. In accordance with literature.[S12]

***N*-alkyl-*p*-nitrosulfonamides 2 and 3**

In a round-bottomed 100 mL flask 4-nitro-*N*-(prop-2-yn-1-yl)benzenesulfonamide **1** (420 mg, 1.75 mmol) was suspended in water (50 mL). To this suspension, the aqueous solution of NaOH (1 eq., 70 mg in 5 mL) was poured and the mixture was sonicated for 5 min. Then, the aqueous solution was filtrated. Filtrate was frozen by immersing the flask in a bath of dry ice and was lyophilized to dryness. In this step total conversion was assumed and the sodium salt was used without further purification.

To the sodium salt of **1** (obtained in the previous step) was added acetonitrile (50 mL), and the mixture was stirred at the ambient temperature to dissolve the solid. Then, 1.5 eq. of corresponding alkyl bromide was added, and the stirring continued at the ambient temperature for 2 days. The reaction progress was monitored by TLC. Upon completion of the reaction, the solvent was removed under reduced pressure. The crude reaction mixture was dissolved in ethyl acetate (50 mL) and the organic layer was washed with a saturated solution of $NaHCO_3$ (3 x 50 mL). Then, the organic phase was dried over $MgSO_4$, filtered, and evaporated to give both compounds as yellowish oil.

***N*-butyl-4-nitro-*N*-(prop-2-yn-1-yl)benzenesulfonamide 2:** ^{1}H NMR (400 MHz, DMSO-d_6) δ: 8.40 (d, 2H, Ar*H*, J = 8.9 Hz); 8.10 (d, 2H, Ar*H*, J = 8.9 Hz); 4.16 (d, 2H, CH_2, J = 2.3 Hz); 3.18 (t, 2H, CH_2, J = 7.2 Hz); 3,06 (t, 1H, CH, J = 2.4 Hz); 1.52 (m, 2H, CH_2); 1.34–1.23 (m, 2H, CH_2); 0.88 (t, 3H, CH_3, J = 7.4 Hz) ppm. ^{13}C NMR (101 MHz, DMSO-d_6) δ: 149.9; 144.0; 129.0; 124.4; 76.61; 76.55; 46.3; 36.2; 29.0; 19.1; 13.4 ppm. IR 3227; 3108; 3083; 3071; 3041; 2958; 2931; 2872; 2117; 1610; 1527; 1346; 1303; 1159 cm^{-1}. HRMS calcld $[C_{13}H_{16}N_2O_4S+H]^+$ 297.0903 found *m*/*z* 297.0907 $[M+H]^+$ The title compound **2** was obtained as yellow oil in 80 % yield.

***N*-heptyl-4-nitro-N-(prop-2-yn-1-yl)benzenesulfonamide 3:** ^{1}H NMR (400 MHz, DMSO-d_6) δ: 8.41 (d, 2H, Ar*H*, J = 6.9 Hz); 8.10 (d, 2H, Ar*H*, J = 6.9 Hz); 4.16 (d, 2H, CH_2, J = 2.4 Hz); 3.17 (t, 2H, CH_2, J = 7.1 Hz); 3.08 (t, 1H, CH, J = 2.3 Hz); 1.52 (q, 2H, CH_2); 1.30 – 1.19 (m, 8H); 0.85 (t, 3H, CH_3, J = 6.6 Hz) ppm. ^{13}C NMR (101 MHz, DMSO-d_6) δ: 149.9; 144.0; 129.0; 124.4; 76.66; 76.54; 46.6; 36.2; 31.2; 28.1; 26.9; 25.9; 22.0; 13.9 ppm. IR 3287; 3105; 2954; 2929; 2871; 2858; 1719; 1606; 1592; 1529; 1347; 1310; 1161 cm^{-1}. HRMS calcld $[C_{16}H_{23}N_2O_4S]^+$ 339.1373; found *m*/*z* 339.1370 $[M+H]^+$; calcld $[C_{16}H_{22}N_2O_4SNa]^+$ 361.1192; found *m*/*z* 361.1191 $[M+Na]^+$. The title compound **3** was obtained as yellow oil in 85 % yield.

***N*-alkyl-*p*-aminosulfonamides 4 and 5**

N-alkyl-*p*-nitrosulfonamide **2** or **3** (1.2 mmol) was dissolved in ethanol (50 mL). Then, the reaction mixture was heated to reflux and 15 eq. of tin(II) chloride dihydrate (18 mmol) were added. The mixture was stirred for 6 h, and the progress was monitored by TLC. Upon completion of the reaction, the mixture was cooled to room temperature. The solvent was removed under reduced pressure, and the crude reaction mixture was dissolved in ethylacetate (50 mL). The organic layer was firstly washed with aqueous potassium hydroxide (5 M, 100 mL) then, with a saturated solution of $NaHCO_3$ (3 x 50 mL). Finally, the organic phase was dried over $MgSO_4$, filtered, and evaporated to give both title compounds as yellow oil in quantitative yield.

4-amino-*N*-butyl-*N*-(prop-2-yn-1-yl)benzenesulfonamide 4: ^{1}H NMR (400 MHz, DMSO-*d*$_6$) δ: 7.40 (d, 2H, Ar*H*, *J* = 8.7 Hz); 6.60 (d, 2H, Ar*H*, *J* = 8.8 Hz); 6.00 (s, 2H, NH_2); 3.99 (d, 2H, CH_2, *J* = 2.4 Hz); 3,08 (t, 1H, CH, *J* = 2.4 Hz); 3.02 (t, 2H, CH_2, *J* = 7.2 Hz); 1.45 (m, 2H, CH_2); 1.26 (m, 2H, CH_2); 0.86 (t, 3H, CH_3, *J* = 7.3 Hz) ppm. ^{13}C NMR (101 MHz, DMSO-*d*$_6$) δ: 153.0; 129.3; 123.1; 112.6; 77.9; 75.8; 45.8; 36.0; 29.0; 19.2; 13.5 ppm. IR 3472; 3377; 3282; 3065; 3040; 2959; 2932; 2871; 2114; 1627; 1595; 1503; 1338; 1313; 1146 cm^{-1}. HRMS calcld $[C_{13}H_{18}N_2O_2S]^+$ 267.1162 found *m/z* 267.1161 $[M]^+$

4-amino-*N*-heptyl-N-(prop-2-yn-1-yl)benzenesulfonamide 5: ^{1}H NMR (400 MHz,DMSO-*d*$_6$) δ: 7.39 (d, 2H, Ar*H*, *J* = 8.7 Hz); 6.59 (d, 2H, Ar*H*, *J* = 8.8 Hz); 6.00 (s, 2H, NH_2); 3.99 (d, 2H, CH_2, *J* = 2.4 Hz); 3.09 (t, 1H, CH, *J* = 2.4 Hz); 3.00 (t, 2H, CH_2, *J* = 7.2 Hz); 1.46 (q, 2H, CH_2, *J* = 7.4 Hz); 1.28 – 1.19 (m, 8H); 0.85 (t, 3H, CH_3, *J* = 6.7 Hz) ppm. ^{13}C NMR (101 MHz, DMSO-*d*$_6$) δ: 153.0; 129.2; 123.1; 112.6; 77.9; 75.8; 46.1; 36.0; 31.2; 28.2; 26.9; 26.0; 22.0; 13.9 ppm. IR 3476; 3379; 3286; 2954; 2927; 2869; 2856; 1627; 1595; 1503; 1314; 1147 cm^{-1}. HRMS calcld $[C_{16}H_{25}N_2O_2S]^+$ 309.1631; found *m/z* 309.1631 $[M+H]^+$; calcld $[C_{16}H_{24}N_2O_2SNa]^+$ 331.1450; found m/z 331.1448 $[M+Na]^+$.

Ureido receptors 6 and 7

To a stirred solution of *N*-alkyl-*p*-aminosulfonamides **4** or **5** (0.4 mmol) in dichloromethane, 1.2 eq. of corresponding aromatic isocyanate was added dropwise. The reaction mixture was stirred overnight and then poured into water. The product was precipitated and extracted into chloroform. The organic phase was dried over $MgSO_4$, filtered and the filtrate was evaporated. Ureido receptor **6** was cleaned by crystallization from DMSO or by using preparative alumina TLC (EtOAc:PE 1:4). Ureido receptor **7** was purified by using preparative silicagel TLC (EtOAc:PE 1:4).

***N*-butyl-4-(3-phenylureido)-*N*-(prop-2-yn-1-yl)benzenesulfonamide 6:** ^{1}H NMR (400 MHz, DMSO-d_6) δ: 9.25 (s, 1H, N*H*); 8.89 (s, 1H, N*H*); 7.72 (d, 2H, Ar*H*, *J* = 8.9 Hz); 7.66 (d, 2H, Ar*H*, *J* = 8.9 Hz); 7.47 (d, 2H, Ar*H*, *J* = 7.8 Hz); 7.29 (m, 2H, Ar*H*); 6.99 (t, 1H, Ar*H*, *J* = 7.3 Hz); 4.08 (d, 2H, CH_2, *J* = 2.2 Hz); 3.12-3.08 (m, 3H, CH+CH_2); 1.49 (m, 2H, CH_2); 1.28 (m, 2H, CH_2); 0.87 (t, 3H, CH_3, *J* = 7.3 Hz) ppm. ^{13}C NMR (101 MHz, DMSO-d_6) δ: 152.2; 144.1; 139.3; 130.5; 128.8; 128.7; 122.3; 118.5; 117.4; 77.4; 76.1; 46.0; 36.1; 29.0; 19.2; 13.5 ppm. IR 3395; 3273; 3138; 3057; 2962; 2930; 2869; 2113; 1723; 1712; 1677; 1591; 1533; 1496; 1441; 1308; 1235; 1203; 1147cm^{-1}. HRMS calcld $[C_{20}H_{23}N_3O_3S]^+$ 386.1533 found *m*/*z* 386.1531 $[M]^+$ The title compound **6** was obtained as crystalizing syrup in 65 % yield.

***N*-heptyl-*N*-(prop-2-yn-1-yl)-4-(3-(4-(trifluoromethyl)phenyl)ureido)benzenesulfonamide 7:** ^{1}H NMR (400 MHz,DMSO-d_6) δ: 9.36 (s, 1H, N*H*); 9.31 (s, 1H, N*H*); 7.77 – 7.63 (m, 8H, Ar*H*); 4.08 (d, 2H, CH_2, *J* =2.4 Hz); 3.13 – 3.06 (m, 3H, CH_2+CH); 1.49 (q, 2H, CH_2, *J* = 7.1 Hz); 1.28 – 1.20 (m, 8H); 0.86 (t, 3H, CH_3, *J* =6.7 Hz) ppm. ^{13}C NMR (101 MHz, DMSO-d_6) δ: 152.0; 143.7; 143.1; 131.0; 128.6; 126.1 (q, *J* = 32.8); 125.9; 123.2 (m); 118.1; 117.7; 77.4; 76.1; 46.3; 36.1; 31.2; 28.2; 26.9; 25.9; 22.0; 13.9 pm. ^{19}F NMR (376 MHz, DMSO-d_6) δ: -60.11 ppm. IR 3395; 3375; 3327; 3277; 2957; 2929; 2873; 2857; 1726; 1593; 1537; 1314; 1149

cm^{-1}. HRMS calcld $[C_{24}H_{28}F_3N_3O_3SNa]^+$ 518.1696; found *m*/*z* 518.1677 $[M+Na]^+$. The title compound **7** was obtained as crystalizing syrup in 60 % yield.

Dendrimer 8

In the 10 mL microwave vial, the azide-terminated dendrimer (0.15 g, 0.2 mmol) with receptor **7** (5 equivalents, 0.5g), copper(I) iodide (0.5 equiv. per azide group) and DIPEA (10 equiv. per azide group) were dissolved in 6 mL of DMF. The vial was sealed and irradiated by microwaves for 1.5 h at 120 °C. After cooling, the contents of the vial were poured into 1M HCl and the mixture was extracted with ethyl acetate (3 × 30 mL). The combined organic layers were dried over $MgSO_4$, filtered and the filtrate was evaporated giving the crude product. The residue was dissolved in DMSO (5 mL) and an excess of chelex100 sodium form (Sigma-Aldrich) was added. The mixture was stirred overnight. The solids were filtered off and the filtrate was purified by OSN (see above). The product was obtained as a brownish syrup in 75% yield.

^{1}H NMR (400 MHz, DMSO-d_6) δ: 9.30 (s, 4H, N*H*); 9.26 (s, 4H, N*H*); 7.91 (s, 4H); 7.79 – 7.45 (m, 32H); 4.35 (s, 8H); 4.24 (t, 8H, J = 6.8 Hz); 3.02 (t, 8H, J = 7.4 Hz); 1.76 –1.69 (m, 8H); 1.32 – 1.05 (m, 48H); 0.78 (t, 12H, J = 7.1 Hz); 0.48 (m, 16H); 0.36 (m, 8H); -0.11 (s, 24) ppm. ^{13}C NMR (101 MHz, DMSO-d_6) δ: 151.9; 143.4; 143.0; 131.5; 128.1; 125.94; 125.91; 124.4 (q, J = 271.0 Hz); 123.8; 122.1 (q, J = 31.8 Hz); 118.0; 117.8; 52.1; 47.5; 42.7; 31.1; 28.1; 27.6; 25.8; 24.6; 21.9; 19.2; 18.0; 16.8; 13.8; 11.4; -3.8 ppm. ^{19}F NMR (376 MHz, DMSO-d_6) δ: -60.11 ppm. ^{29}Si NMR (79 MHz, DMSO-d_6) δ: 1.98 (4Si); 0.68 (Si) ppm. IR 3344; 3297; 3198; 3120; 3066; 2953; 2924; 2870; 2856; 1717; 1592; 1536; 1310; 1153 cm^{-1}. HRMS calcld $[C_{128}H_{184}N_{24}F_{12}O_{12}S_4Si_5]^+$ 2747.6306 found *m*/*z* 2747.6314 $[M+H]^+$, 100%.

3 Spectral characterization of prepared compounds

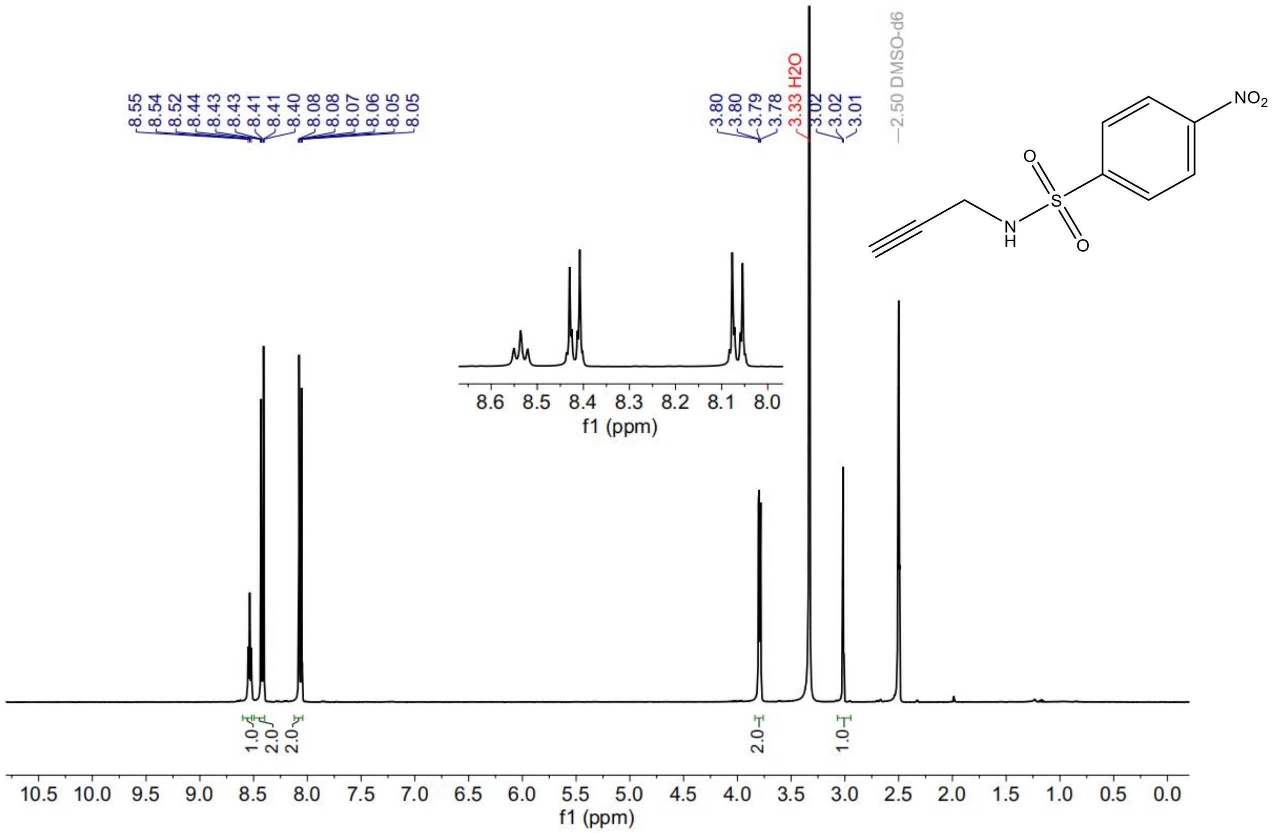

Figure S1: ^{1}H NMR of compound **1** (DMSO-d_6, 400 MHz).

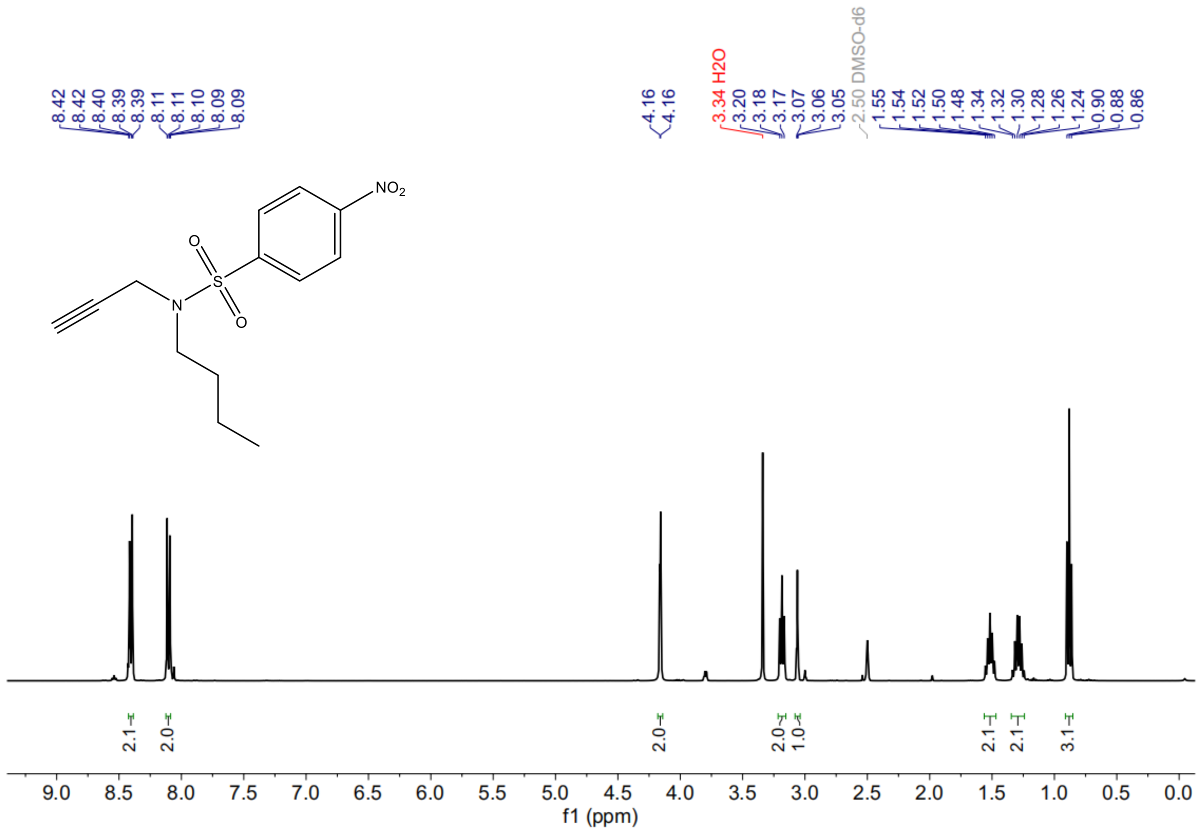

Figure S2: ^{1}H NMR of compound **2** (DMSO-d_6, 400 MHz).

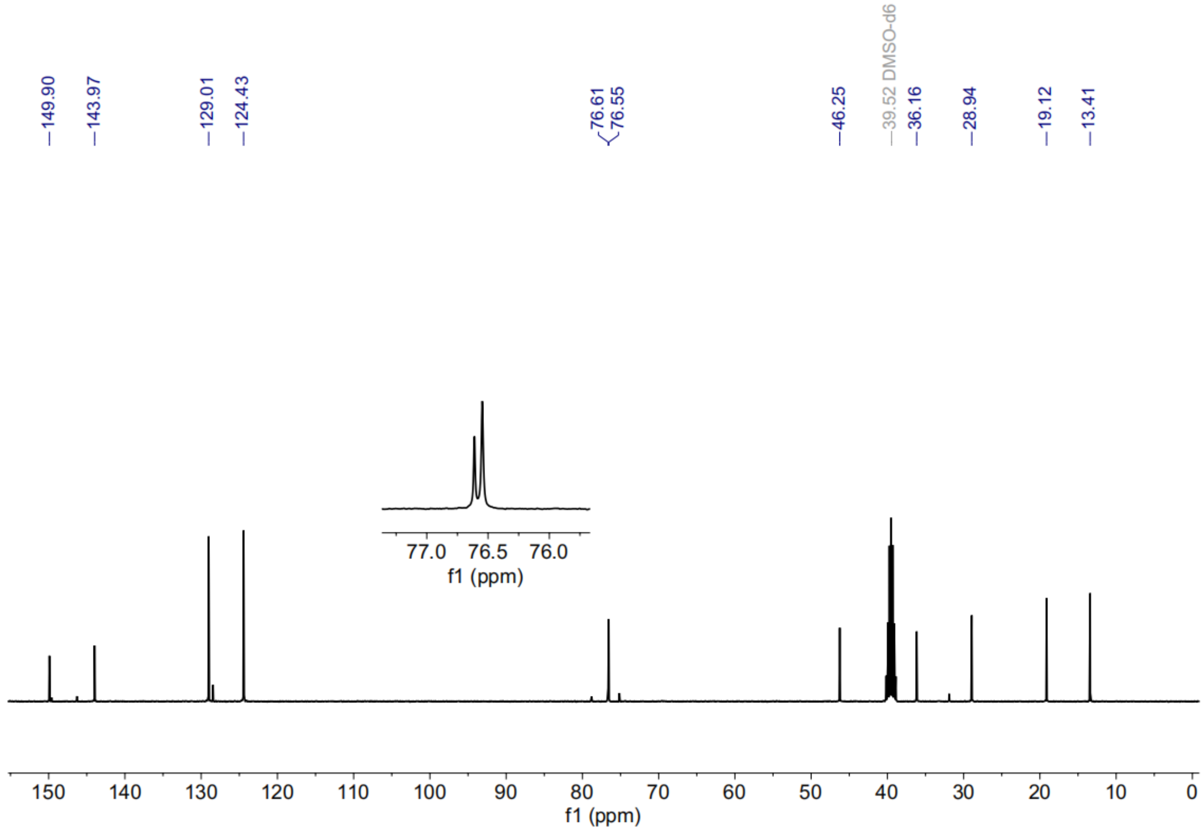

Figure S3: ^{13}C NMR of compound **2** (DMSO-d_6, 101 MHz).

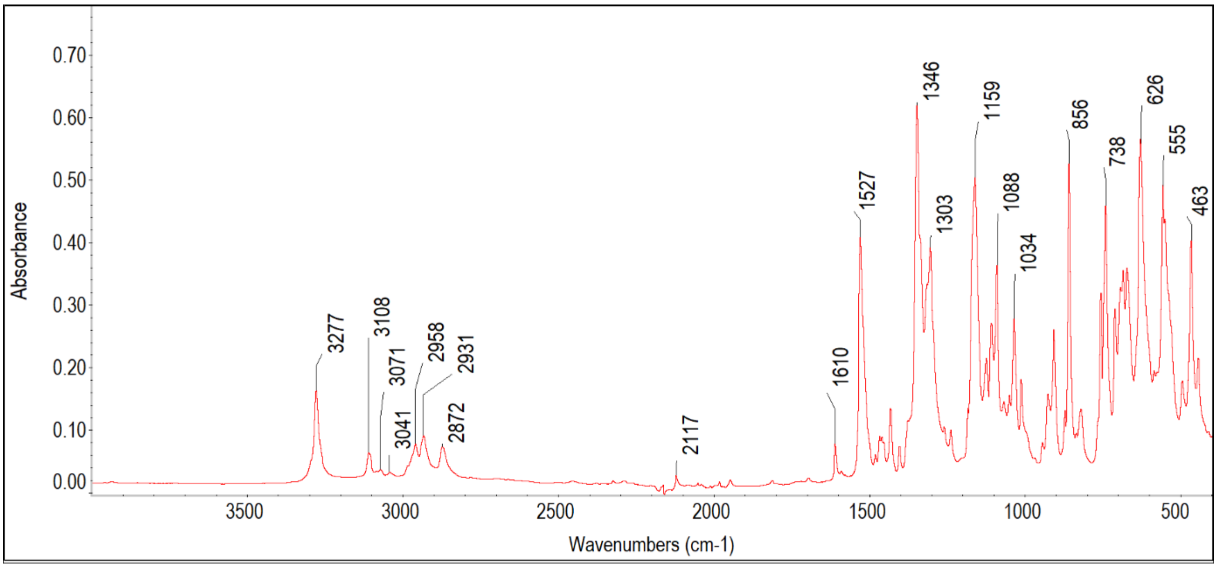

Figure S4: IR spectrum of compound **2** (ATR).

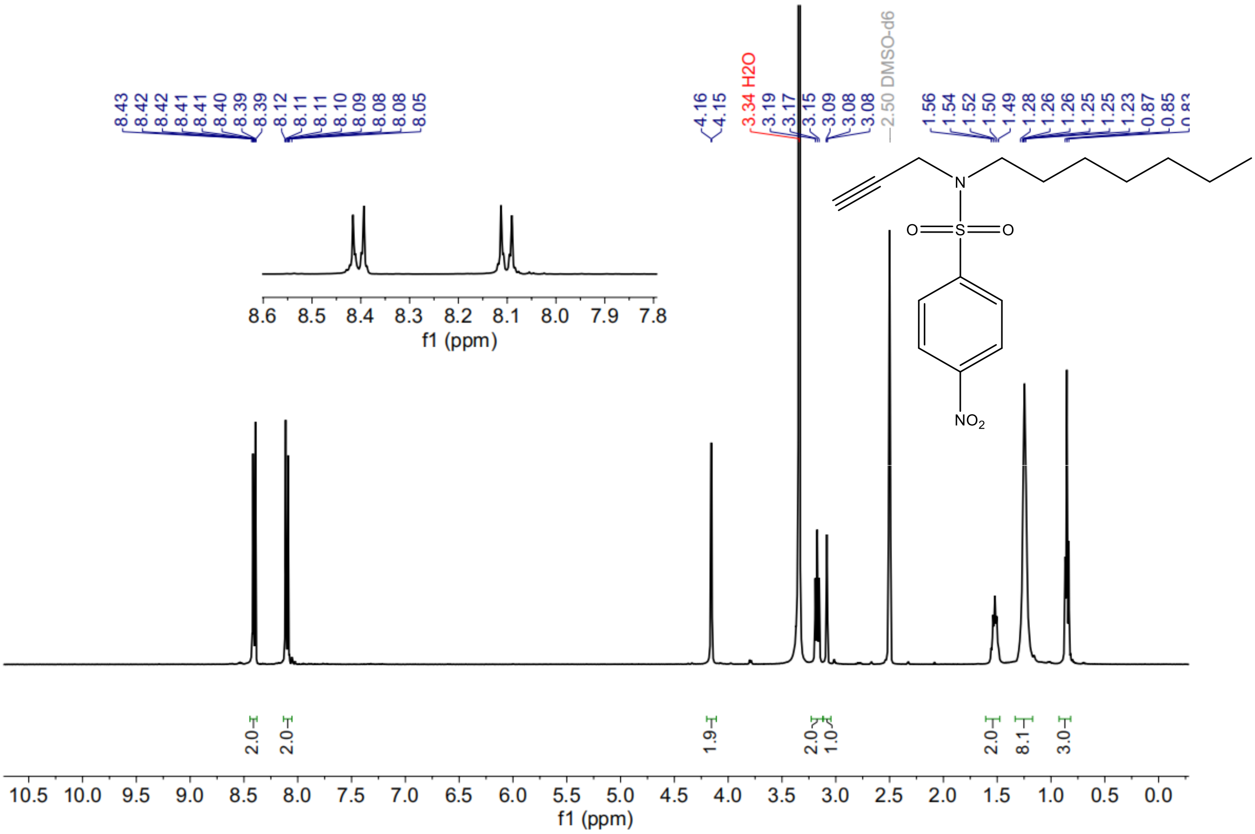

Figure S5: ^{1}H NMR of compound **3** (DMSO-d_6, 400 MHz).

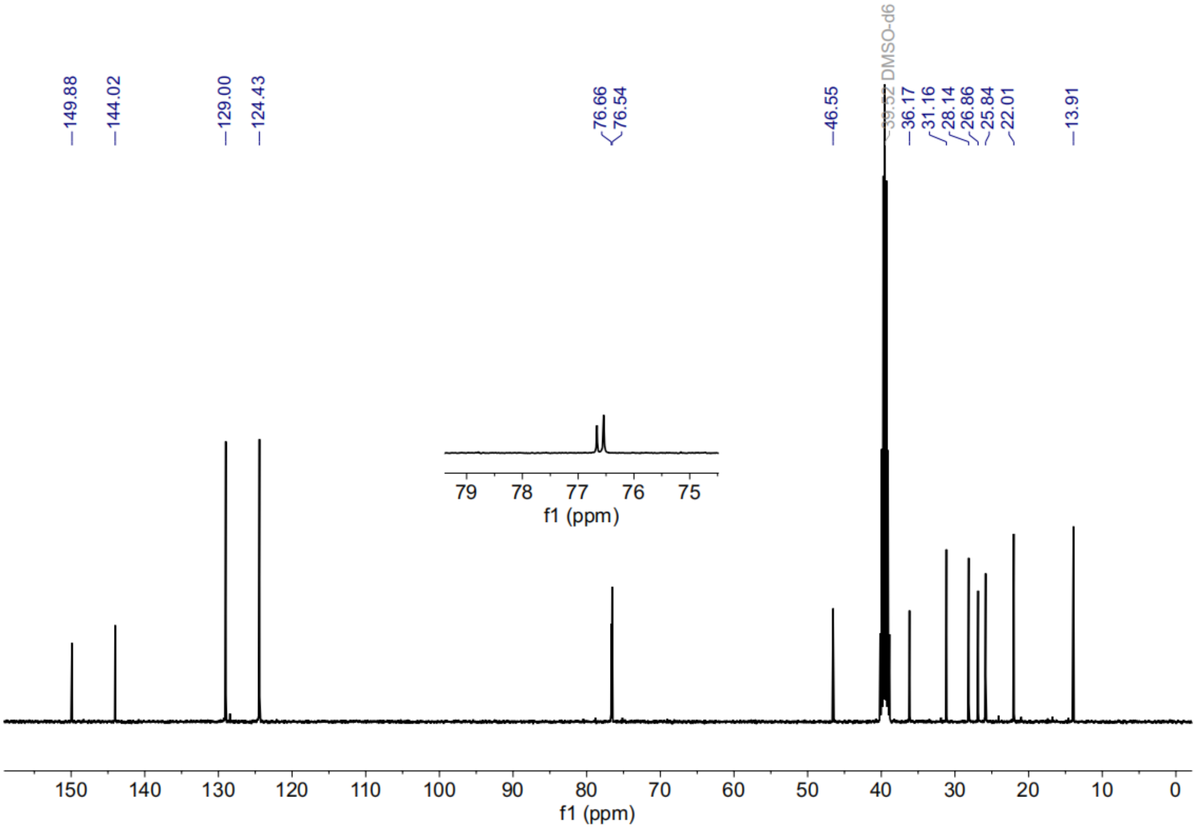

Figure S6: ^{13}C NMR of compound **3** (DMSO-d_6, 101 MHz).

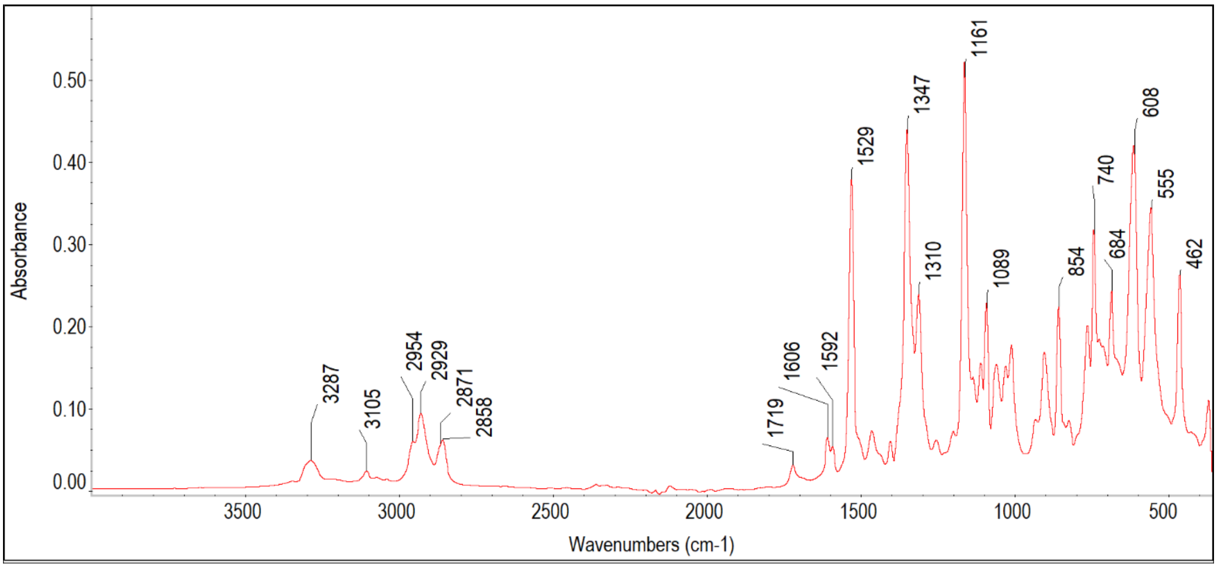

Figure S7: IR spectrum of compound **3** (ATR).

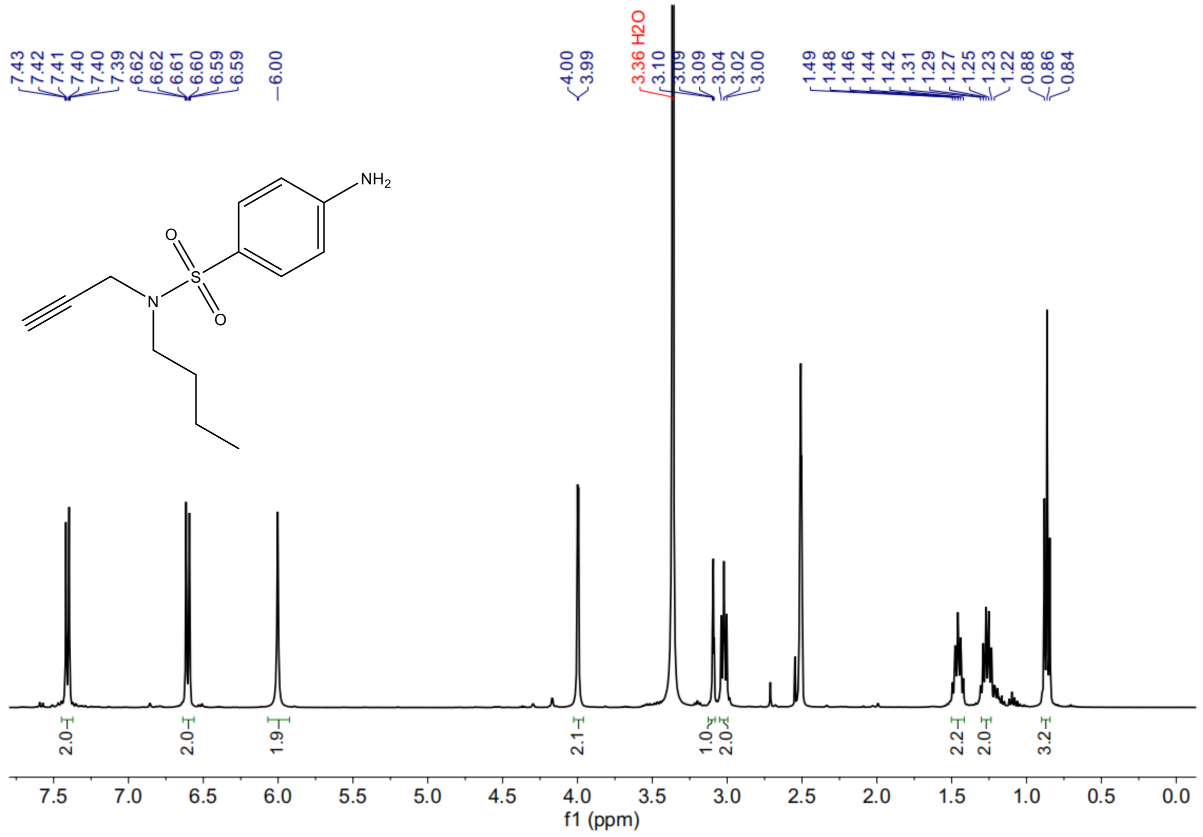

Figure S8: ^{1}H NMR of compound **4** (DMSO-d_6, 400 MHz).

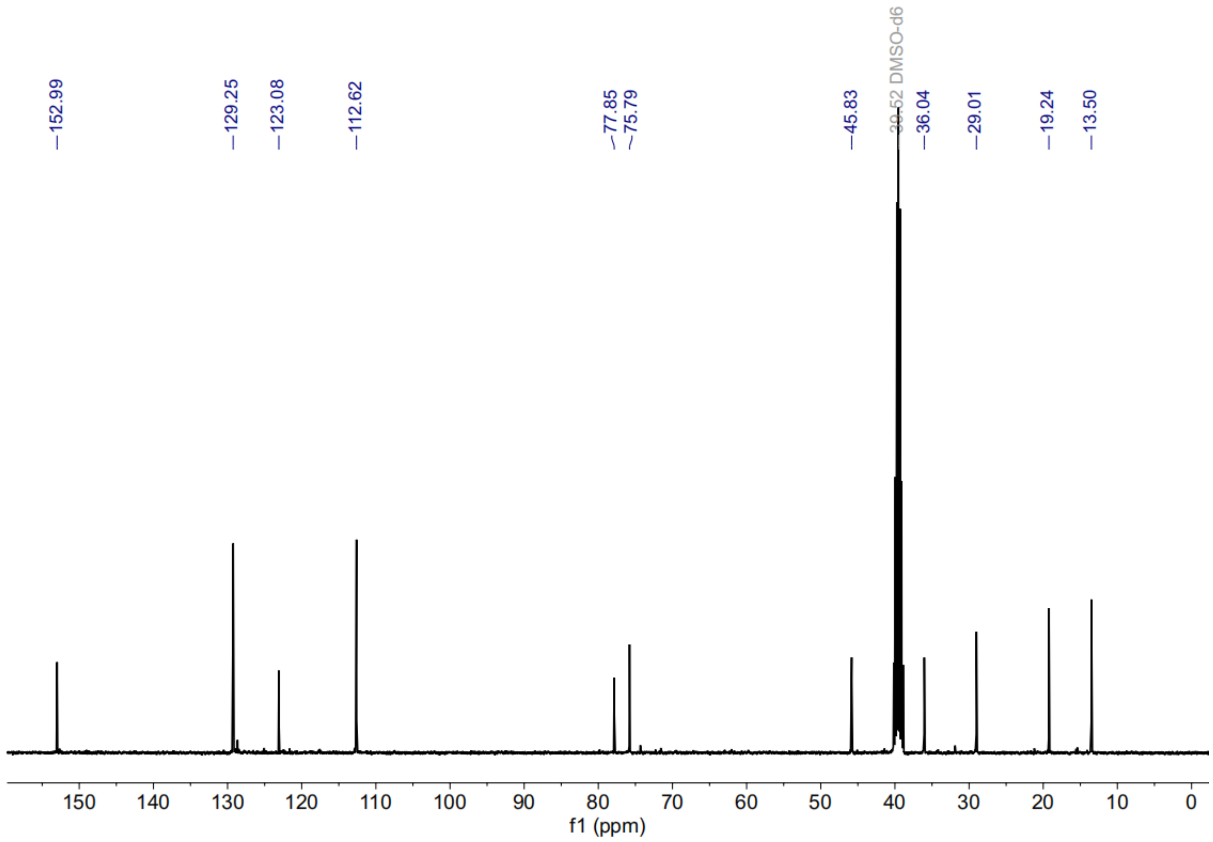

Figure S9: ^{13}C NMR of compound **4** (DMSO-d_6, 101 MHz).

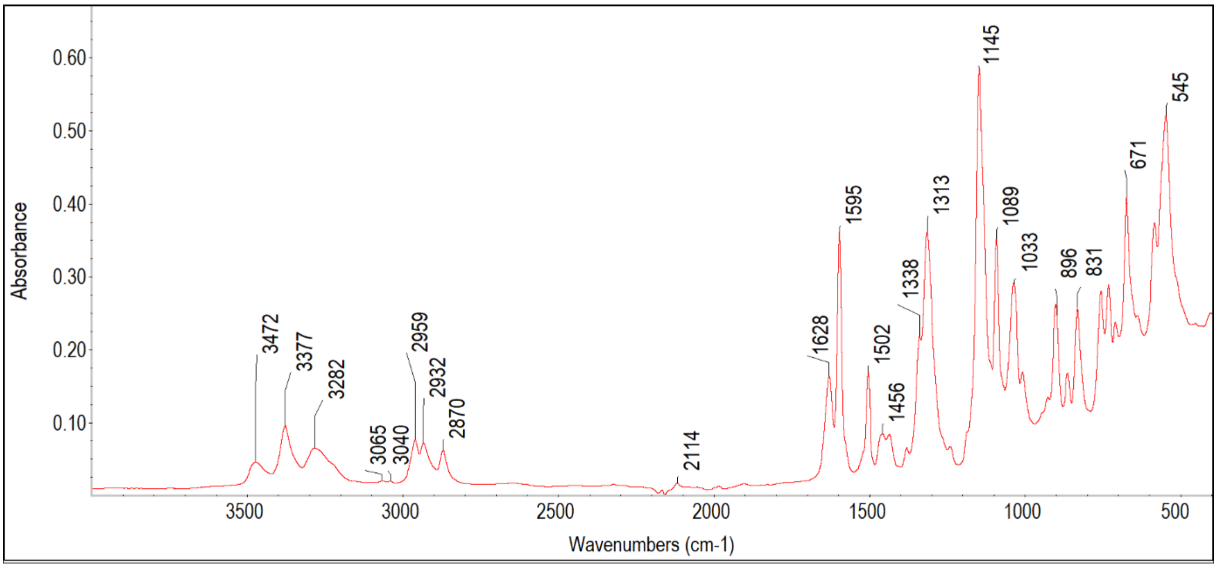

Figure S10: IR spectrum of compound **4** (ATR).

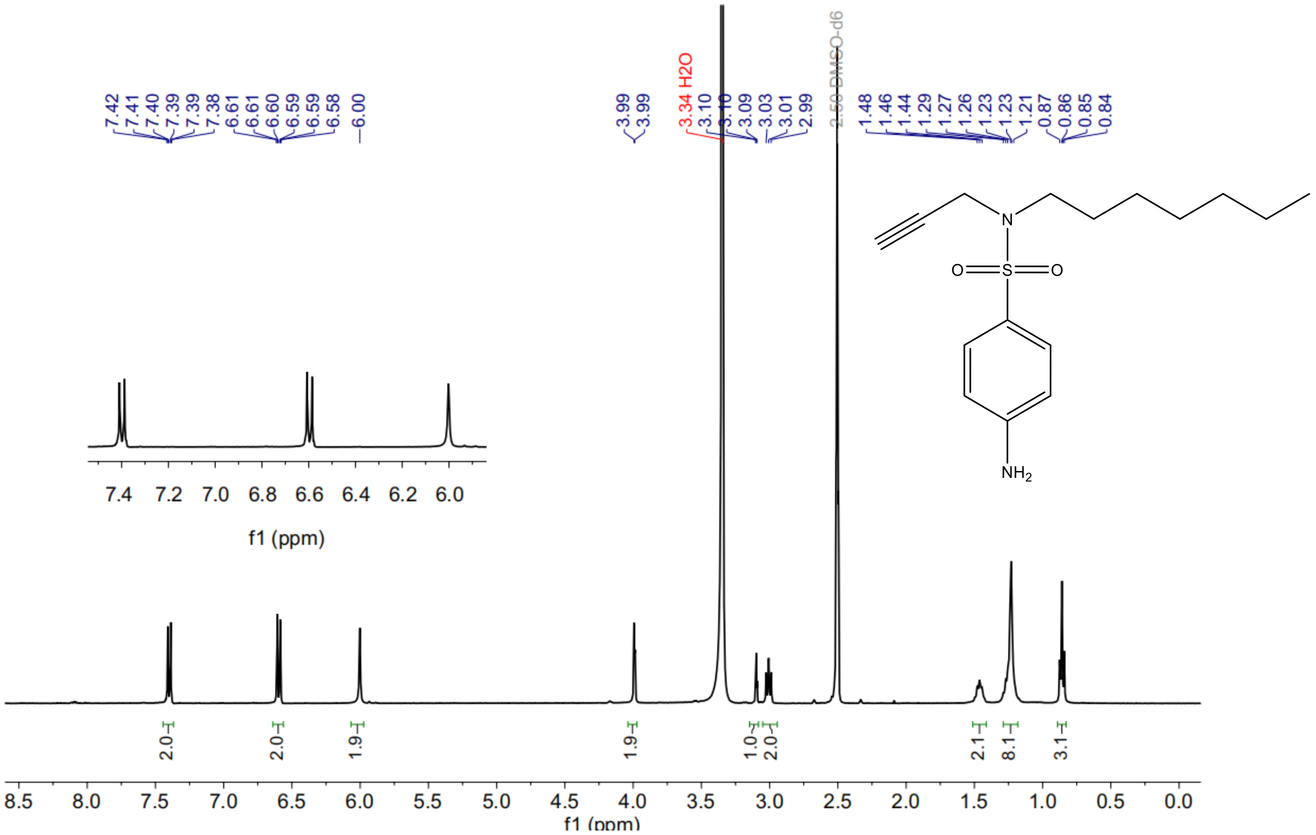

Figure S11: ^{1}H NMR of compound **5** (DMSO-d_6, 400 MHz).

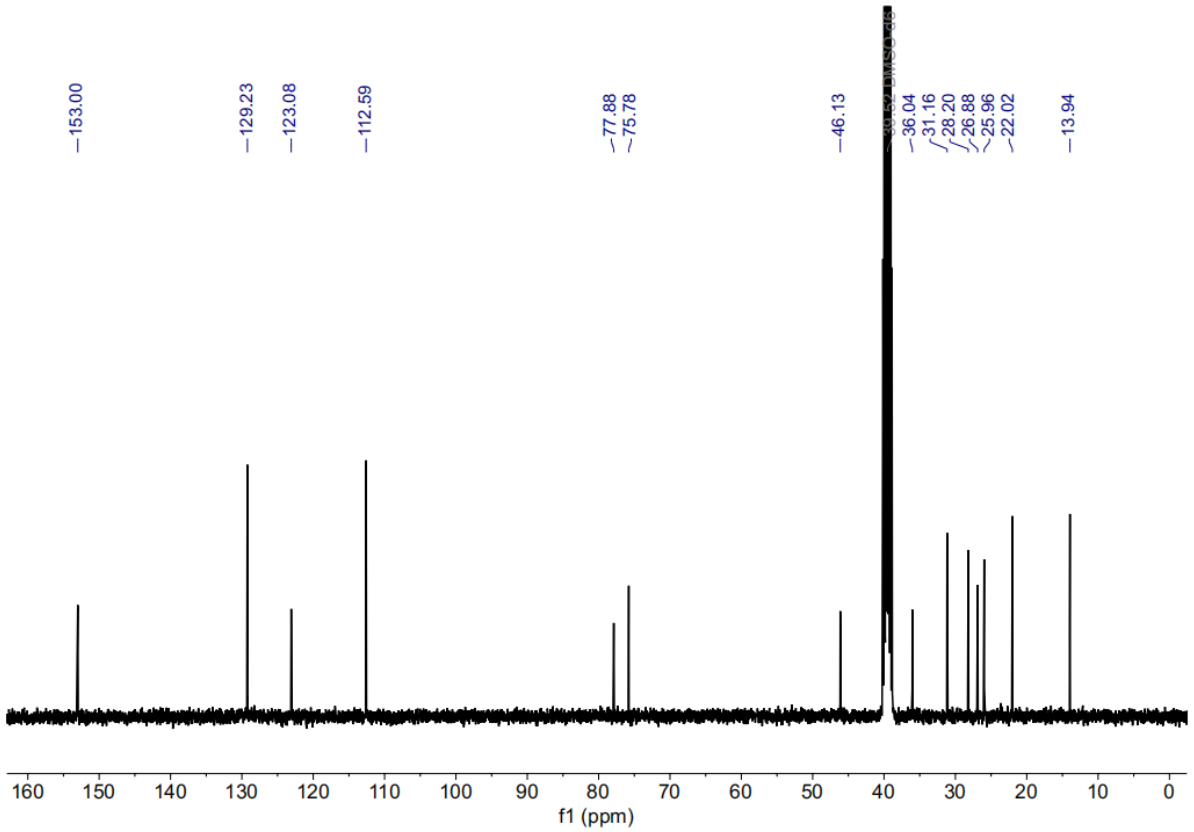

Figure S12: ^{13}C NMR of compound **5** (DMSO-d_6, 101 MHz).

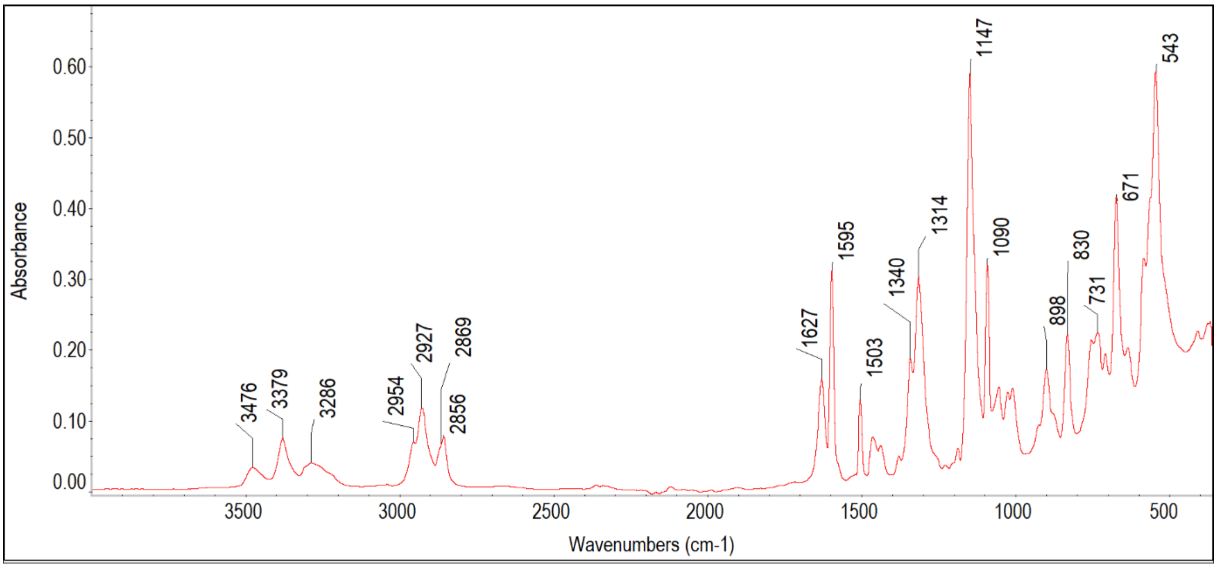

Figure S13: IR spectrum of compound **5** (ATR).

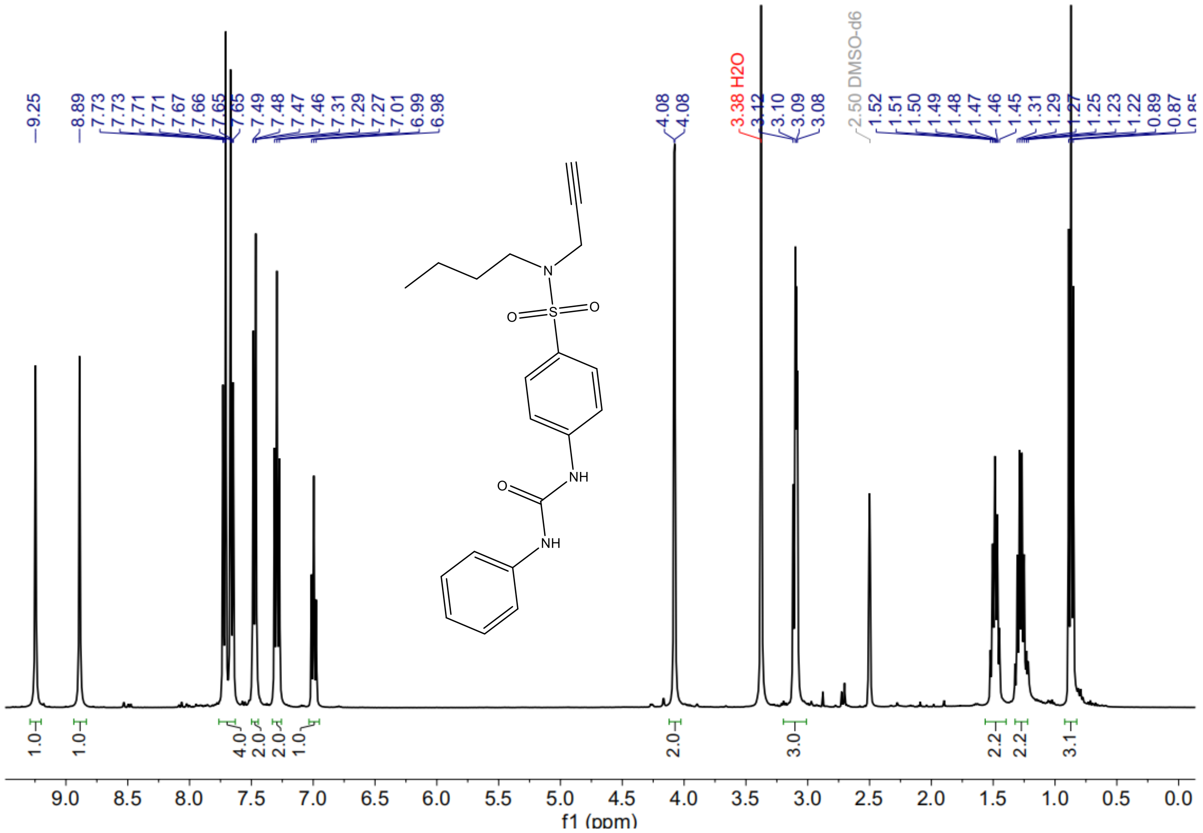

Figure S14: ^{1}H NMR of compound **6** (DMSO-d_6, 400 MHz).

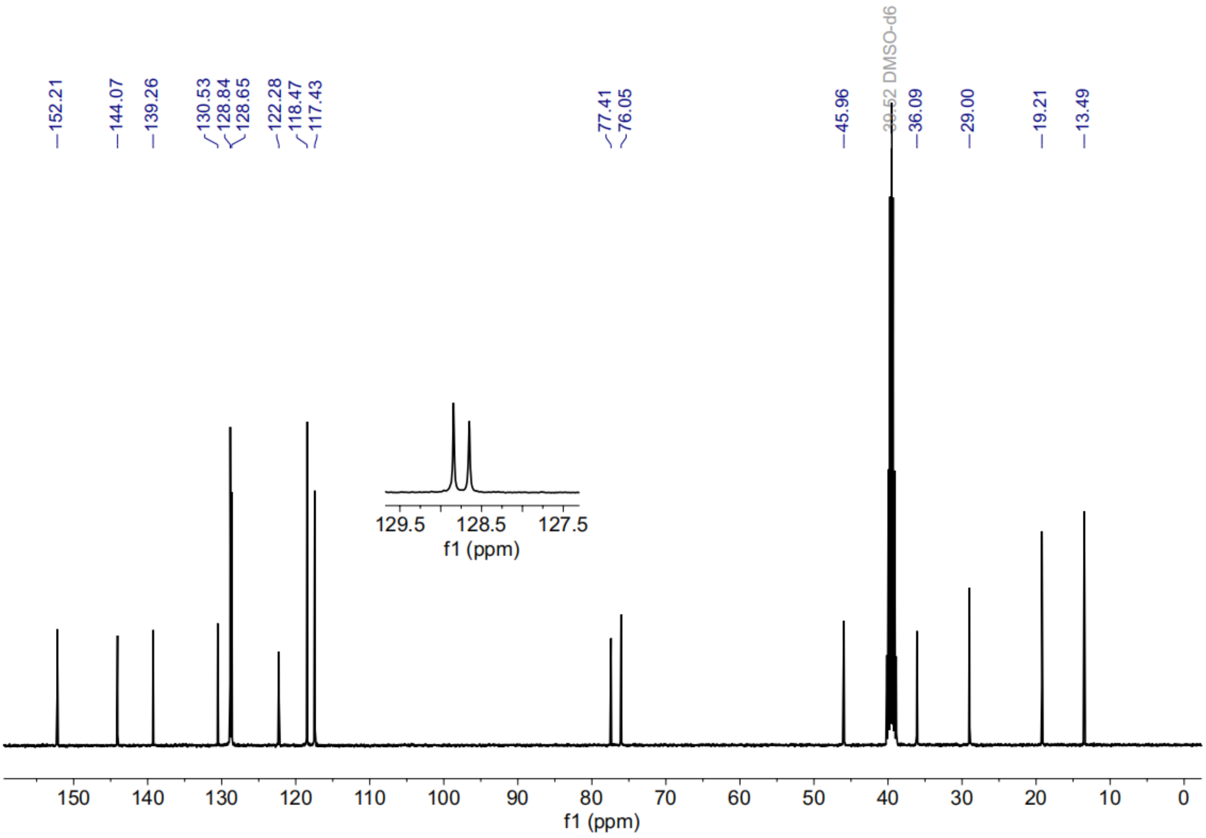

Figure S15: ^{13}C NMR of compound **6** (DMSO-d_6, 101 MHz).

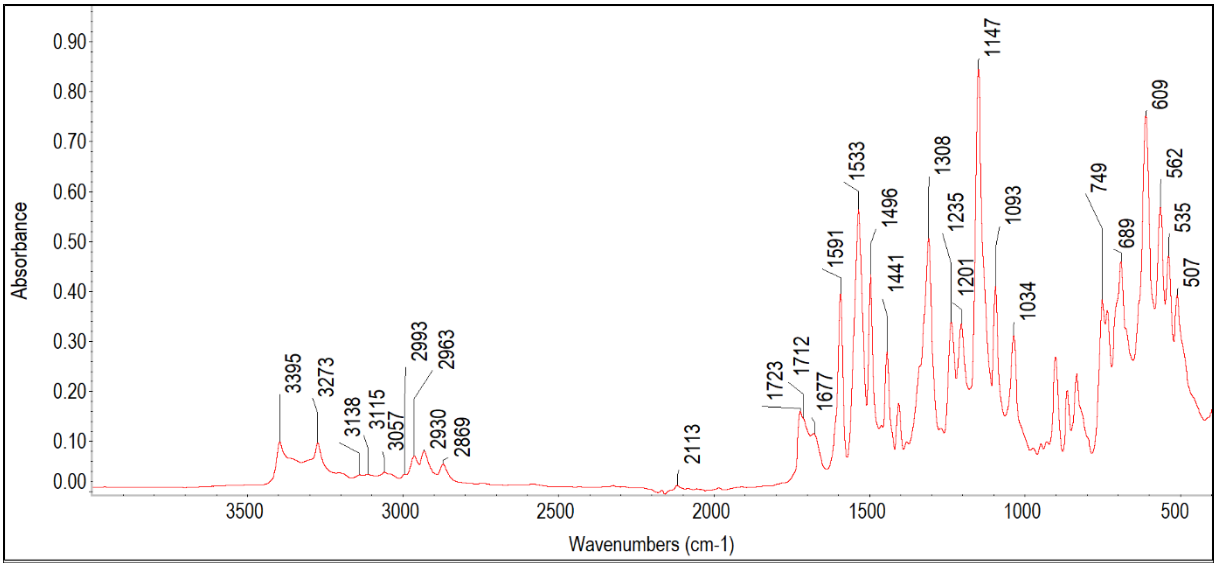

Figure S16: IR spectrum of compound **6** (ATR).

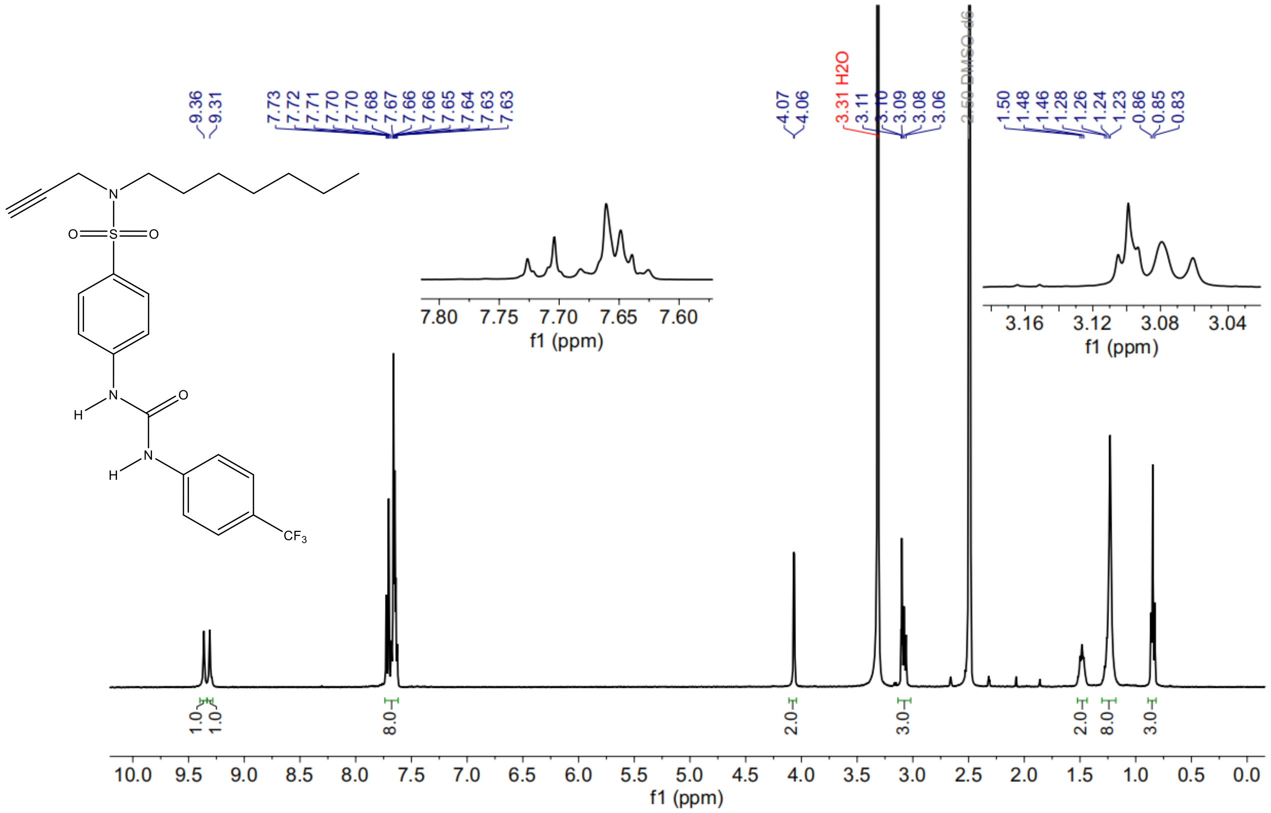

Figure S17: ^{1}H NMR of compound **7** (DMSO-d_6, 400 MHz).

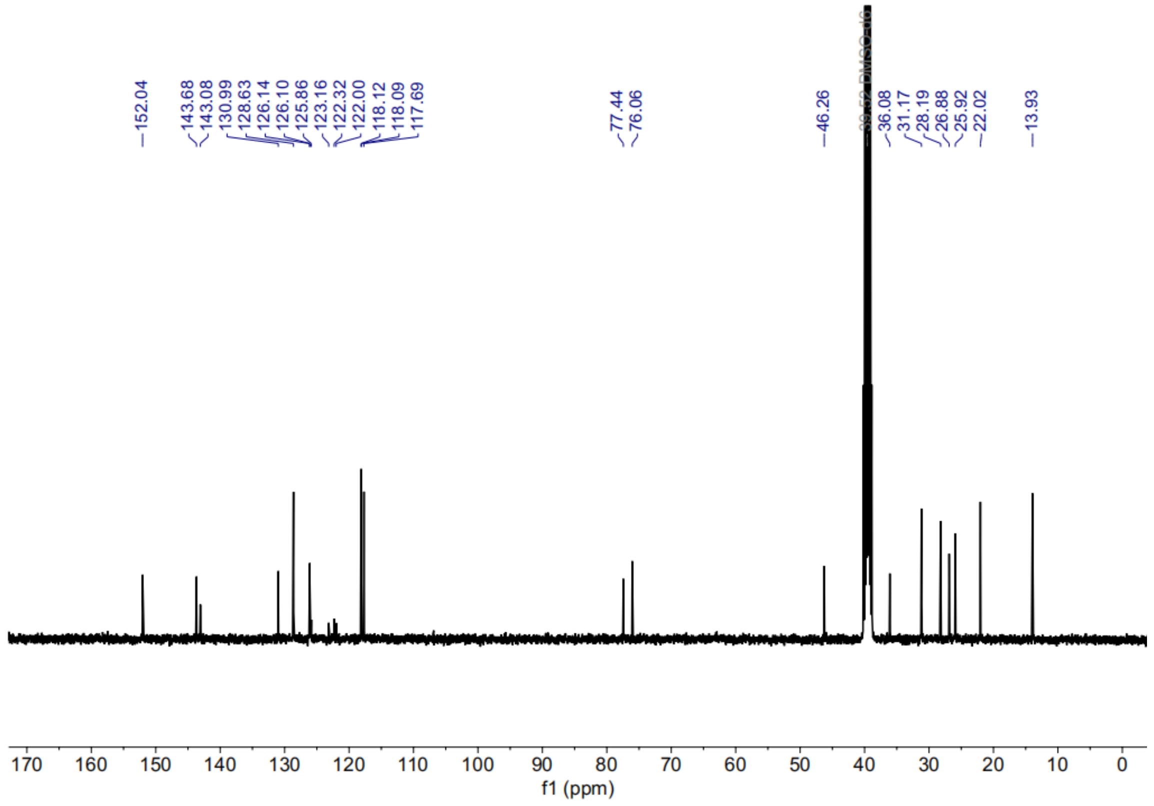

Figure S18: ^{13}C NMR of compound **7** (DMSO-d_6, 101 MHz).

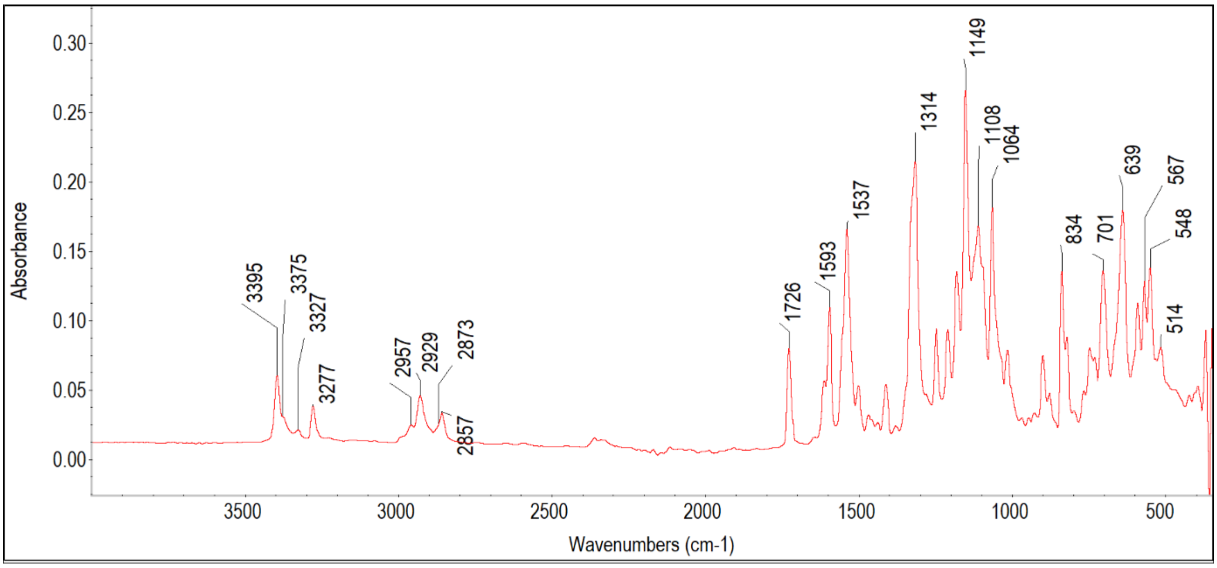

Figure S19: IR spectrum of compound **7** (ATR).

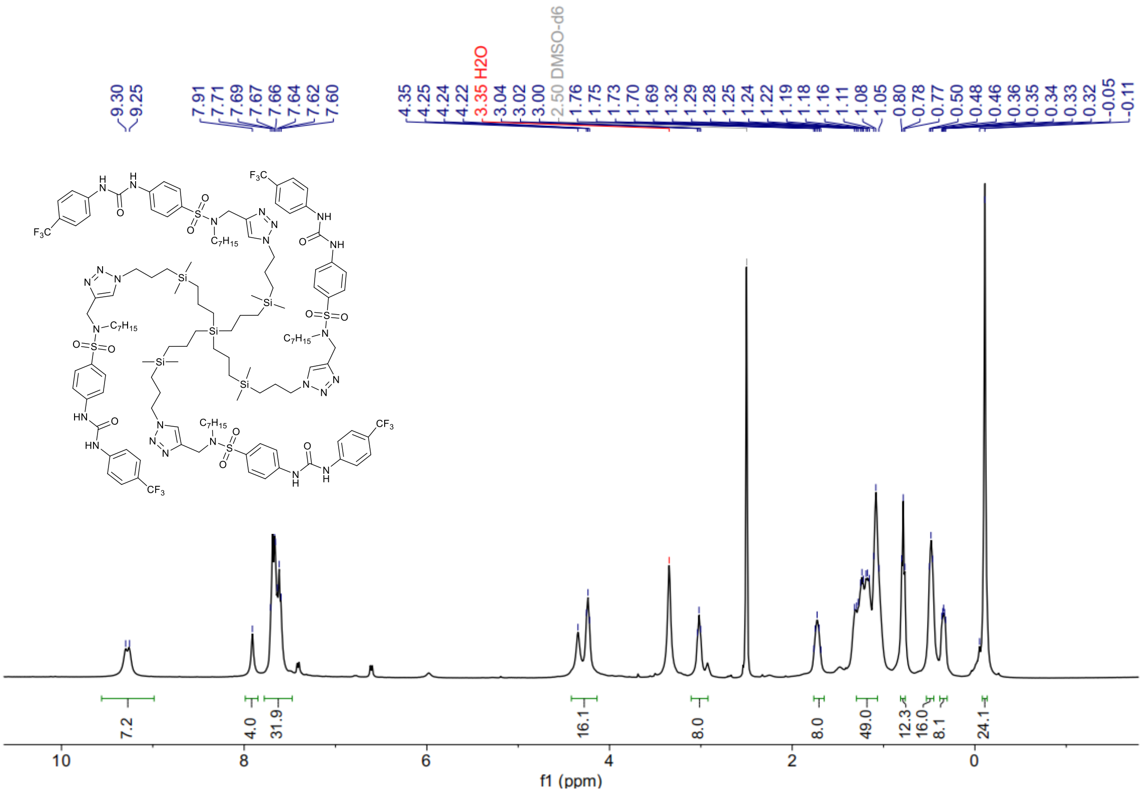

Figure S20: ^{1}H NMR of compound **8** (DMSO-d_6, 400 MHz).

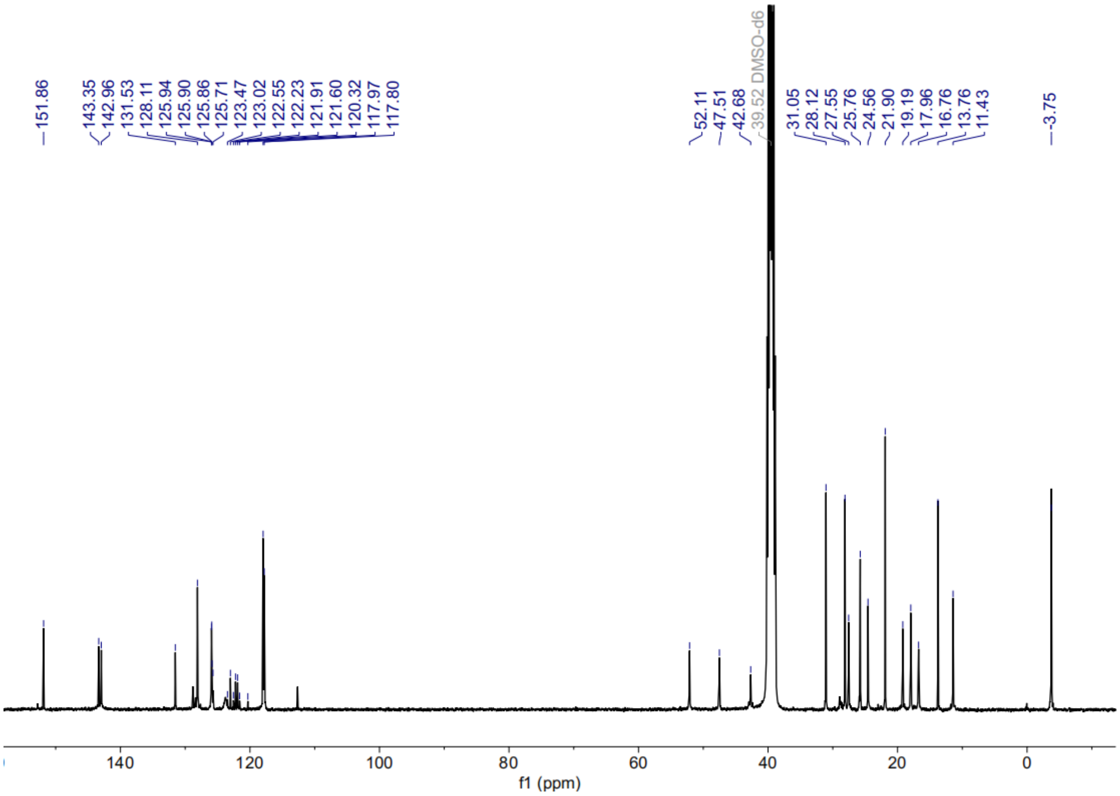

Figure S21: ^{13}C NMR of compound **8** (DMSO-d_6, 101 MHz).

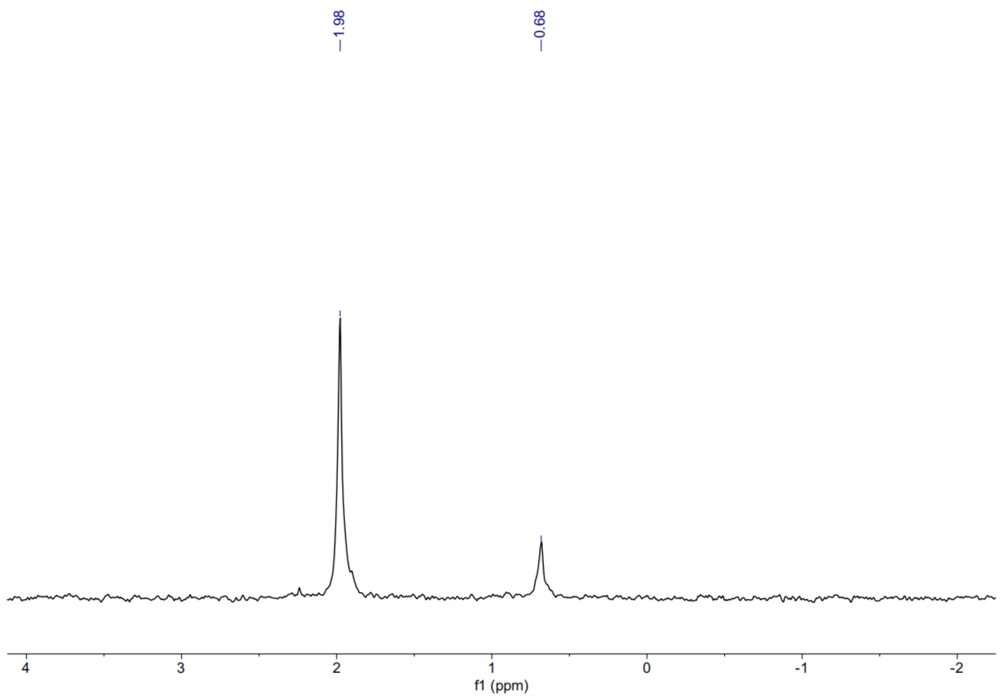

Figure S22: ^{29}Si {^{1}H} NMR of compound **8** (DMSO-d_6, 79 MHz).

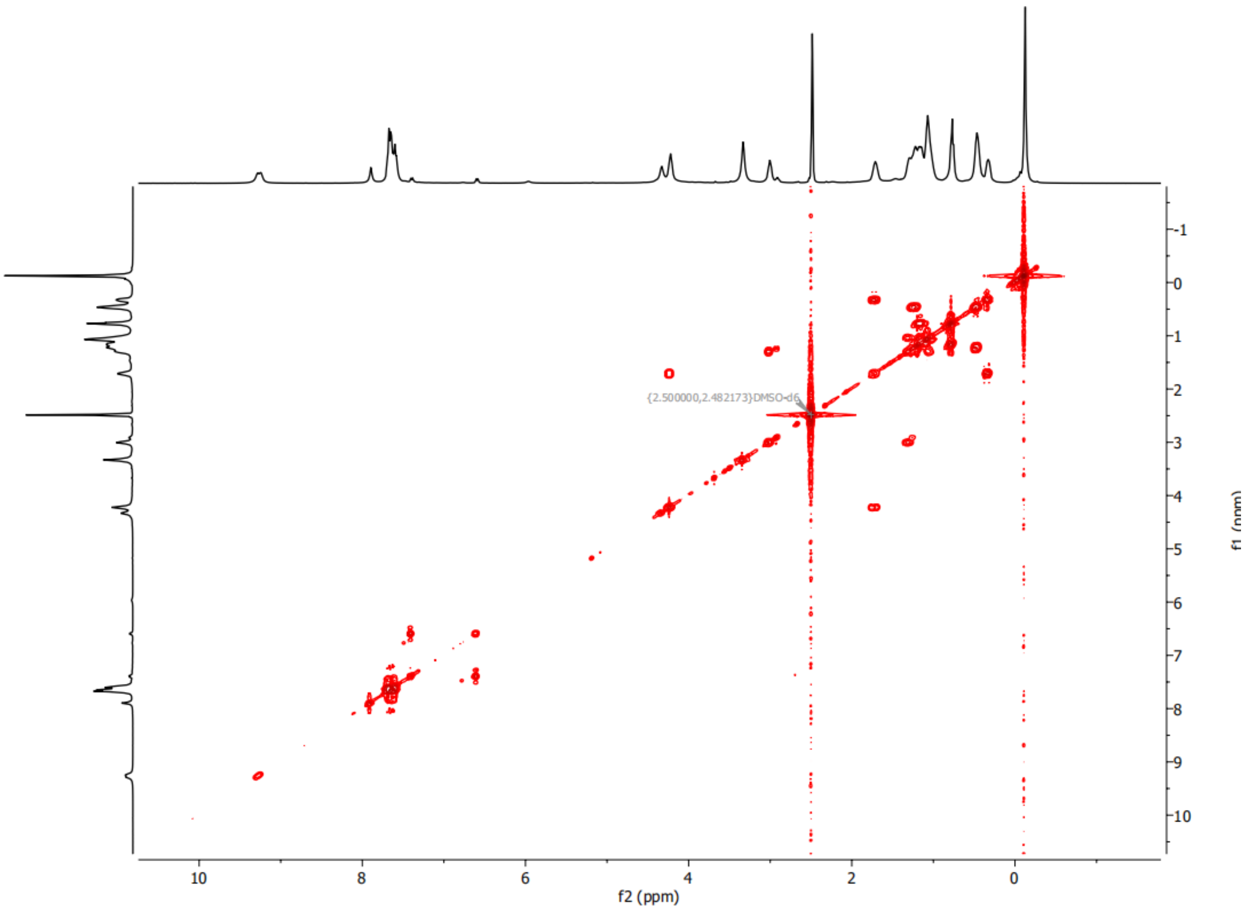

Figure S23: ^{1}H-^{1}H COSY NMR of compound **8** (DMSO-d_6, 400 MHz).

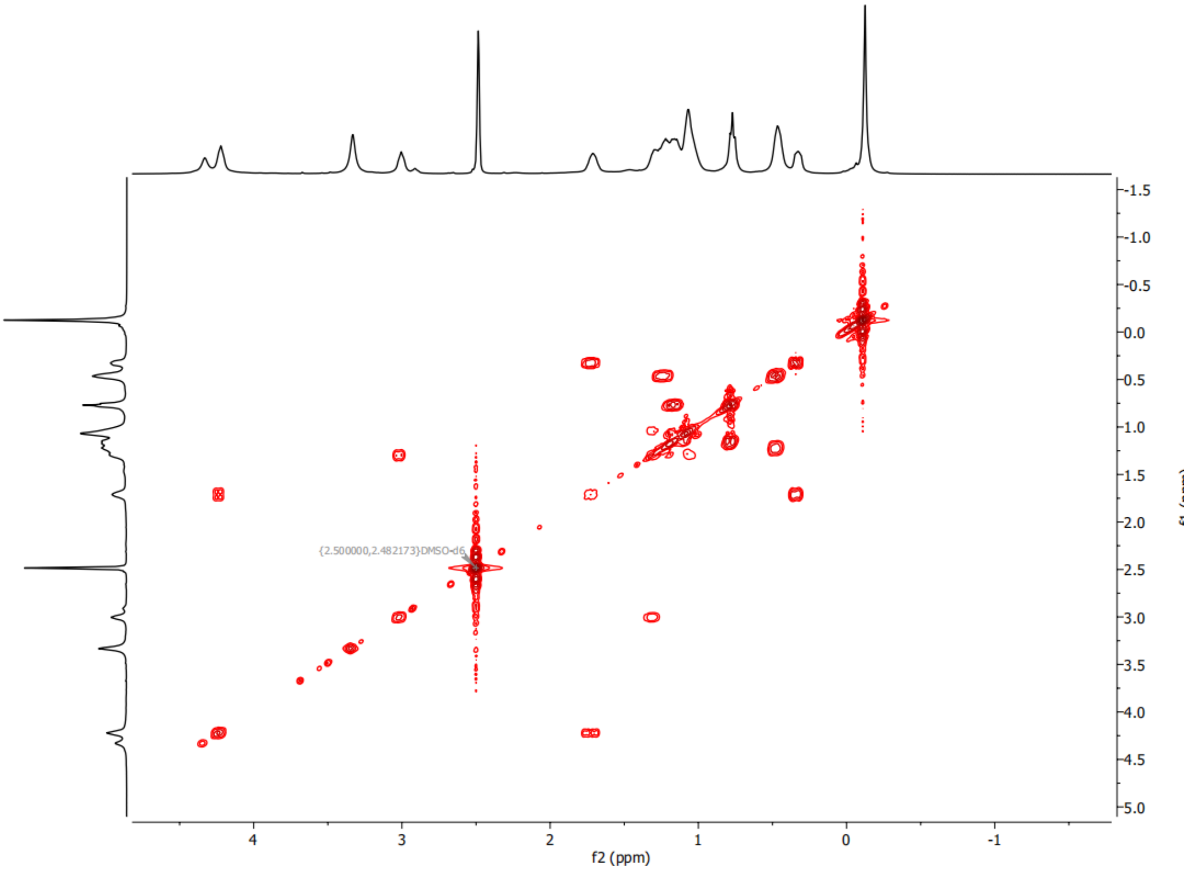

Figure S24: ^{1}H-^{1}H COSY NMR of compound **8** (DMSO-d_6, 400 MHz) – aliphatic region.

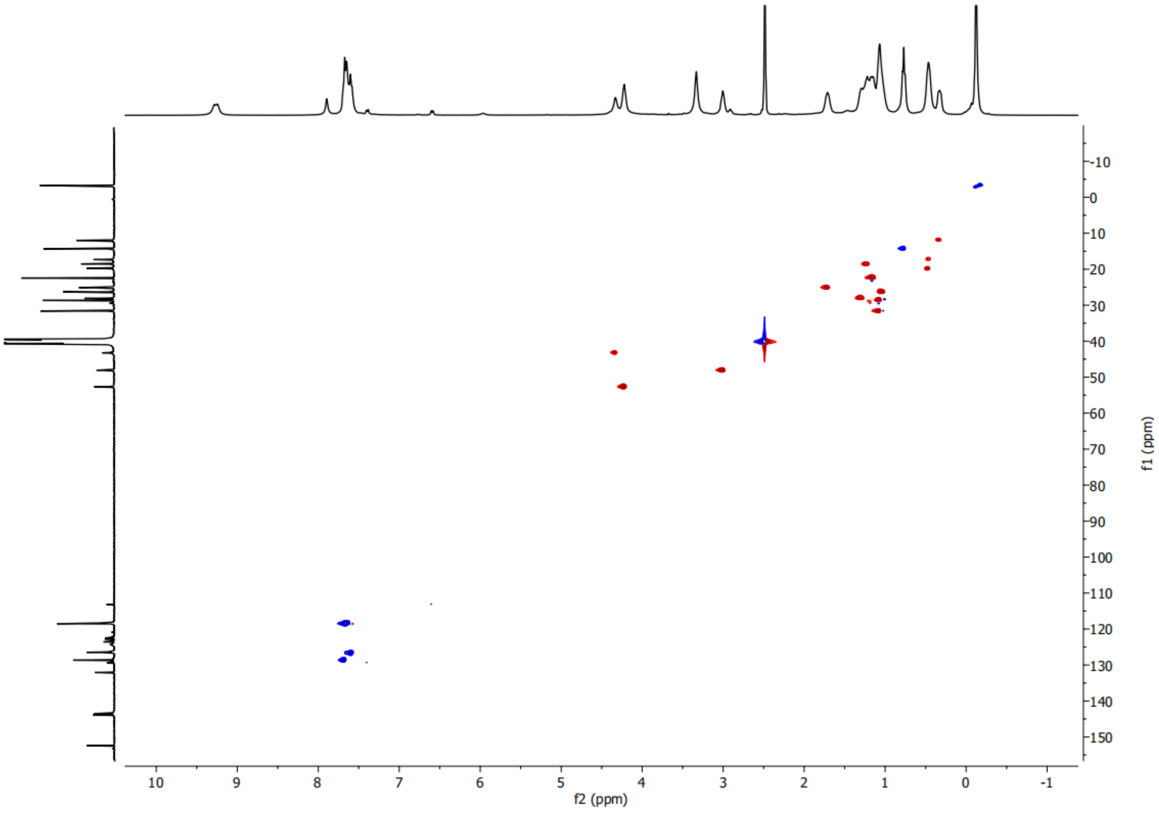

Figure S25: ^{1}H-^{13}C HSQC NMR of compound **8** (400/101 MHz, DMSO-d_6).

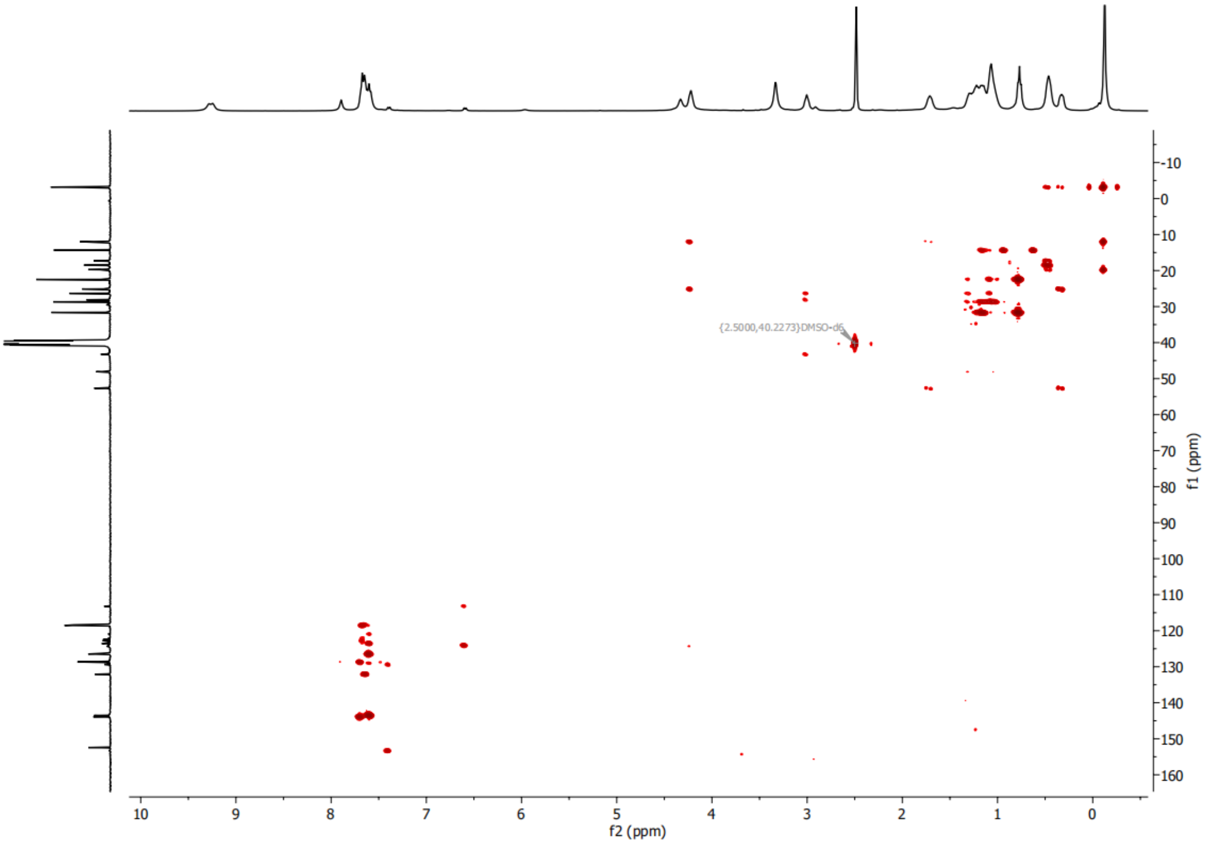

Figure S26: ^{1}H-^{13}C HMBC NMR of compound **8** (400/101 MHz, DMSO-d_6).

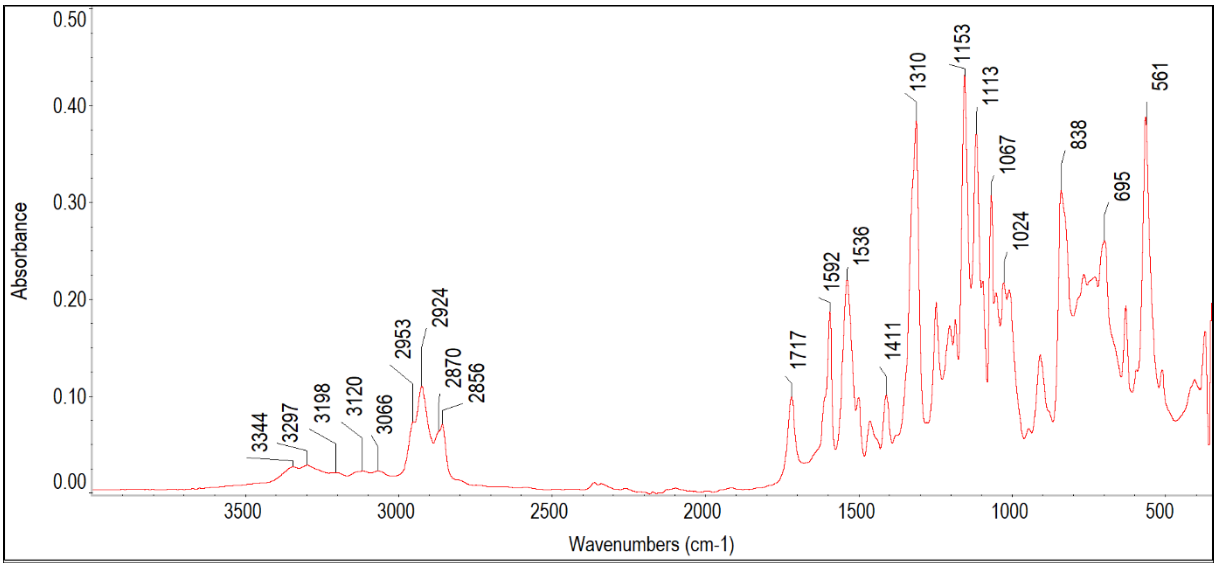

Figure S27: IR spectrum of compound **8** (ATR).

4 X-Ray single crystal diffraction analysis

M = 385.49 g.mol^{-1}, monoclinic system, space group $P2_1/c$, a = 14.8922 (2) Å, b = 15.11541 (19) Å, c = 8.65503 (11) Å, β = 92.4515 (13)°, Z = 4, V = 1946.48 (5) Å^3, D_{calc} = 1.315 g.cm^{-3}, μ(Cu Kα) = 1.69 mm^{-1}, crystal dimensions of 0.55 × 0.19 × 0.04 mm. T = 95 (2) K, R_{obs} = 0.037, wR_{all} = 0.106, indep. refln. = 3940, θ_{max} = 74.6°), ref. par. no. = 252, ref. res. no. = 8. CCDC 228879.

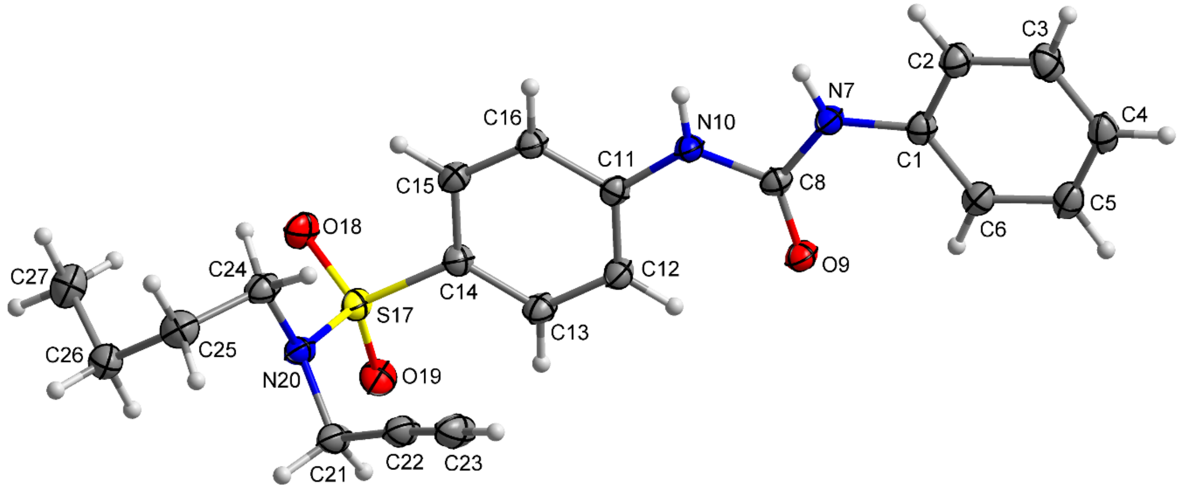

Figure S28. The numbering scheme of **6** with ADPs displayed at 50% probability level.

M = 495.56 g.mol^{-1}, monoclinic system, space group $C2/c$, a = 38.7601 (19) Å, b = 15.0572 (7) Å, c = 8.6258 (4) Å, β = 98.942 (2)°, Z = 8, V = 4973.0 (4) Å^3, D_{calc} = 1.324 g.cm^{-3}, μ(Cu Kα) = 1.62 mm^{-1}, crystal dimensions of 0.58 × 0.16 × 0.03 mm. T = 180 (2) K, R_{obs} = 0.051, wR_{all} = 0.140, indep. refln. = 4752, θ_{max} = 70.4°), ref. par. no. = 407, ref. res. no. = 157. CCDC 2288792. The disordered heptyl and trifluoromethyl groups were refined with restrained geometry. The heptyl group disorder is symmetry related therefore its occupancy ratio is fixed at 500:500, while the trifluoromethyl group was refined with sum of occupancies constrained to full, resulting in occupancy ratio of 544(13):456(13).

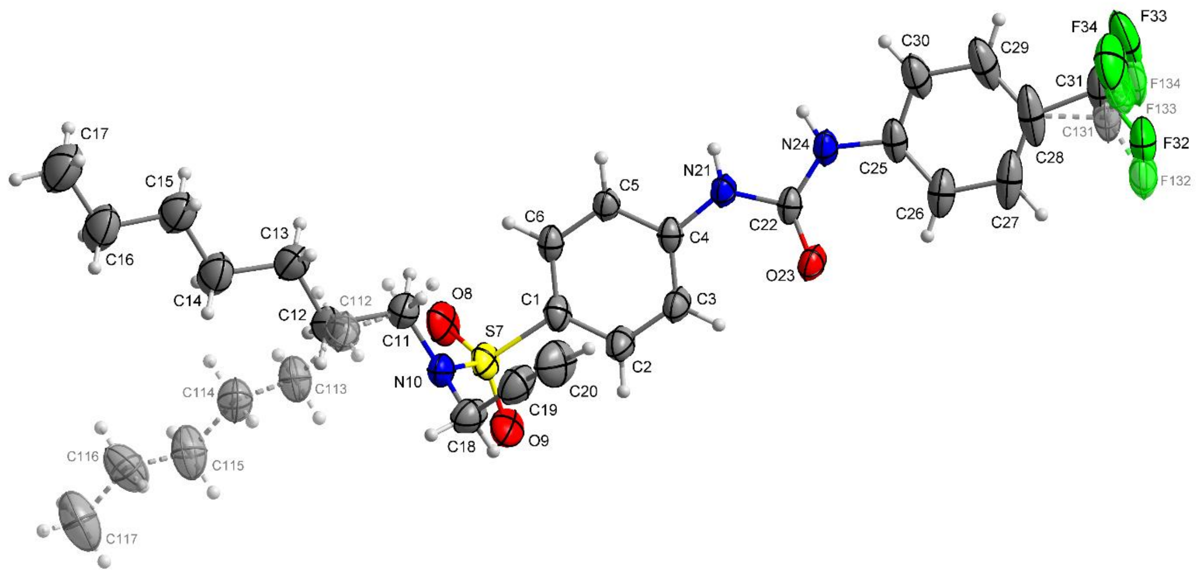

Figure S29. The numbering scheme of **7** with ADPs displayed at 50% probability level. The second disorder positions depicted as transparent.

5 Dynamic light scattering

Table S1: Data obtained by DLS measurements for receptor **7** in $CHCl_3$.

c **7** (mM)	*c* $H_2PO_4^-$ (mM)	Effective diameter (nm)	Polydispersity
1.95	-	2 000	0.25
1.67	-	1 480	0.27
1.17	-	1 400	0.32
0.98	-	1 360	0.31
1.95	2.0	990	0.21

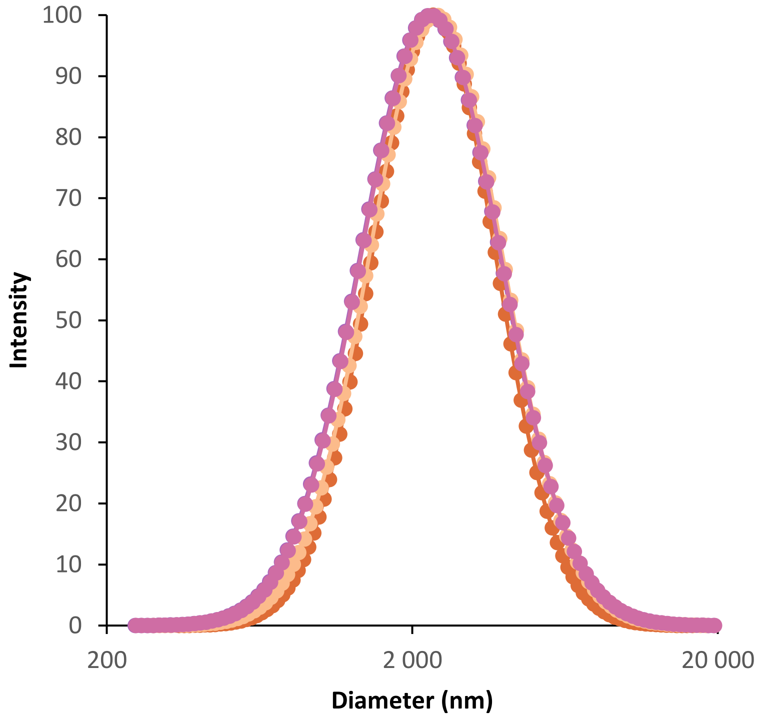

Figure S30: Sample recording of a series of DLS measurements obtained for receptor **7** (1.95 mM, $CHCl_3$).

6 Titration experiments

Dilution experiments

Table S2: The obtained values of aggregation constants K_E and dimerization constant K_d determined by ^{1}H NMR dilution of receptor **7** in DMSO-d_6 or $CDCl_3$ at 25 °C, evaluated for CoEK aggregation model and EK aggregation model.

Model	Solvent	K_d [M]$^{-1}$	K_E [M]$^{-1}$	ρ
CoEK aggregation	DMSO-d_6	40 ± 5 %	80 ± 10 %	0.48 ± 30 %
EK aggregation		20 ± 2.5 %	40 ± 5 %	-
CoEK aggregation	$CDCl_3$	130 ± 3 %	260 ± 6 %	1.77 ± 15 %
EK aggregation		170 ± 2.5 %	340 ± 5 %	-

ρ – Cooperativity factor[S13]

c (**7**) mmol/L	δ_{NH} (ppm)
25.6	9.4705
17.1	9.4453
12.8	9.4303
10.2	9.4280
8.53	9.4176
6.82	9.4038
5.68	9.3971
3.79	9.3850
2.53	9.3748
1.26	9.3646
0.632	9.3586

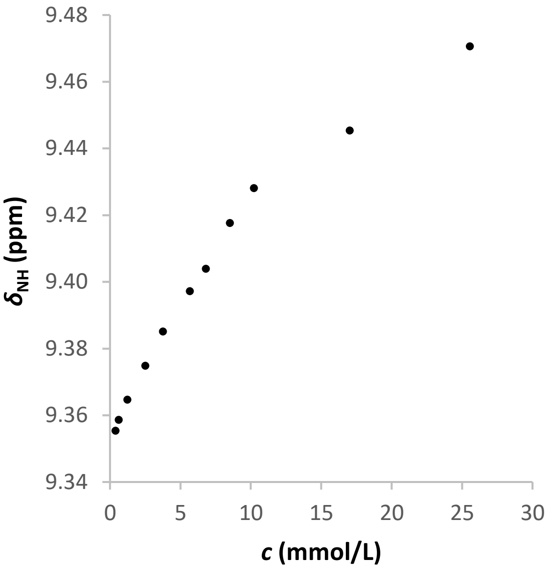

Figure S31: Chemical shifts of urea N*H* signal of **7** as a function of its concentration during dilution measurement in DMSO-d_6, t = 25 °C.

LINK for CoEK aggregation model: http://app.supramolecular.org/bindfit/view/13ed7f80-71d1-46c9-af12-42326ab7097d

LINK for EK aggregation model: http://app.supramolecular.org/bindfit/view/69be1015-dc76-41f3-b0b7-ba63a2464f10

c (**7**) mmol/L	δ_{NH} (ppm)
25.5	7.68
17.0	7.58
12.7	7.54
10.2	7.48
8.50	7.45
6.80	7.41
3.78	7.28
2.52	7.19
1.26	7.04
0.630	6.94
0.420	6.86

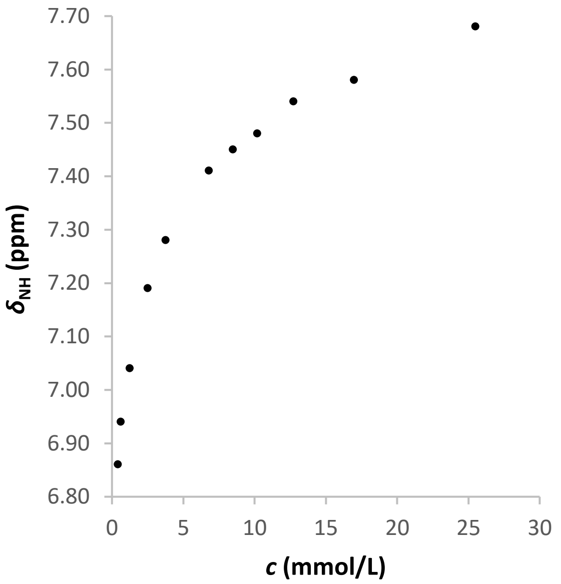

Figure S32: Chemical shifts of urea N*H* signal of **7** as a function of its concentration during dilution measurement in $CDCl_3$, t = 25 °C.

LINK for CoEK aggregation model: http://app.supramolecular.org/bindfit/view/e15d7f6c-fc8d-432c-b84e-ebdbc02b221c

LINK for EK aggregation model: http://app.supramolecular.org/bindfit/view/752504db-c9e2-4e1a-92b3-c23bd374bb58

Complexation study

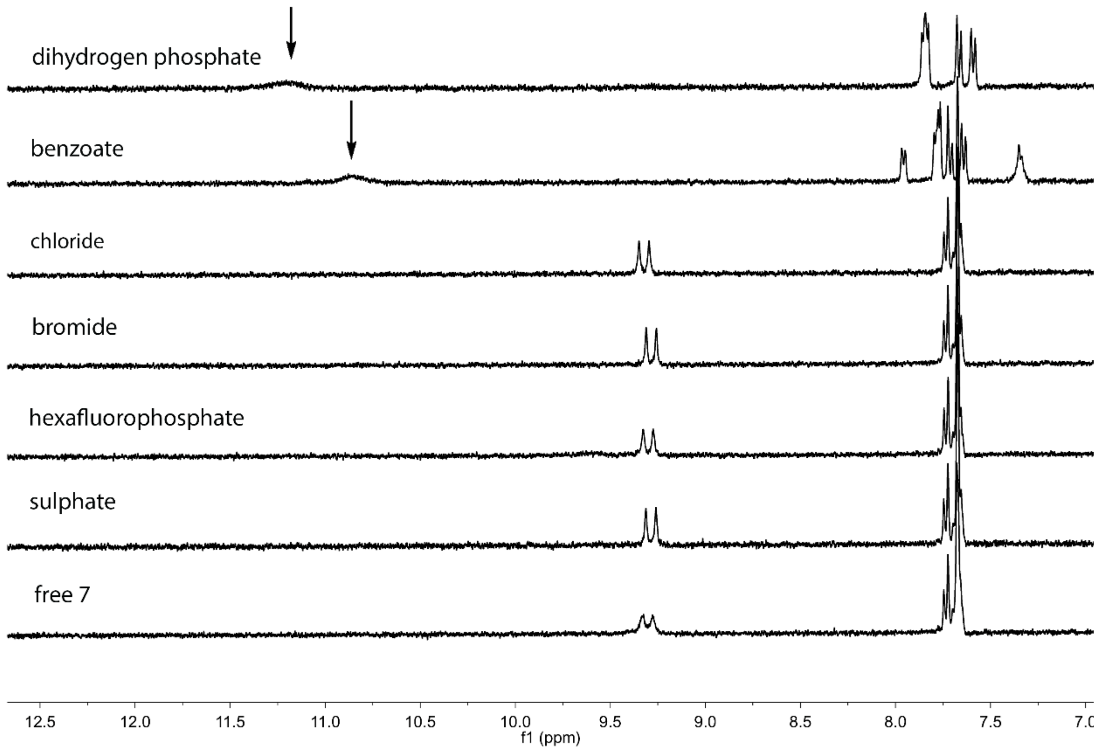

Figure S33: Aromatic and N*H* region in ^{1}H NMR spectra of receptor **7** (0.5 mM, DMSO-d_6, 25 °C) after the addition of 1 equivalent of anion in the form of TBA$^+$ salt.

c (**6**) mmol/L	*c* ($TBA^+H_2PO_4^-$) mmol/L	δ_{ArH} (ppm)
0.644	0	7.0061
0.644	0.126	6.9905
0.644	0.248	6.9734
0.644	0.364	6.9608
0.644	0.585	6.9424
0.644	0.791	6.9323
0.644	1.07	6.9209
0.644	1.49	6.9125
0.644	1.84	6.9073
0.644	2.41	6.9033
0.644	2.86	6.9011
0.644	6.44	6.8947

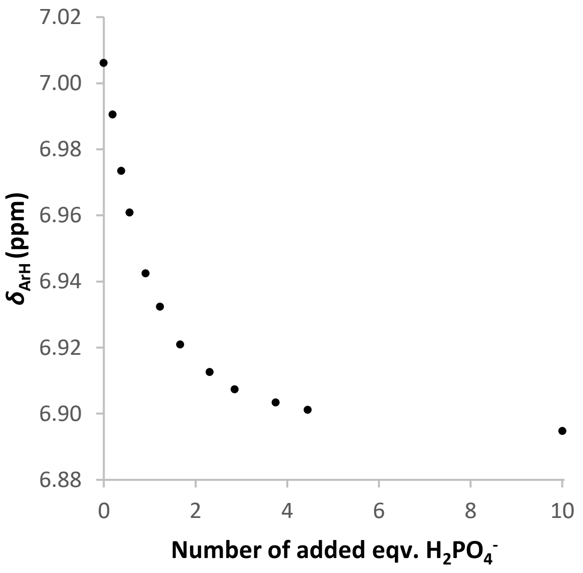

Figure S34: ^{1}H NMR titration of **6** by $TBA^+H_2PO_4^-$ (DMSO-d_6, 400 MHz).

LINK: http://app.supramolecular.org/bindfit/view/ce8c0263-2be7-44f5-b979-4f10157948eb

c (**6**) mmol/L	c (TBA^+BzO^-) mmol/L	δ_{NH} (ppm)
0.644	0	9.2150
0.644	0.125	9.6462
0.644	0.246	10.0690
0.644	0.362	10.4126
0.644	0.582	10.9311
0.644	0.786	11.2633
0.644	1.07	11.5797
0.644	1.48	11.8446
0.644	1.83	11.9912
0.644	2.40	12.1466
0.644	2.84	12.2209
0.644	6.40	12.4908

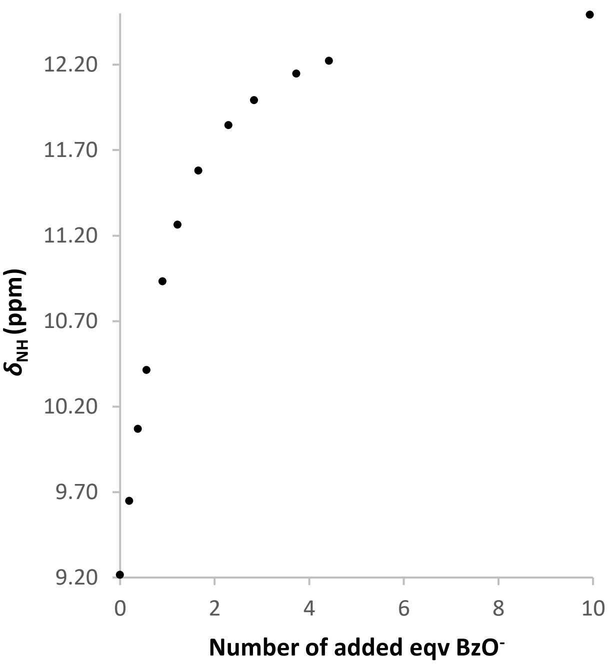

Figure S35: ^{1}H NMR titration of **6** by TBA^+BzO^- (DMSO-d_6, 400 MHz).

LINK: http://app.supramolecular.org/bindfit/view/88a3f749-e218-4550-9873-71338e16e973

c (**6**) mmol/L	*c* (TBA^+Cl^-) mmol/L	δ_{NH} (ppm)
6.52	0	9.2006
6.52	1.89	9.3230
6.52	3.71	9.4287
6.52	5.46	9.5259
6.52	8.77	9.6768
6.52	11.8	9.7841
6.52	16.1	9.9063
6.52	22.3	10.0412
6.52	27.6	10.1297
6.52	36.2	10.2379
6.52	42.9	10.3040
6.52	96.4	10.5564

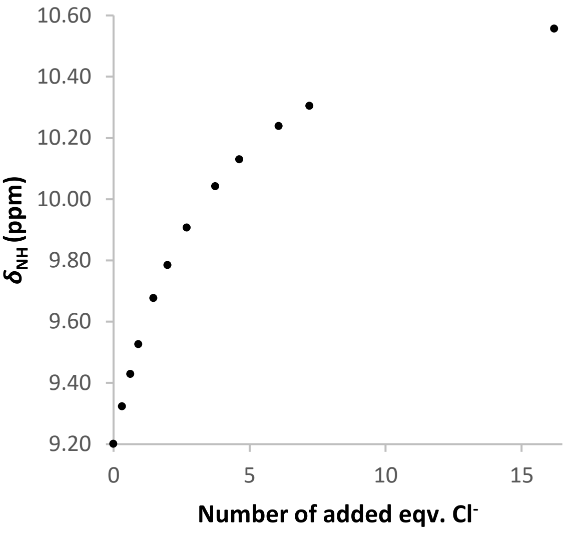

Figure S36: ^{1}H NMR titration of **6** by TBA^+Cl^- (DMSO-d_6, 400 MHz).

LINK: http://app.supramolecular.org/bindfit/view/b184952a-d02c-4879-9da7-d9594f819819

c (**6**) mmol/L	*c* ($TBA^+HSO_4^-$) mmol/L	δ_{NH} (ppm)
7.75	0	9.2238
7.75	2.86	9.2390
7.75	5.61	9.2549
7.75	8.25	9.2688
7.75	13.2	9.2931
7.75	17.9	9.3146
7.75	24.3	9.3406
7.75	33.6	9.3737
7.75	41.6	9.3996
7.75	54.6	9.4346
7.75	64.8	9.4598
7.75	146	9.5750

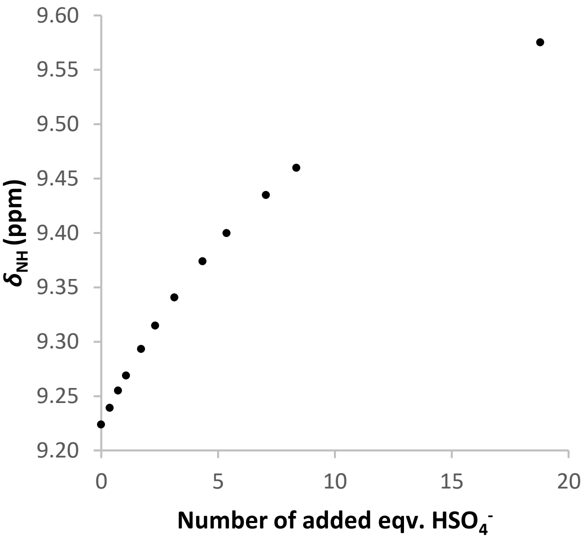

Figure S37: ^{1}H NMR titration of **6** by TBA^+ HSO_4^- (DMSO-d_6, 400 MHz).

LINK: http://app.supramolecular.org/bindfit/view/284a1ec7-8405-4171-a4df-8baaddf957fa

c (**6**) mmol/L	*c* (TBA^+Br^-) mmol/L	δ_{NH} (ppm)
8.22	0	9.2188
8.22	2.99	9.2368
8.22	5.86	9.2598
8.22	8.63	9.2837
8.22	13.9	9.3167
8.22	18.7	9.3445
8.22	25.4	9.3835
8.22	35.2	9.4335
8.22	43.6	9.4740
8.22	57.2	9.5274
8.22	67.7	9.5685
8.22	152	9.7808

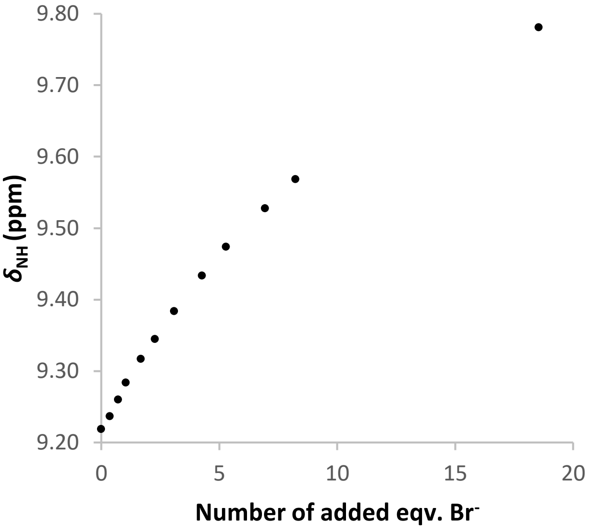

Figure S38: ^{1}H NMR titration of **6** by TBA^+Br^- (DMSO-d_6, 400 MHz).

LINK: http://app.supramolecular.org/bindfit/view/84957356-e4b6-4df6-aaf8-889a76d80aeb

c (**7**) mmol/L	c ($TBA^+H_2PO_4^-$) mmol/L	δ_{NH} (ppm)
0.400	0	9.3191
0.400	0.122	10.027
0.400	0.240	10.650
0.400	0.354	11.120
0.400	0.463	11.454
0.400	0.568	11.700
0.400	0.767	12.028
0.400	1.04	12.275
0.400	1.44	12.4668
0.400	1.78	12.5597
0.400	2.78	12.6561
0.400	6.25	12.8241

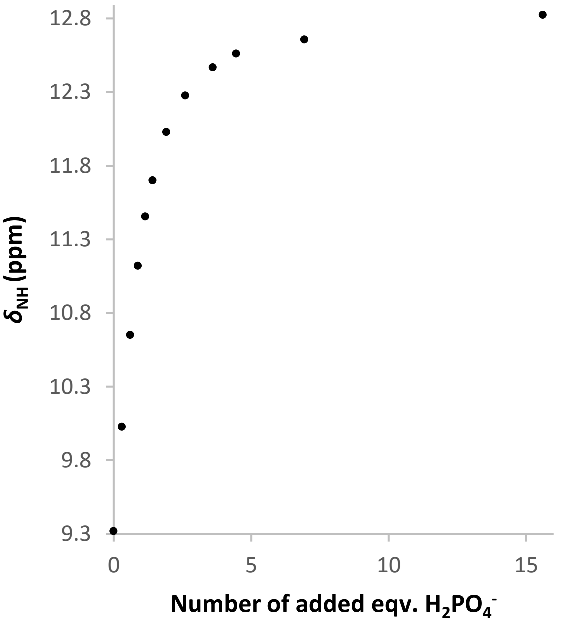

Figure S39: ^{1}H NMR titration of **7** by $TBA^+H_2PO_4^-$ (DMSO-d_6, 400 MHz).

LINK: http://app.supramolecular.org/bindfit/view/5134e79f-dc2d-4567-a8d7-703cff3e7e41

c (**7**) mmol/L	*c* (TBA^+BzO^-) mmol/L	δ_{NH} (ppm)
0.521	0	9.3243
0.521	0.194	9.8991
0.521	0.381	10.5691
0.521	0.560	11.0991
0.521	0.900	11.7291
0.521	1.22	12.0291
0.521	1.65	12.1591
0.521	2.28	12.3191
0.521	2.83	12.3791
0.521	3.71	12.4591
0.521	4.40	12.4791
0.521	9.90	12.5391

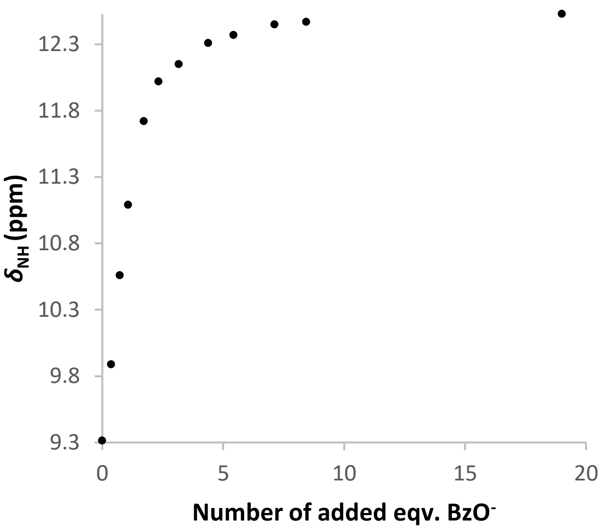

Figure S40: ^{1}H NMR titration of **7** by TBA^+BzO^- (DMSO-d_6, 400 MHz).

LINK: http://app.supramolecular.org/bindfit/view/c24ec3b3-2f27-4996-b3b5-1148cd4fa1ab

c (**7**) mmol/L	*c* (TBA^+Cl^-) mmol/L	δ_{NH} (ppm)
7.16	0	9.3313
7.16	2.14	9.4983
7.16	4.20	9.6340
7.16	6.18	9.7398
7.16	9.92	9.8988
7.16	13.4	10.0119
7.16	18.2	10.1299
7.16	25.2	10.2509
7.16	31.2	10.3268
7.16	40.9	10.4126
7.16	48.5	10.4605
7.16	109	10.6494

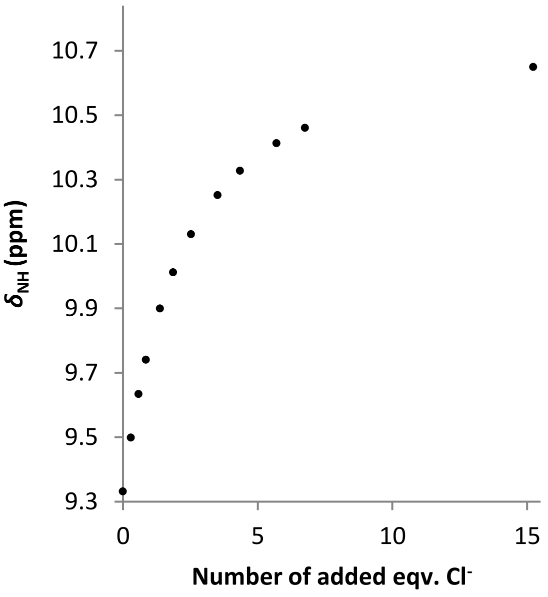

Figure S41: ^{1}H NMR titration of **7** by TBA^+Cl^- (DMSO-d_6, 400 MHz).

LINK: http://app.supramolecular.org/bindfit/view/7a427375-8365-44e7-b551-595868648192

c (**7**) mmol/L	c ($TBA^+HSO_4^-$) mmol/L	δ_{NH} (ppm)
8.97	0	9.3458
8.97	2.45	9.3769
8.97	4.81	9.3982
8.97	7.07	9.4163
8.97	11.4	9.4467
8.97	15.3	9.4720
8.97	20.8	9.5052
8.97	28.8	9.5460
8.97	35.7	9.5747
8.97	46.9	9.6145
8.97	55.5	9.6415
8.97	125	9.7631

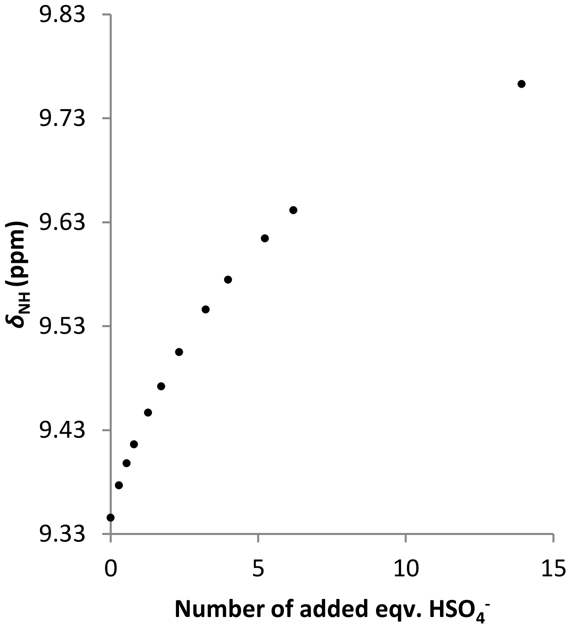

Figure S42: ^1H NMR titration of **7** by $TBA^+HSO_4^-$ (DMSO-d_6, 400 MHz).

LINK: http://app.supramolecular.org/bindfit/view/fa380b77-67f6-4da2-9909-cdc0ee9a86e0

c (**7**) mmol/L	*c* (TBA^+Br^-) mmol/L	δ_{NH} (ppm)
8.76	0	9.3389
8.76	2.41	9.3572
8.76	4.72	9.3735
8.76	6.95	9.3920
8.76	11.2	9.4211
8.76	15.1	9.4471
8.76	20.5	9.4803
8.76	28.3	9.5229
8.76	35.1	9.5568
8.76	46.0	9.6069
8.76	54.5	9.6327
8.76	123	9.8373

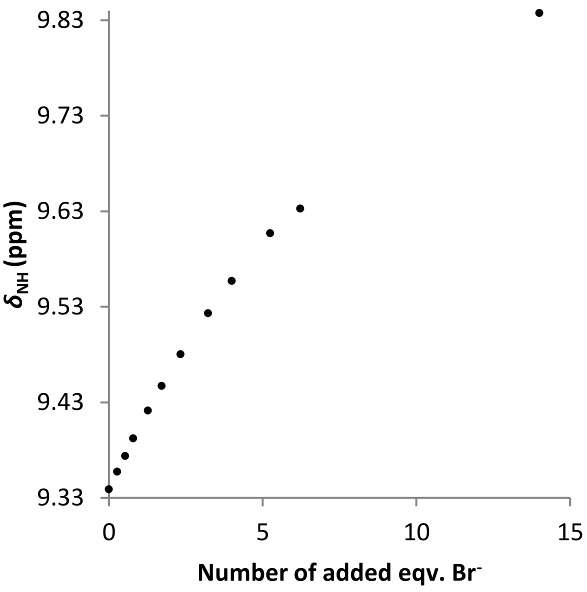

Figure S43: ^{1}H NMR titration of **7** by TBA^+Br^- (in DMSO-*d*$_6$, 400 MHz).

LINK: http://app.supramolecular.org/bindfit/view/860eaf38-d0ce-4e52-acbc-b1d1198e8076

c (**8**) mmol/L	*c* (TBA^+BzO^-) mmol/L	eqv.	δ_{NH} (ppm)
1.25	0	0	9.4 (brs)
1.25	0.291	0.1	9.406
1.25	0.571	0.33	9.443
1.25	0.841	0.56	9.462
1.25	1.10	0.77	9.559
1.25	1.35	0.98	9.716
1.25	1.82	1.18	9.829
1.25	2.48	1.60	10.188
1.25	2.87	2.08	10.659
1.25	3.25	2.40	10.932
1.25	3.93	2.70	11.181
1.25	4.24	3.25	11.562
1.25	4.95	3.49	11.726
1.25	5.57	4.06	11.970
1.25	6.12	4.56	12.110
1.25	6.60	4.99	12.177
1.25	14.9	5.38	12.236

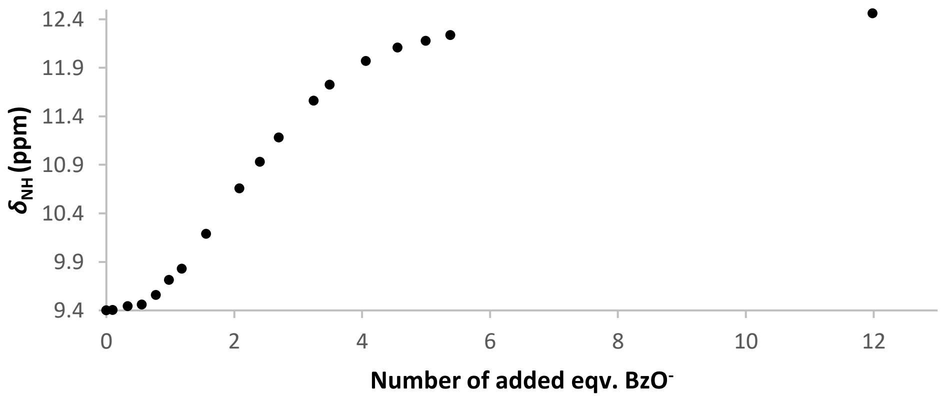

Figure S44: ^{1}H NMR titration of dendrimer **8** (1.25 mM, $CDCl_3$) with TBA^+BzO^-.

8 Extraction

Time dependence

Table S3: Time dependence of phase transfer of $TBA^+H_2PO_4^-$ from aqueous solution into organic phase containing receptor **7.**

τ	Integral ratio for CH_3 signals (**7** : TBA^+)	c (mM)[a]	NH shifts (ppm)	
			NH_a	NH_b
1 min	1 : 2.3	2.9	9.91	9.68
5 min	1 : 2.3	2.9	9.91	9.68
15 min	1 : 2.3	2.9	9.91	9.68
60 min	1 : 2.2	2.8	9.89	9.65
Diffusion 24 h	1 : 2.1	2.6	9.84	9.60

Conditions: c_{aq} ($TBA^+H_2PO_4^-$) = 10 mM, c_{org}(**7**) = 5 mM; [a] Extracted concentration of anion was evaluated as a ratio between the integral obtained for CH_3 ***I*** (from TBA^+, related to CH_3 signal of receptor **7** - ***II***) and its theoretical maximum value, multiplied by concentration of **7**.

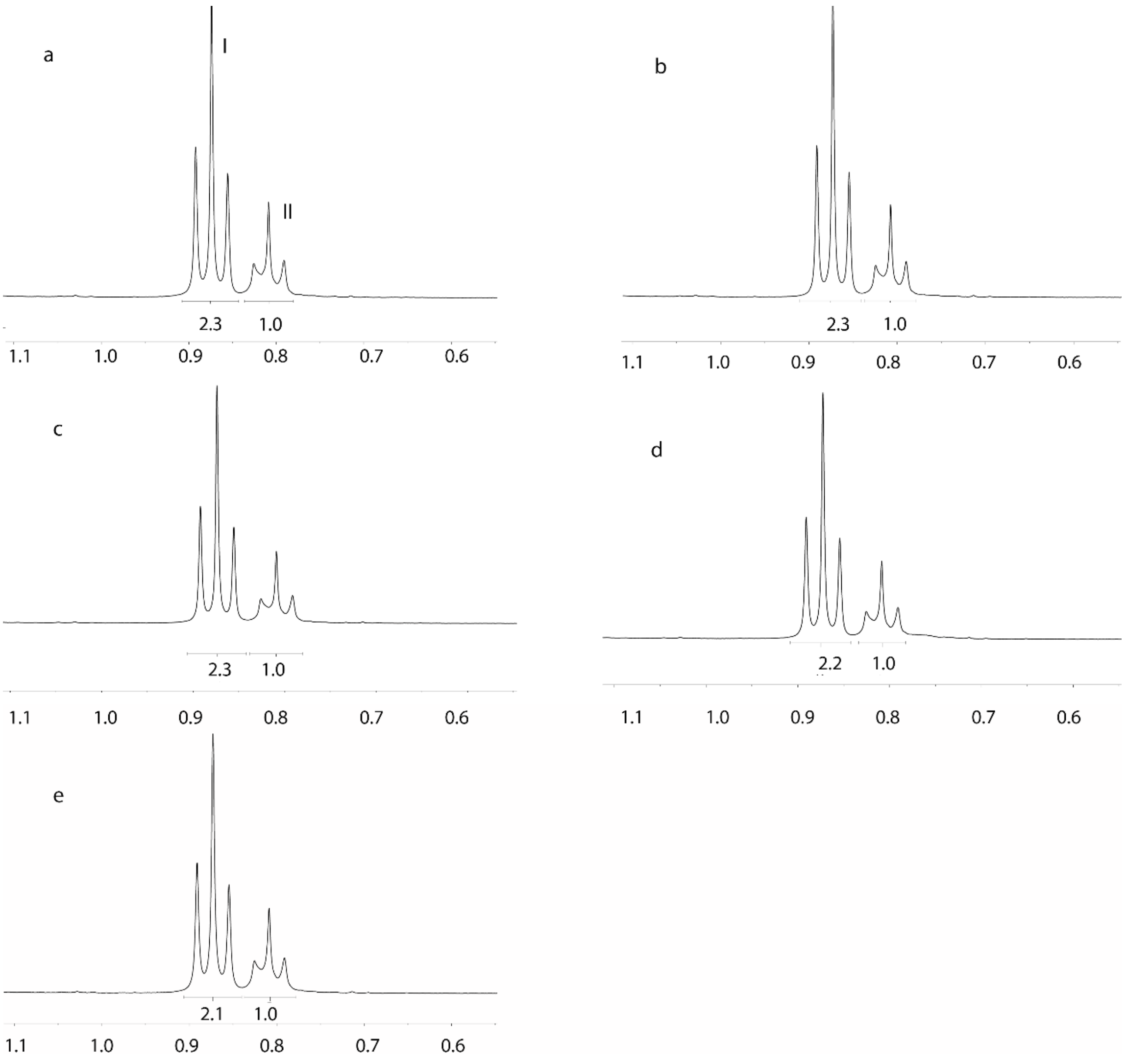

Figure S45: ^{1}H NMR of **7**, CH_3 groups; ***I***: CH_3 of TBA^+ salt, ***II***: CH_3 of receptor **7**, extraction of aqueous phase containing 10 mM $TBA^+H_2PO_4^-$ with extraction time **a)** 1 min **b)** 5 min **c)** 15 min **d)** 60 min **e)** without shaking – free diffusion for 24 hours.

Concentration dependence

Table S4: Concentration dependence of phase transfer of $TBA^+H_2PO_4^-$ from aqueous solution into organic phase containing receptor **7.**

c_{aq} ($H_2PO_4^-$) mM	Integral ratio for CH_3 signals (**7** : TBA^+)	*c* (mM) [a]	NH shifts (ppm)	
			NH_a	NH_b
0	-	-	~ 7.5 (brs)	
5	1 : 1.9	2.4	9.65	9.43
10	1 : 2.3	2.9	9.91	9.68
25	1 : 2.7	3.4	10.10	9.87
100	1 : 3.8	4.8	10.24	10.01

Conditions: c_{org}(**7**) = 5 mM, τ = 15 min; [a] Extracted concentration was evaluated as a ratio between the integral obtained for CH_3 ***I*** (from TBA^+ related to CH_3 signal of receptor **7** - ***II***) and its theoretical maximum value, multiplied by concentration of **7**.

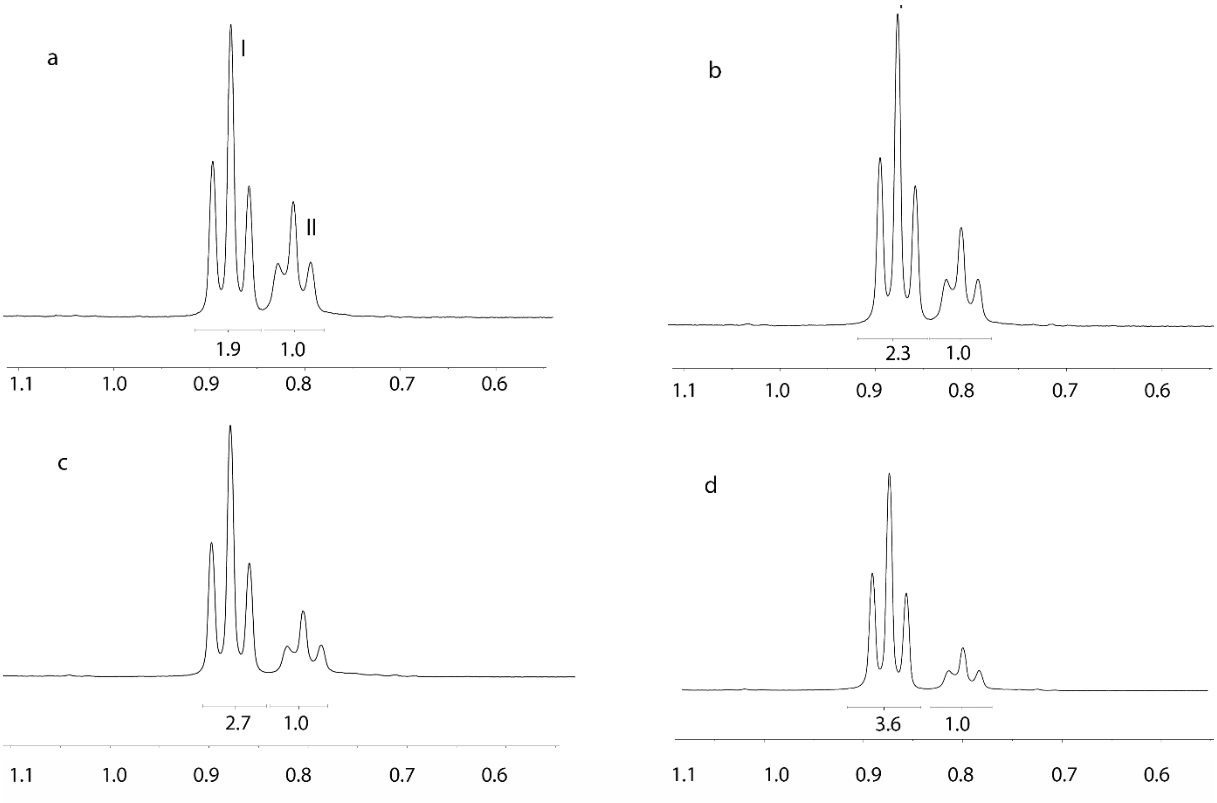

Figure S46: ^{1}H NMR of 7, CH_3 groups; signal ***I***: CH_3 of TBA^+ salt, ***II***: CH_3 of receptor **7**, extraction of aqueous phase containing $TBA^+H_2PO_4^-$ **a)** 5 mM **b)** 10 mM **c)** 25 mM **d)** 100 mM.

pH dependence

Table S5: Phase transfer of different forms of phosphates from aqueous solution into organic phase containing receptor **7.**

Form of anion	pH	Integral ratio for CH_3 signals (**7** : TBA^+)	*c* (mM) [a]	NH shifts (ppm)	
				NH_a	NH_b
$H_2PO_4^-$	4.7	1 : 2.3	2.9	9.91	9.67
HPO_4^{2-}	10.4	1 : 3.3	2.1	10.10	9.86

Conditions: : c_{aq} ($TBA^+H_2PO_4^-$) or ($TBA_2^+HPO_4^-$) = 10 mM, c_{org}(**7**) = 5 mM, τ = 15 min; [a] Extracted concentration was evaluated as a ratio between the integral obtained for CH_3 ***I*** (from TBA^+ related to CH_3 signal of receptor **7** - ***II***) and its theoretical maximum value, multiplied by concentration of **7**.

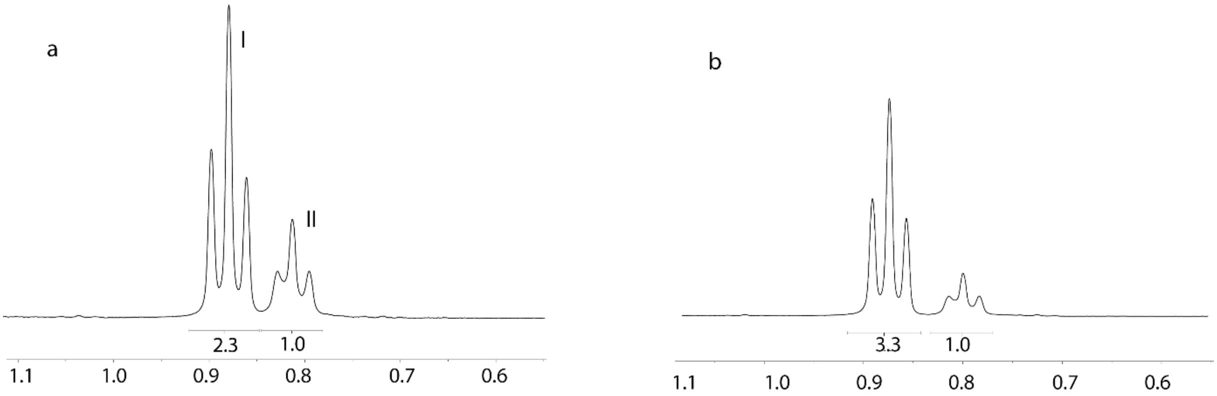

Figure S47.^{1}H NMR of **7**, CH_3 groups: signal ***I***: CH_3 of TBA^+ salt, ***II***: CH_3 of receptor **7** after extraction of aqueous phase containing 10 mM of **a)** $TBA^+H_2PO_4^-$ **b)** $TBA_2^+HPO_4^{2-}$.

Extraction of different anions

Table S6: The phase transfer of different TBA^+ salts from aqueous solution into organic phase containing receptor **7**.

Anion	Self-transfer *c* (mM) [a]	Integral ratio for signals (**7** : TBA^+)	*c* (mM) [b]	NH shift (ppm)	
				NH_a	NH_b
BzO^-	0.4	1 : 2.7 (CH_2)	3.4	10.53	10.33
HSO_4^-	-	1 : 2.2 (CH_2)	2.8	10.04	9.85
Cl^-	0.1	1 : 2.4 (CH_3)	3.0	9.94	9.70
$H_2PO_4^-$	-	1 : 2.3 (CH_3)	2.9	9.91	9.67
Br^-	1.5	1 : 3.7 (CH_3)	4.6	9.86	9.66
Free	-	-	-	~ 7.5 (brs)	

Conditions: $c_{aq}(TBA^+X^-)$ = 10 mM, $c_{org}(\mathbf{7})$ = 5 mM, τ = 15 min; [a] Phase transfer of corresponding TBA^+ salt (10 mM) from water to $CDCl_3$ phase in absence of receptor **7**; [b] Extracted concentration was evaluated as a ratio between the integral obtained for CH_x (from TBA^+ related to signal of receptor **7**) and its theoretical maximum value, multiplied by concentration of **7**.

Table S7: The phase transfer of different TBA^+ salts from aqueous solution into organic phase containing dendrimer **8**.

Anion	Precipitation [a]	*c* (mM) [b]	NH shift (ppm)	
BzO^-	-	4.9	11.23; 11.13	11.01; 10.93
HSO_4^-	~ 60 % [c]	-	10.17	10.07
Cl^-	~ 20 % [d]	2.5	9.69; 9.65	9.46; 9.43
$H_2PO_4^-$	~ 20 % [d]	1.5	9.42	9.24
Br^-	-	4.0	9.66	9.46
Free + water	-	-	8.51	8.35

Conditions: $c_{aq}(TBA^+X^-)$ = 10 mM, $c_{org}(\mathbf{8})$ = 1.25 mM, τ = 15 min; [a] Precipitation was evaluated by comparing the ratio of solvent signal and signals of dendrimer **8**. [b] Extracted concentration was evaluated as a ratio between the integral obtained for CH_x (from TBA^+ related to signals of dendrimer **8**) and its theoretical maximum value, multiplied by concentration of **8**. [c] The precipitate was isolated, dissolved and analysed in DMSO-d_6, as containing 2.5 mM of TBA^+ HSO_4^-. [d] precipitate not isolated.

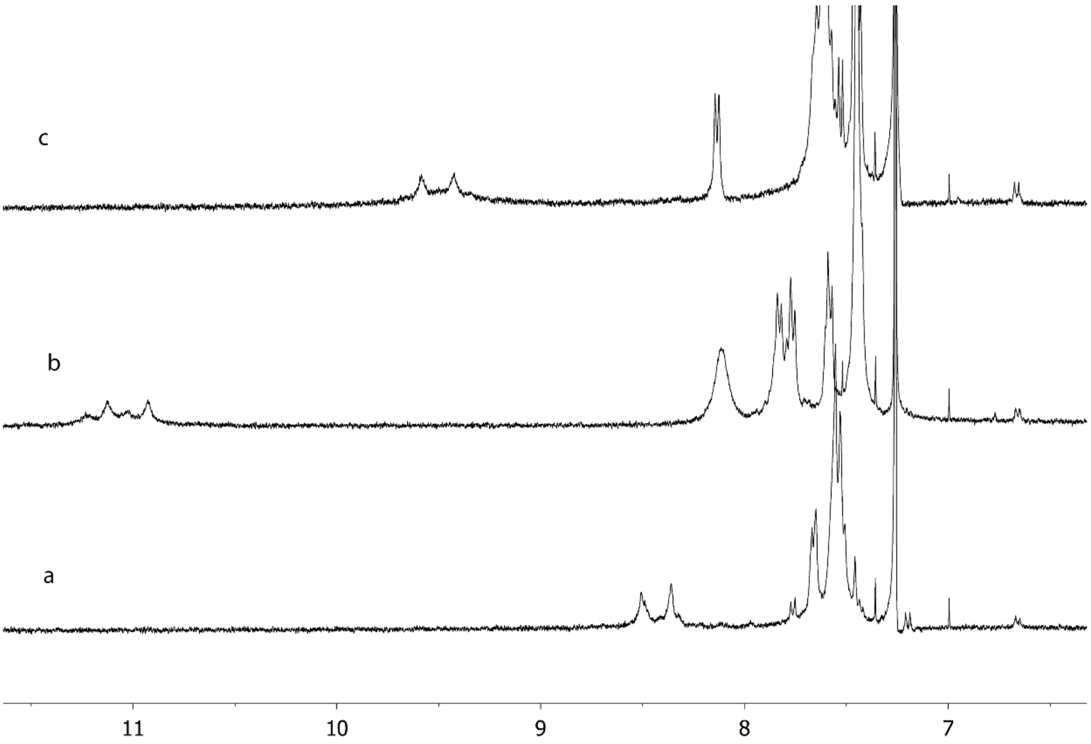

Figure S48: Part of aromatic and NH region in ^{1}H NMR spectra of dendrimer **8** (1.25 mM, wet $CDCl_3$) **a)** without extracting anion **b)** the same solution of dendrimer **8** after shaking with 10 mM aqueous solution of TBA^+BzO^-; and **c)** spectrum after back-extraction of chloroform solution b with water (3 x 1.5 mL).

Table S8: The phase transfer of different TBA^+ carboxylates from aqueous solution into organic phase containing dendrimer **8**.

Anion	*c* (mM) [a]	NH shift (ppm)	
BzO^-	~ 4.9	11.17; 11.06	10.96; 10.86
Phtalate	~ 2.7[b]	10.48	10.37
Isophtalate	~ 1.4[b]	11.19	11.05
Terephtalate	~ 1.3[b]	10.94	10.75

Conditions: $c_{aq}(TBA_x^+X^-)$ = 10 mM, $c_{org}(\mathbf{8})$ = 1.25 mM, τ = 15 min; Extracted concentration was evaluated as a ratio between the integral obtained for CH_x (from TBA^+) and its theoretical maximum value, multiplied by concentration of **8**, related to receptor concentration in chloroform: [b] precipitation of dendrimer about 30 %; the amount of anion in the precipitate was neglected.

Anion mixtures

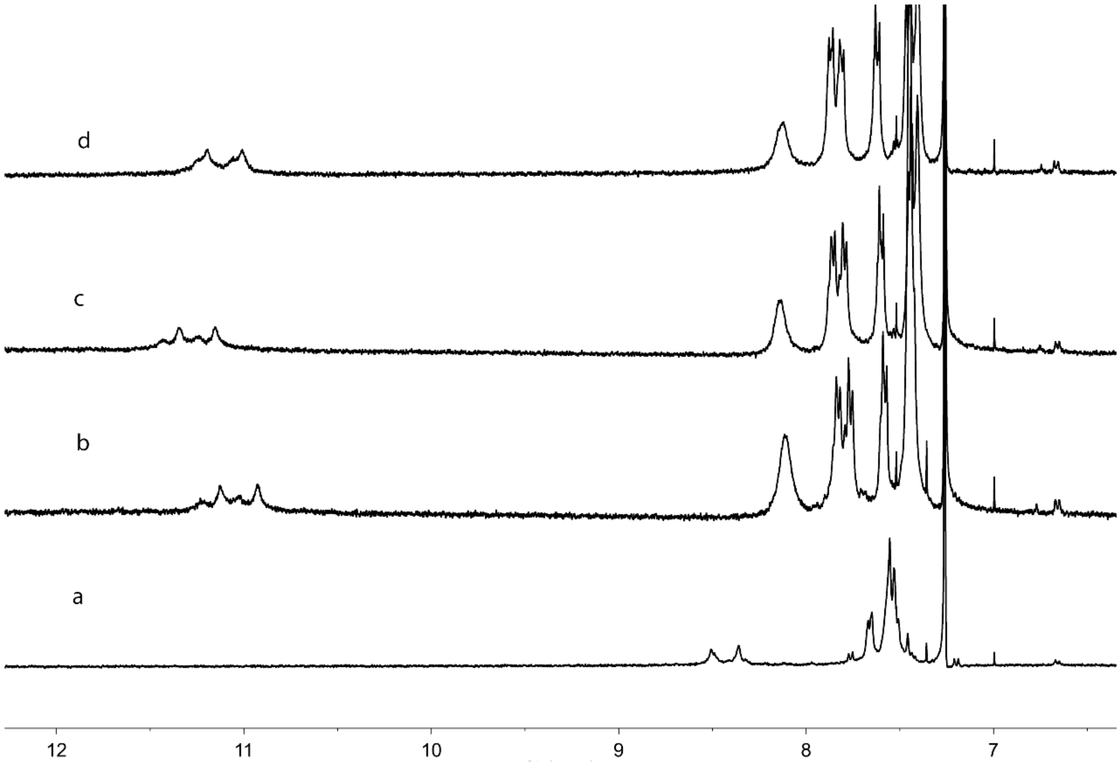

Figure S49: Part of aromatic and NH region in ^{1}H NMR spectra of dendrimer **8** (1.25 mM, wet $CDCl_3$) **a)** without extracting anion **b)** after extraction of 10 mM aqueous solution of TBA^+BzO^- **c)** after shaking with 10 mM aqueous solution of TBA^+BzO^- containing additionally 10 mM of TBA^+Cl^- **d)** after shaking with 10 mM aqueous solution of TBA^+BzO^- containing additionally 10 mM concentration of TBA^+Br^-.

Table S9: The phase transfer of benzoate from aqueous solution mixtures into organic phase containing dendrimer **8**.

Concentration of anion in aqueous phase before extraction			Integral ratio for signals (**8** : BzO^-) [a]	c_{ex} BzO^- (mM) [b]
c_{aq0} (TBA^+BzO^-) mM	c_{aq0} (TBA^+Cl^-) mM	c_{aq0} (TBA^+Br^-) mM		
10	-	-	8 : 7.86	~ 4.9
10	10	-	8 : 4.72	~ 3.0
10	-	10	8 : 7.12	~ 4.5

Conditions: Content of aqueous phase is determined in the table, c_{org}(**8**) = 1.25 mM, τ = 15 min; Extracted concentration was evaluated as a ratio between the integral obtained for benzoate CH and its theoretical maximum value, multiplied by concentration of **8**.

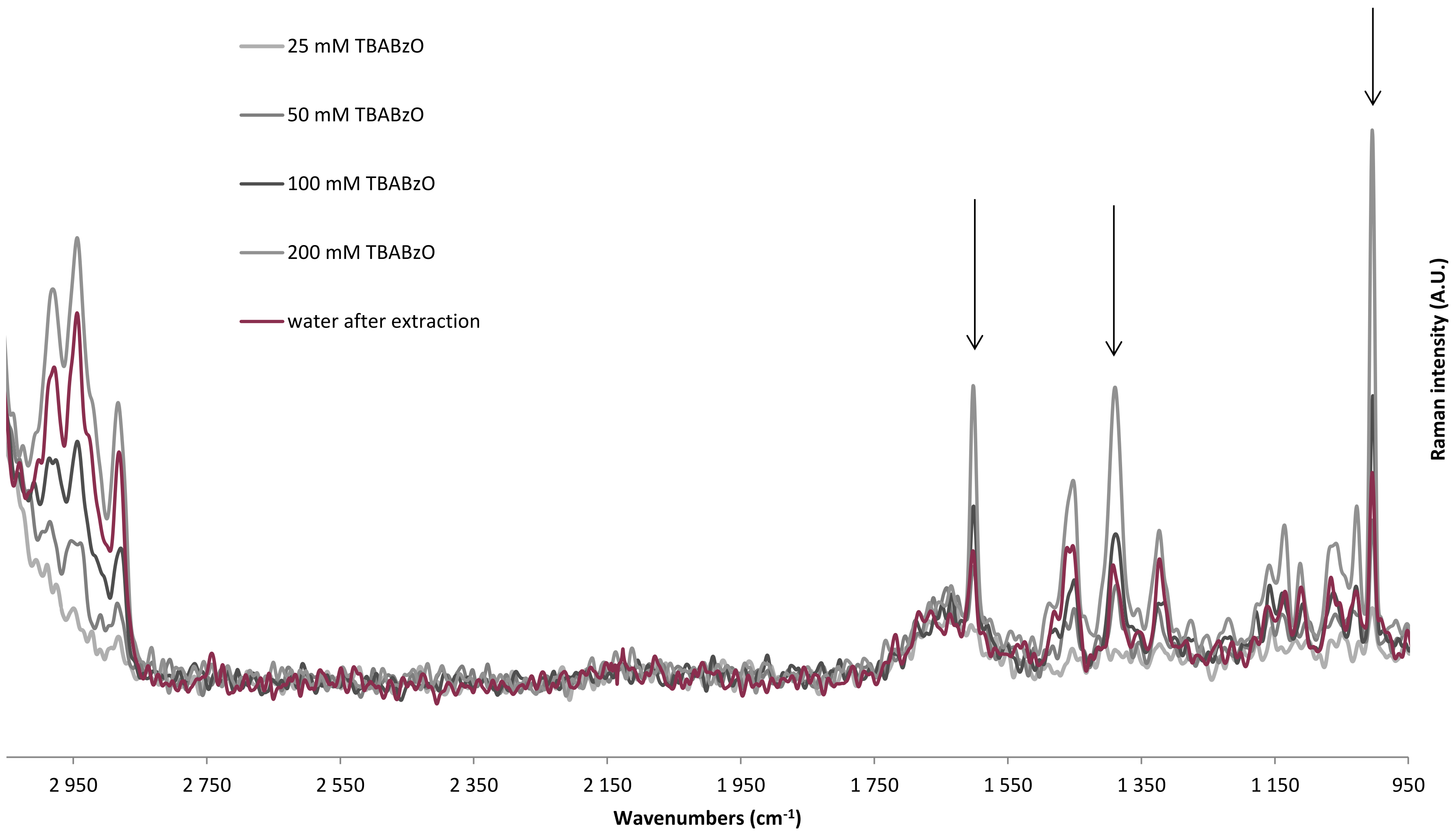

Figure S50: A series of Raman spectra **a)** grades of grey: containing different concentrations of TBA^+BzO^- in water **b)** red: aqueous phase after extraction. Arrows indicating clearly distinguishable bands corresponding to BzO^-.

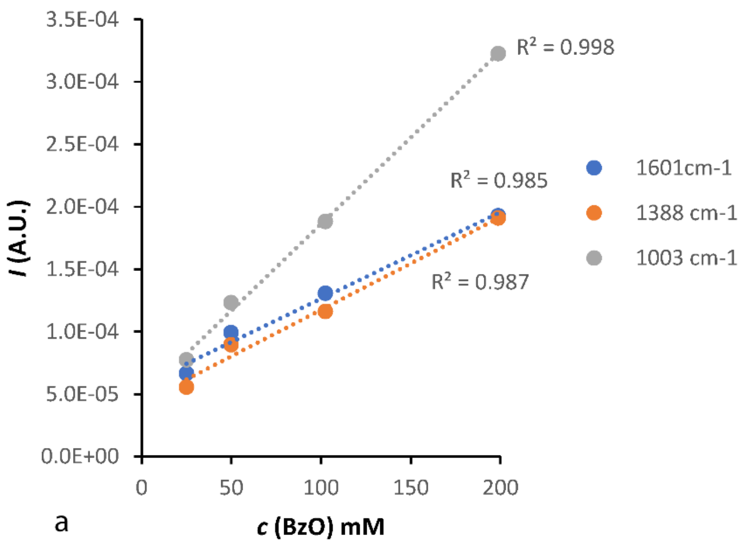

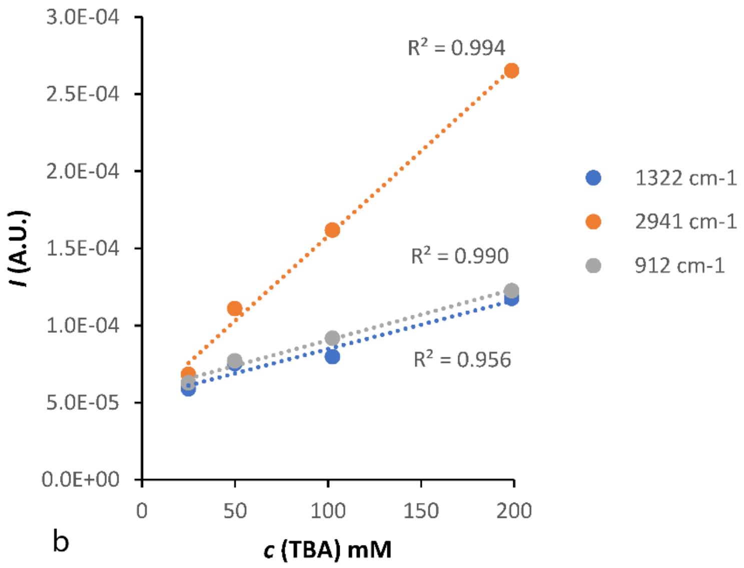

Figure S51. Raman spectroscopy. The calibration curves for calculation of the content of TBA BzO in the presence of TBA Cl in the water phase after extraction with chloroform containing dendrimer 8; a) concentration of benzoate anions, b) concentration of tetrabutylammonium cations, corresponding to concentration of both benzoate and chloride anions.

Table S10: The values of Raman intensity (with corresponding wavenumbers) of the aqueous phase containing TBA BzO and TBA Cl, after extraction with organic phase ($CDCl_3$ solution of dendrimer **8**). The table also contains lines parameters *a* and *b* obtained from calibration curves (S51 a), and calculated values of concentrations of benzoate in water after extraction.

Signal	Wavenumber $\tilde{\nu}$ (cm^{-1})	*I* (A.U.) after extraction	*a*	*b*	$c_{calculated}$ (mM)
BzO^-	1 600	1.03E-04	6.60E-07	5.73E-05	68.8
	1 388	9.36E-05	7.40E-07	4.32E-05	68.2
	1 004	1.42E-04	1.40E-06	4.58E-05	68.3
	Spectral intervals of BzO^-	TQ Analyst	65.5E-07	4.58E-05	68.3

Table S11: The values of Raman intensity (with corresponding wavenumbers) of the aqueous phase containing TBA BzO and TBA Cl, after extraction with organic phase ($CDCl_3$ solution of dendrimer **8**). The table also contains lines parameters *a* and *b* obtained from calibration curves (S51 b), and calculated values of concentrations of TBA^+ in water after extraction.

Signal	Wavenumber $\tilde{\nu}$ (cm^{-1})	*I* (A.U.) after extraction	*a*	*b*	$c_{calculated}$ (mM)
TBA^+	2 941	2.24E-04	1.10E-06	4.81E-05	156.7
	1 322	1.03E-04	3.20E-07	5.32E-05	156.0
	911	1.08E-04	3.30E-07	5.74E-05	155.2

9 Computational studies

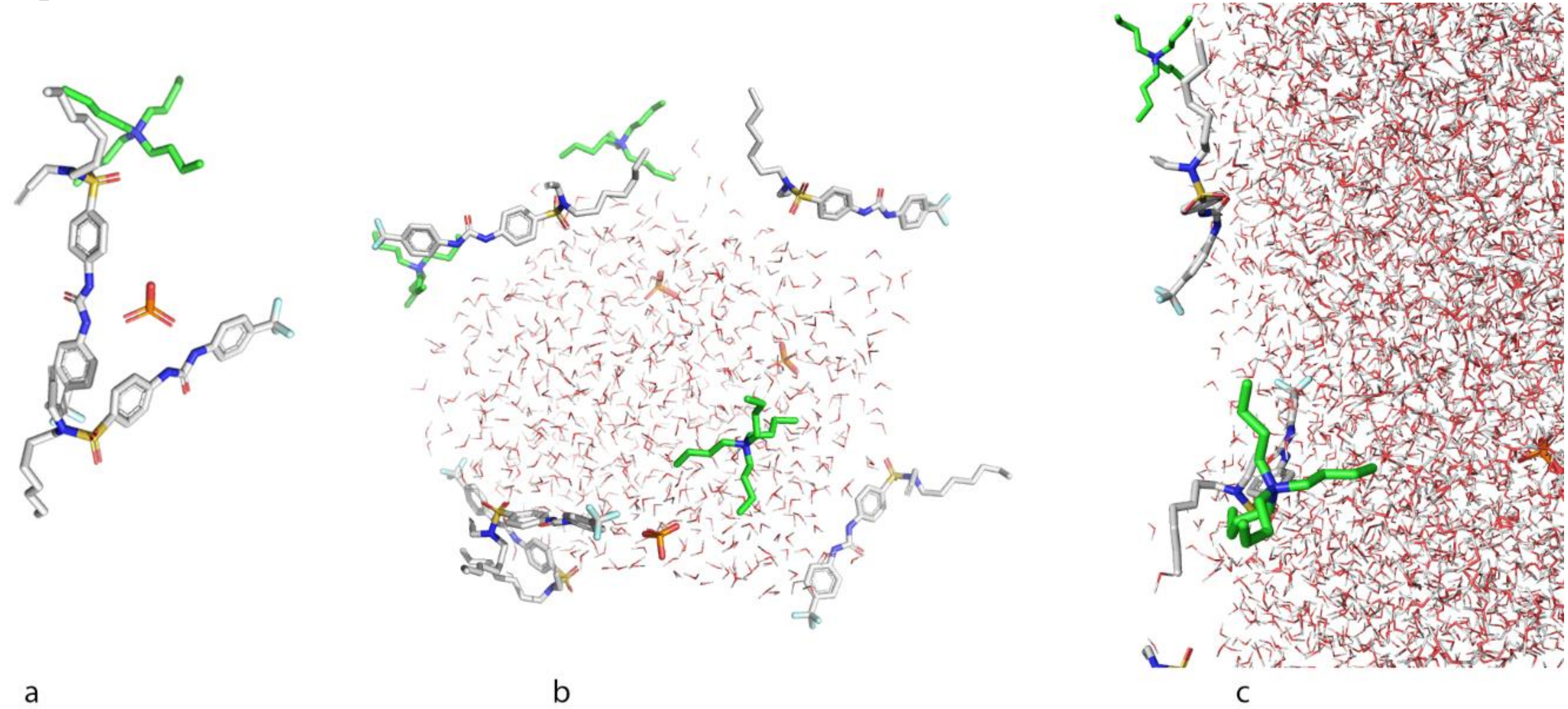

Figure S52, Simulation of behaviour of **7** in the presence of TBA$^+$ $H_2PO_4^-$; a) dry chloroform, b) wet chloroform, c) water/chloroform interface.

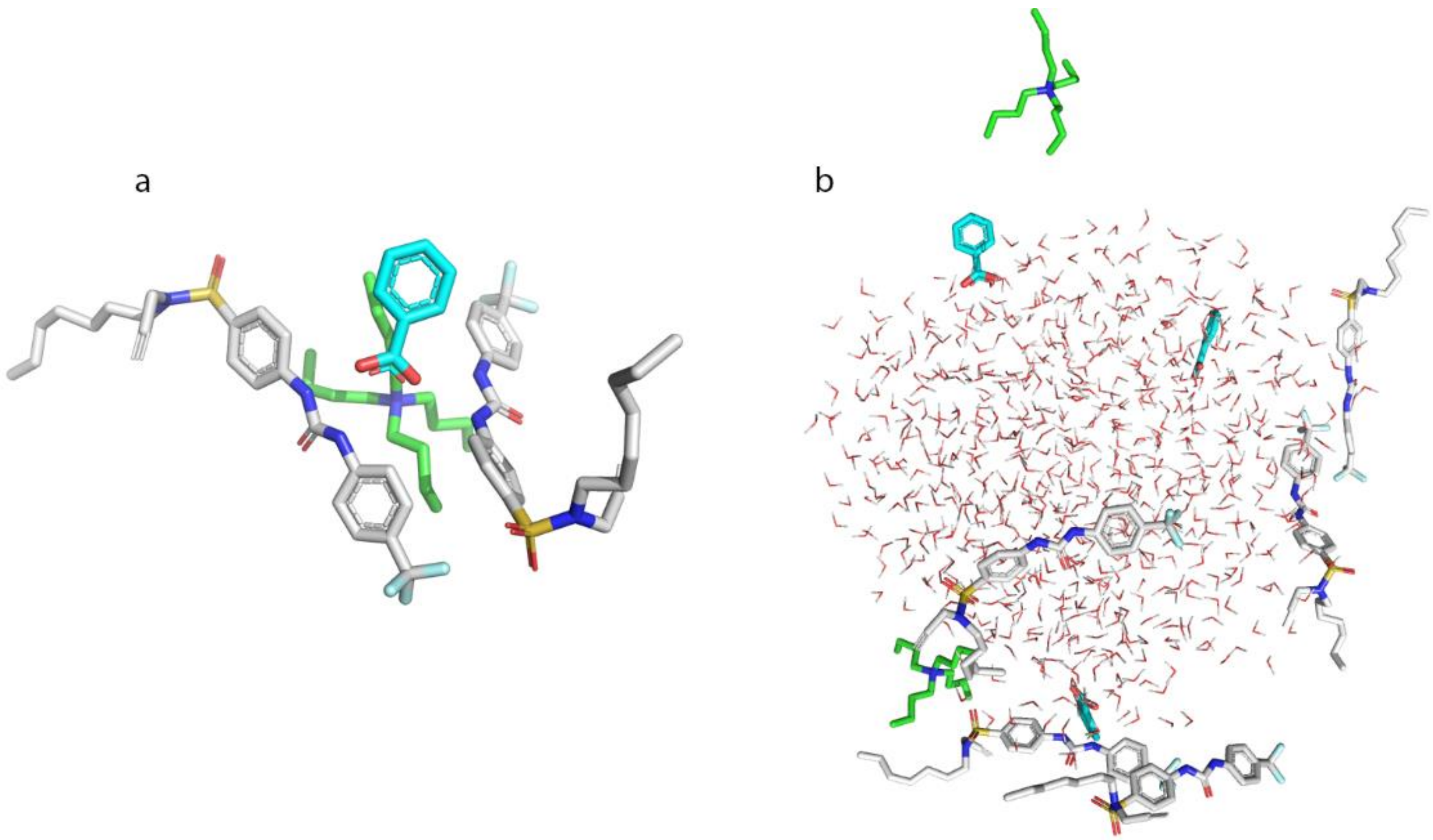

Figure S53. Simulation of behaviour of **7** in the presence of TBA^+ BzO^-; a) chloroform, b) wet chloroform.

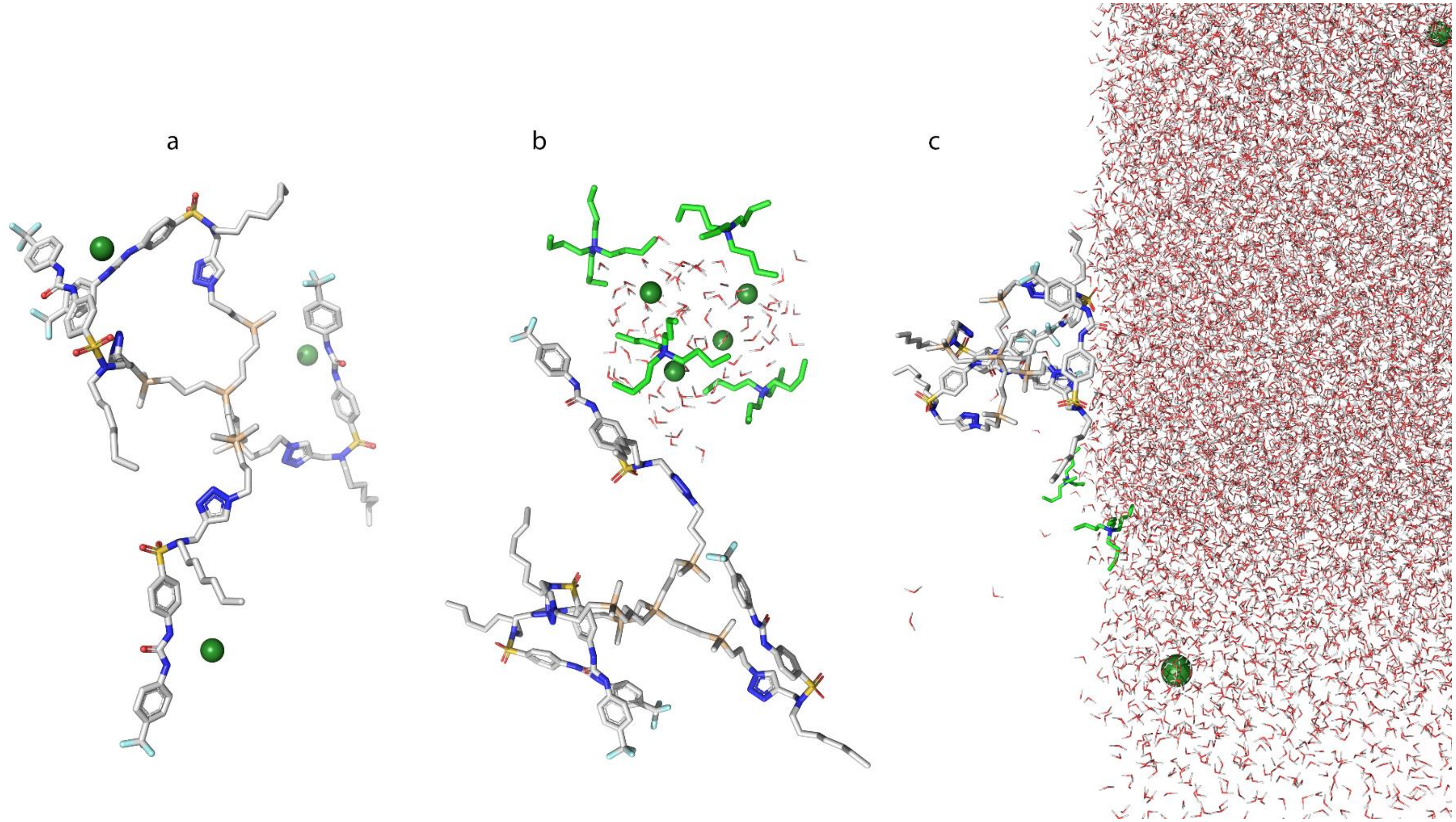

Figure S54. Simulation of behaviour of **8** in the presence of TBA^+ Cl^-; a) dry chloroform, b) wet chloroform, c) water/chloroform interface.

10 References

(S1) CrysAlis PRO. Rigaku Oxford Diffraction Ltd, Yarnton, Oxfordshire, England, **2020**.

(S2) APEX4, SAINT and SADABS. Bruker AXS Inc., Madison, Wisconsin, USA, **2021**.

(S3) Palatinus L.; Chapuis G. SUPERFLIP–a computer program for the solution of crystal structures by charge flipping in arbitrary dimensions. *J. Appl. Cryst.* **2007**, *40*, 786-790. https://doi.org/10.1107/S0021889807029238

(S4) Betteridge P. W.; Carruthers J.R.; Cooper R. I.; Prout K.; Watkin D. J. Software for guided crystal structure analysis. *J. Appl. Cryst.* **2003**, *36*, 1487. http://dx.doi.org/10.1107/S0021889803021800

(S5) Rohlicek J.; Husak M. MCE2005–a new version of a program for fast interactive visualization of electron and similar density maps optimized for small molecules. *J. Appl. Cryst.* **2007**, *40*, 600. https://doi.org/10.1107/S0021889807018894

(S6) Brandenburg, K. DIAMOND. Crystal Impact GbR, Bonn, Germany, **1999**.

(S7) Macrae C. F.; Edgington P. R.; McCabe P.; Pidcock E.; Shields G. P.; Taylor R.; Towler M.; van de Streek J. Mercury: Visualization and Analysis of Crystal Structures. *J. Appl. Cryst.* **2006**, *39*, 453-457. http://dx.doi.org/10.1107/S002188980600731X

(S8) Krupková A.; Müllerová M.; Petričkovič R.; Strašák T. On the edge between organic solvent nanofiltration and ultrafiltration: Characterization of regenerated cellulose membrane with aspect on dendrimer purification and recycling. *Sep. Purif. Technol*. **2023**, 123141. https://doi.org/10.1016/j.seppur.2023.123141

(S9) Chemical Computing Group, I. Molecular Operating Environment (MOE 2019.10). Available from: http://www.chemcomp.com.

(S10) Schrödinger Suite, Release 2021-2, Schrödinger, LLC New York, NY, USA. Available from: https://www.schrodinger.com

(S11) Salvadori K.; Šimková L.; Císařová I.; Sýkora J.; Ludvík J.; Cuřínová P. Sulphonamidic Groups as Electron-Withdrawing Units in Ureido-Based Anion Receptors: Enhanced Anion Complexation versus Deprotonation. *ChemPlusChem* 2020, *85*, 1401-1411. https://doi.org/10.1002/cplu.202000326.

(S12) Kraljević T. G.; Harej A.; Sedić M.; Pavelić S. K.; Stepanić V.; Drenjančević D.; Talapko, J.; Raić-Malić S.; Synthesis, in vitro anticancer and antibacterial activities and in silico studies of new 4-substituted 1,2,3-triazole–coumarin hybrids. *Eur. J. Med. Chem.* **2016**, *124, 794–808.* https://doi:10.1016/j.ejmech.2016.08.062

(S13) Von Krbek, L. K. S., Schalley, C. A., & Thordarson, P. Assessing cooperativity in supramolecular systems. Chemical Society Reviews **2017**, *46*, 2622–2637. https://doi:10.1039/c7cs00063d
